# Ionic Associations and Hydration in the Electrical Double Layer of Water-in-Salt Electrolytes

**DOI:** 10.1021/acsami.5c01781

**Published:** 2025-05-07

**Authors:** Daniel M. Markiewitz, Zachary A. H. Goodwin, Qianlu Zheng, Michael McEldrew, Rosa M. Espinosa-Marzal, Martin Z. Bazant

**Affiliations:** † Department of Chemical Engineering, 2167 Massachusetts Institute of Technology, Cambridge, Massachusetts 02139, United States; ‡ John A. Paulson School of Engineering and Applied Sciences, Harvard University, Cambridge, Massachusetts 02138, United States; § Department of Materials, 170993 University of Oxford, Parks Road, Oxford OX1 3PH, U.K.; ∥ Department of Civil and Environmental Engineering, The Grainger College of Engineering, 14589 University of Illinois Urbana−Champaign, Urbana, Illinois 61801, United States; ⊥ Department of Materials Science and Engineering, The Grainger College of Engineering, 14589 University of Illinois Urbana−Champaign, Urbana, Illinois 61801, United States; # Department of Mathematics, 2167 Massachusetts Institute of Technology, Cambridge, Massachusetts 02139, United States

**Keywords:** chemical thermodynamics, electric double layer, interfacial properties, concentrated electrolytes, water-in-salt electrolytes

## Abstract

Water-in-Salt-Electrolytes (WiSEs) are an exciting class of concentrated electrolytes finding applications in energy storage devices because of their expanded electrochemical stability window, good conductivity and cation transference number, and fire-extinguishing properties. These distinct properties are thought to originate from the presence of an anion-dominated ionic network and interpenetrating water channels for cation transport, which indicates that associations in WiSEs are crucial to understanding their properties. Currently, associations have mainly been investigated in the bulk, while little attention has been given to the electrolyte structure near electrified interfaces. Here, we develop a theory for the electrical double layer (EDL) of WiSEs, where we consistently account for the thermoreversible associations of species into Cayley tree aggregates. The theory predicts an asymmetric structure of the EDL. At negative voltages, hydrated Li^+^ dominates, and cluster aggregation is initially slightly enhanced before disintegration at larger voltages. At positive voltages, when compared to the bulk, clusters are strictly diminished. Performing atomistic molecular dynamics (MD) simulations of the EDL of WiSE provides EDL data for validation and bulk data for parametrization of our theory. Validating the predictions of our theory against MD showed good qualitative agreement. Furthermore, we performed electrochemical impedance measurements to determine the differential capacitance of the studied LiTFSI WiSE and also found reasonable agreement with our theory. Overall, the developed approach can be used to investigate ionic aggregation and solvation effects in the EDL, which, among other properties, can be used to understand the precursors for solid-electrolyte interphase formation.

## Graphical abstract

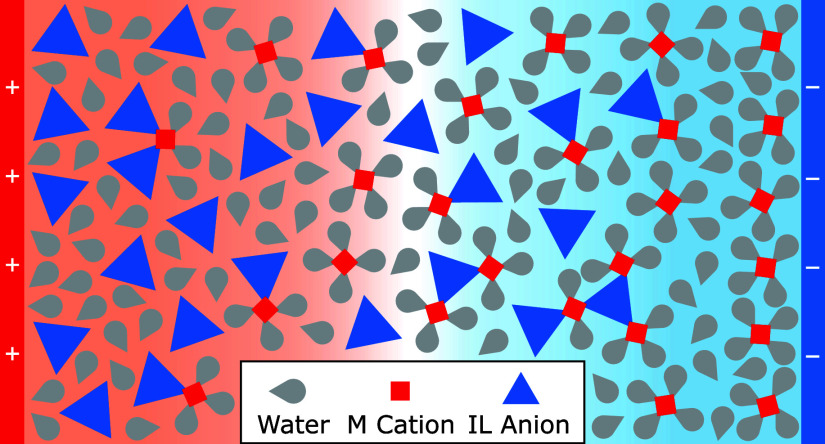

## Introduction

Water-in-Salt Electrolytes (WiSEs) have emerged as a promising class of electrolytes for applications in batteries and supercapacitors.^1–7^ In contrast to conventional organic Li-ion battery electrolytes, WiSEs have dramatically improved safety and stability, owing to the use of water as a solvent instead of flammable carbonate solvents.^1,2,6,7^ In classical dilute aqueous electrolytes, water is known to electrolyze around 1.23 V, giving their small electrochemical stability windows (ESW), but the superconcentrated WiSE regime displays enhanced ESWs up to 4 V.^5^ The reductive stability of WiSEs is attributed to the formation of a passivating solid-electrolyte interphase (SEI) at the anode, similar to conventional Li-ion battery electrolytes.^2,8,9^ At the same time, the oxidative stability originates from the thermodynamic activity of water reducing in the superconcentrated regime.^8–12^

As WiSEs are often used in the superconcentrated salt regime, such as 21m LiTFSI, it is perhaps not surprising that the aggregation of ions has been discovered to be important in numerous simulation and experimental studies.^12–19^ At the optimal concentration of 21m LiTFSI, the electrolyte obtained an operating voltage of ∼2.3 V while maintaining reasonable conductivity.^2^ They found that a predominantly Li-anion ionic network exists^13^ that is interpenetrated by nanochannels of water-rich domains containing Li cations.^12,13,15^ The existence of these nanochannels enables the facile transport of Li cations, which is crucial for their operating performance. In addition, the existence and equilibrium of aggregates at interfaces have been revealed by surface force apparatus (SFA) and atomic force microscopy (AFM) measurements^18–20^ and molecular dynamics simulations,^10,11,21^ where it was found that hydrated Li^+^ exists near the interface.

To understand these simulations and experiments in the electrical double layer (EDL) of WiSEs, it is useful to have a theory to rationalize the observations. One of the first theories for the EDL of WiSEs came from McEldrew et al.,^11^ where the Bazant-Storey-Kornyshev theory^22^ was incorporated with the Langevin fluctuating dipole model for “free” water molecules, with most of the water assumed to be rigidly bound to Li cations. This theory was able to rationalize the overall changes in the composition of the EDL; but, it contains no explicit information on the ionic associations, and assumes that the water bound to each of Li^+^ had infinitely strong associations. Later, McEldrew et al.^23^ developed a theory for thermo-reversible aggregation and gelation in WiSEs,^12^ among other electrolytes,^24–26^ which allowed the cluster distributions and percolating ionic networks to be understood within the framework of Flory, Stockmayers and Tanaka’s famous polymer work. More recently, Goodwin et al.^27,28^ and Markiewitz et al.^29^ extended this formalism to tackle the EDL, but the theory has yet to be rigorously tested against MD simulations and experiments for real electrolytes of interest in the battery and supercapacitor community.

In this paper, we develop a theory for the EDL of WiSEs based on previous work,^12,23,27,29^ where thermoreversible associations are treated consistently, and test it against MD simulations and experiments. This comparison of theory, MD simulations, and experiments allow us to analyze the role these associations have in the structure and properties of WiSEs in the EDL. We found three main conclusions from this investigation. First, we found that at negative voltages, in both theory and MD simulations, electric field-induced associations are present at small negative electrostatic potentials, i.e. over a range of potentials the WiSE *becomes more associated than in bulk*. Moreover, we found the hydrated Li^+^ becomes increasingly dominant with larger negative potentials. At positive voltages, it appears the clusters are strictly diminished when compared to the bulk. The distinct difference in the EDL structure and behavior at negative and positive voltages can be seen through our theory and is presented schematically in Figure 1. Second, we found the theory was able to reproduce trends observed in MD simulations as well as aggregation length scales inferred from AFM measurements. Third, we found that the trends in the theory’s predicted differential capacitance were promising when compared against experimental measurements. Overall, we found that the simple theory presented here can capture how associations change *within* the EDL, which has never been quantified with any theory.

**Figure 1:**
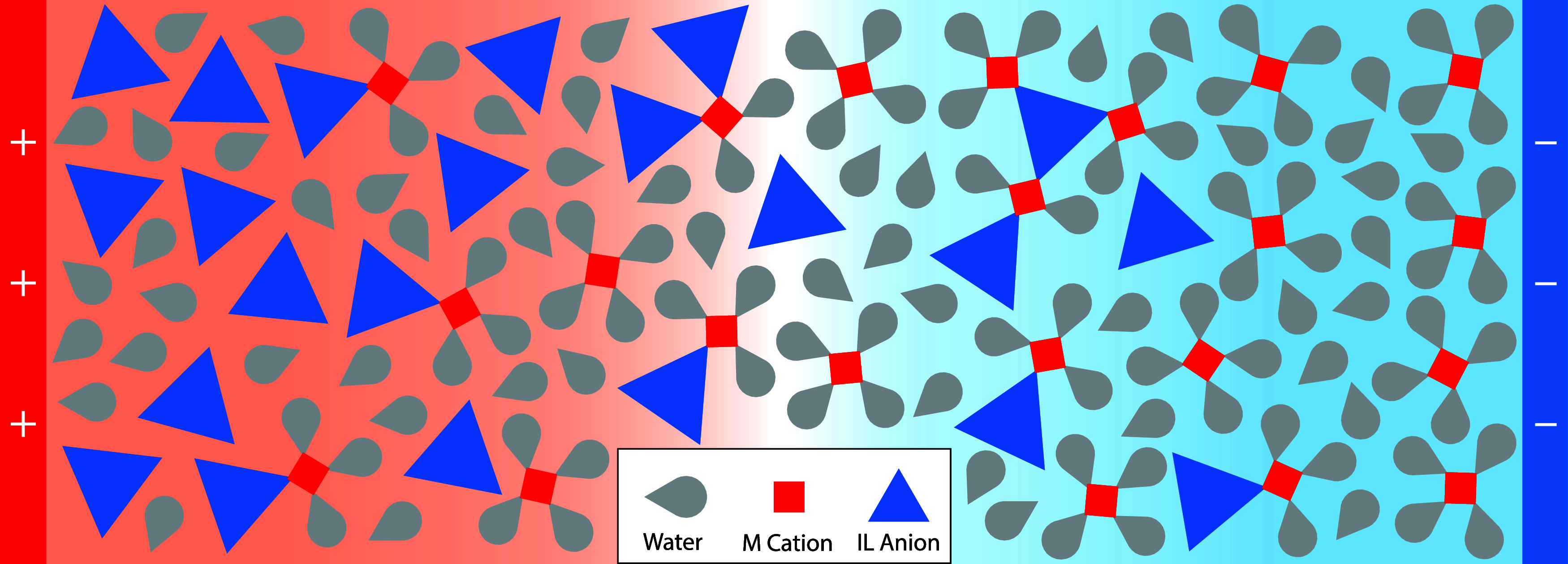
Schematic of the modulation of aggregation occurring in the EDL of WiSE near positively (left) and negatively (right) charged electrodes. Here, the alkali metal cations can form up to 4 associations, the IL anions can form up to 3 associations, and water can form up to 1 association. Ion associations are shown by touching vertices.

## Theory

Here, we consider our system to be an incompressible lattice gas model^23^ composed of alkali metal cations (+), ionic liquid anions (−), and water (0), where we define the size of a lattice site by the volume of a water molecule (*v*_0_), with the volume ratios of all species, ξ_*j*_ = *v*_*j*_/*v*_0_. From these ratios, we can define the dimensionless concentration of a species as *c*_*j*_ = ϕ_*j*_/ξ_*j*_, where (ϕ_*j*_) is the volume fraction of each species and *j* is + ,–,0.

Similar to previous works,^12,26^ we consider the formation of associations between the cations and anions, as well as between cations and water. The cations can form a maximum of *f*_+_ associations, anions can form a maximum of *f*_–_ associations, and water can form a maximum of 1 association; these maximum associations are defined as the functionality of each species. The functionality of a species can be obtained from the maximum coordination numbers in the first solvation shell from MD simulations; and therefore, this is not a free parameter of our theory. When the functionality of the associating species is greater than 1 they can form a set of polydisperse clusters, which can be classified by the rank *lms* of the cluster. This rank specifies the number of cations *l*, anions *m*, and water *s* that comprise the cluster, with the dimensionless concentration of the rank *lms* cluster being *c*_*lms*_. We assume the clusters only form Cayley-tree-like structures, i.e., no loops are present in a cluster.^23^ This Cayley tree assumption for the clusters is necessary to keep this theory analytically tractable and physically intuitive.^23^ This approximation is known to breakdown for some electrolytes,^12^ but it was shown to work well for WiSEs.^25^

When the functionalities of cations and anions are equal to or greater than 2, then a percolating ionic network can form.^23^ This transition is referred to as gelation and is a second-order phase transition. In this gel regime, we employ Flory’s postgel convention to determine the volume fraction of each species in the sol (ϕ_*j*_^*sol*^ this phase contains both the clusters and the free species) and in the gel phase (ϕ_*j*_^*gel*^ this phase contains only the percolating network), where ϕ_*j*_ = ϕ_*j*_^*sol*^ + ϕ_*j*_^*gel*^ and *j* is +, −, 0. The total dimensionless concentration of each species is given by summing over all possible clusters, for example *c*_+_ = ∑_*lms*_ *lc*_*lms*_ + *c*_+_^*gel*^.

The free energy functional ( F ) is proposed to take the following form

$$\begin{array}{rl} \beta F= & \int_{V}dr\left\{-\beta \frac{\epsilon_{0}\epsilon_{r}}{2}(\nabla \Phi {)}^{2}+\beta \rho_{e}\Phi -\frac{c_{001}}{v_{0}}\ln\left(\frac{\sinh(\beta P|\nabla \Phi |)}{\beta P|\nabla \Phi |}\right)\right\}\\ & +\frac{1}{v_{0}}\int_{V}dr\left\{\sum_{lms}(c_{lms}\ln\;\phi_{lms}+\beta c_{lms}\Delta_{lms})+\beta \Delta_{+}^{gel}c_{+}^{gel}+\beta \Delta_{-}^{gel}c_{-}^{gel}+\beta \Delta_{0}^{gel}c_{0}^{gel}\right\}\\ & +\int_{V}dr\left\{\Lambda \left(1-\sum_{lms}(\xi_{+}l+\xi_{-}m+s)c_{lms}-\xi_{+}c_{+}^{gel}-\xi_{-}c_{-}^{gel}-c_{0}^{gel}\right)\right\} \end{array}$$ 1

Here the following variables, electrostatic potential, Φ­(**r**), charge density, ρ_*e*_(**r**), volume fractions/dimensionless concentrations, ϕ­(**r**)/*c*(**r**), and the Lagrange multiplier, Λ­(**r**), all vary in space away from the interface and are integrated over the entire electrolyte domain. The first three terms represent the electrostatic contribution to the free energy: the first subtracts the self-energy of the electrostatic field, the second is the self-interaction energy of the charge density interacting with the mean-field electrostatic potential, and the third is the energy from the fluctuating Langevin dipoles (free water) interacting with the electric field (−∇Φ). The first two terms come from a Legendre transform to enforce Poisson’s equation while taking the variation with Φ.^22,30,31^ The third term comes from the classical theory of random walks with drift, applied to the dipole alignment to the electric field initially by Langevin. This mean-field refinement has been implemented in prior double layer theories^32–34^ and in modeling WiSEs.^11^ The bound water molecules are assumed to not act as fluctuating dipoles. Here ϵ_0_ and ϵ_*r*_, respectively, represent the permittivity of free space and the relative dielectric constant, Φ is the electrostatic potential, ρ_*e*_ is the charge density, given by $\rho_{e}=\frac{e}{v_{0}}(c_{+}-c_{-})$ , with *e* denoting the elemental charge, and *P* is the dipole moment of the free water. The fourth term is the ideal entropy of mixing from the clusters of rank *lms*. The fifth term is the free energy for forming clusters, where Δ_*lms*_ is the free energy of forming the clusters of rank *lms*; this variable is discussed in detail later. The sixth, seventh, and eighth terms represent the free energy of species associating with the gel, Δ_*j*_^*gel*^, which is a function of ϕ_±_ for thermodynamic consistency. The final term is the Lagrange multiplier, which is used to enforce the incompressibility; similar to previous works, this requires one to solve for Λ­(**r**).^29,33^

We can consider the free energy of formation of a *lms* ranked cluster to consist of three contributions,

$$\Delta_{lms}=\Delta_{lms}^{comb}+\Delta_{lms}^{bind}$$ 2

where Δ_*lms*_^*comb*^ is the combinatorial entropy and Δ_*lms*_^*bind*^ is the binding free energy.

The combinatorial entropy comes from the number of ways the ions and water molecules can be arranged in each cluster; in the context of polymers, this was first derived by Stockmayer^23^ for the combinatorial entropy for Cayley tree associations and can be extended to the case of WiSEs,^12^

$$\Delta_{lms}^{comb}=k_{B}T\ln\{f_{+}^{l}f_{-}^{m}W_{lms}\}$$ 3

where

$$W_{lms}=\frac{(f_{+}l-l)!(f_{-}m-m)!}{l!m!s!(f_{+}l-l-m-s+1)!(f_{-}m-m-l+1)!}$$ 4

The binding free energy for an *lms* cluster with *l* > 0 is simply given by,

$$\Delta_{lms}^{bind}=(l+m-1)\Delta f_{+-}+s\Delta f_{+0}$$ 5

where Δ*f*_+*i*_ = Δ*f*_*i*+_ is the free energy of an association between a cation and the *i*th species (anions or water). In the case where *l* = 0, the binding free energy is zero. Note previously, the binding free energy was split up into two terms, the binding energy and the conformational entropy.^23,24,26^

We can calculate the chemical potential of the clusters in the bulk and the EDL, where an overbar will indicate that the variable is the EDL version and Φ is nonzero,

$$\begin{array}{rl} \beta {\overset{®}{\mu }}_{lms}= & (l-m)\beta e\Phi -\ln(\frac{\mathrm{Sinh}(\beta P|\nabla \Phi |)}{\beta P|\nabla \Phi |})\delta_{l,0}\delta_{m,0}\delta_{s,1}+1+\ln({\overset{®}{\phi }}_{lms})+\beta \Delta_{lms}\\ & -(\xi_{+}l+\xi_{-}m+s)\Lambda +(\xi_{+}l+\xi_{-}m+s)\beta {\mathrm{d̅}}^{\prime } \end{array}$$ 6

where *d̅* *′* = *c̅*_+_^*gel*^∂Δ̅_+_^*gel*^ + *c̅*_–_^*gel*^∂Δ̅_–_^*gel*^ + *c̅*_0_^*gel*^∂Δ̅_0_^*gel*^, with the derivative being with respect to ϕ̅_*lms*_.

In the bulk with zero electrostatic potential and field, by asserting the clusters are in equilibrium with the bare species, it follows that,

$$l\mu_{100}+m\mu_{010}+s\mu_{001}=\mu_{lms}$$ 7

From this equilibrium, we can predict the cluster distribution in the bulk with the bare species,

$$c_{lms}=\frac{W_{lms}}{\lambda_{+-}}{\left(\frac{f_{+}\phi_{100}\lambda_{+-}}{\xi_{+}}\right)}^{l}{\left(\frac{f_{-}\phi_{010}\lambda_{+-}}{\xi_{-}}\right)}^{m}(\phi_{001}\lambda_{+0}{)}^{s}$$ 8

where λ_+–_ is the cation–anion association constant and λ_+0_ is the cation-water association constant are given respectively by,

$$\lambda_{+-}=\exp\{-\beta \Delta f_{+-}\}$$ 9

$$\lambda_{+0}=\exp\{-\beta \Delta f_{+0}\}$$ 10

### EDL Equilibrium

By establishing the equilibrium between the free species and the clusters *within* the EDL it follows,

$$l{\overset{®}{\mu }}_{100}+m{\overset{®}{\mu }}_{010}+s{\overset{®}{\mu }}_{001}={\overset{®}{\mu }}_{lms}$$ 11

We obtain an analogous solution to the bulk’s for the EDL cluster distribution given the volume fractions of the bare species in the EDL,

$${\overset{®}{c}}_{lms}=\frac{W_{lms}}{\lambda_{+-}}{\left(\frac{f_{+}{\overset{®}{\phi }}_{100}\lambda_{+-}}{\xi_{+}}\right)}^{l}{\left(\frac{f_{-}{\overset{®}{\phi }}_{010}\lambda_{+-}}{\xi_{-}}\right)}^{m}({\overset{®}{\phi }}_{001}{\overset{®}{\lambda }}_{+0}{)}^{s}$$ 12

where

$${\overset{®}{\lambda }}_{+0}=\lambda_{+0}\frac{\beta P|\nabla \Phi |}{\mathrm{Sinh}(\beta P|\nabla \Phi |)}$$ 13

Note that establishing the equilibrium *within* the EDL allows the aggregation to be consistently treated at the interface, in contrast to previous approaches which only considered the equilibrium in the bulk.^35,36^ In the Supporting Information (SI), we verify that λ̅_+0_ varies in the EDL, supporting the assumptions of our theory. The problem then boils down to consistently linking the equilibriums in the bulk and in the EDL. These approximations are expected to still hold under dynamics. For example, when the bulk goes out of equilibrium, but the EDL and the bulk “just outside” the EDL remains in equilibrium from the effective boundary conditions. This result comes from the asymptotic analysis for thin EDLs.^31^

Following the work of Markiewitz and Goodwin et al.,^27,29^ we can connect the bulk and EDL cluster distributions to the Poisson–Boltzmann equation through closure relations. Here, the closure relations are based on the pregel regime; hence we limit the current analysis to this regime, as other terms should be accounted for in the postgel regime.^27^ This connection is achieved by equating the bare species in the bulk to those in the EDL. For the bare cations

$${\overset{®}{\phi }}_{100}=\phi_{100}\exp(-\beta e\Phi +\xi_{+}\Lambda )$$ 14

there are only contributions from the electrostatic potential and excluded volume effects. For the bare anions

$${\overset{®}{\phi }}_{010}=\phi_{010}\exp(\beta e\Phi +\xi_{-}\Lambda )$$ 15

there are only contributions from the electrostatic potential and excluded volume effects. For the free water

$${\overset{®}{\phi }}_{001}=\phi_{001}\frac{\mathrm{Sinh}(\beta P|\nabla \Phi |)}{\beta P|\nabla \Phi |}\exp(\Lambda )$$ 16

there are only contributions from the fluctuating Langevin dipoles and excluded volume effects. It is important to note here that in previous versions,^27,28^ a parameter α^37^ was introduced to account for the short-ranged correlations between ions, beyond the mean-field that is accounted for here. One can simply introduce the α-parameter by replacing Φ with αΦ. For simplicity, α is excluded here for all but the differential capacitance predictions, where we set α to be 0.1, which has proven to be a reasonable value.^38^ For more details on α’s implementation see the SI.^27,28,37^

Lastly, to solve for the volume fraction of bare species, we need to introduce the idea of association probabilities, conservation of associations, and the law of mass action on said associations.^23^ Knowing the volume fraction of bare species ϕ_100_, ϕ_010_, and ϕ_001_ a priori is uncommon, requiring MD simulations.^12^ This motivates the theory to be designed around the volume fraction of each species that comprise the solution, ϕ_*i*_. One can obtain the desired volume fraction of bare species by introducing the association probabilities *p*_*ij*_, where *i* is the species of interest and *j* is the species that it can form associations with, for this paper they are (*p*_+–_,*p*_+0_,*p*_–+_,*p*_0+_) as it has been shown that associations between water and anions are negligible.^11,12^ Similar to previous works,^12,23,25–27,29^ we can use these probabilities and the functionality of the species to determine the bare species volume fractions, ϕ_100_ = ϕ_+_(1 – *p*_+–_– *p*_+0_)^*f*_+_^, ϕ_010_ = ϕ_–_(1 – *p*_–+_)^*f*_–_^, and ϕ_001_ = ϕ_0_(1 – *p*_0+_).

In order to solve for the bare species, we need four additional equations to determine the association probabilities. These equations are obtained via the conservation of associations and using the law of mass action on the open and occupied association sites.^12,23^ The conservation of cation–anion association produces,

$$\psi_{+}p_{+-}=\psi_{-}p_{-+}=\zeta$$ 17

where ψ_+_ = *f*_+_ϕ_+_/ξ_+_ and ψ_–_ = *f*_–_ϕ_–_/ξ_–_ correspond to the number of cation and anion association sites per lattice site and ζ represents the number of cation–anion associations per lattice site.^12,23^ The conservation of cation-water association provides,

$$\psi_{+}p_{+0}=\phi_{0}p_{0+}=\Gamma$$ 18

where Γ represents the number of cation-water associations per lattice site.^12,23^ Using the law of mass action on the open and occupied association sites for cation–anion associations produces,

$$\lambda_{+-}\zeta =\frac{p_{+-}p_{-+}}{\left(1-p_{+-}-p_{+0})(1-p_{-+}\right)}$$ 19

For cation–anion associations it produces,

$$\lambda_{+0}\Gamma =\frac{p_{+0}p_{0+}}{\left(1-p_{+-}-p_{+0})(1-p_{0+}\right)}$$ 20

where λ_+–_ and λ_+0_ are the association constants for cation–anion associations and cation-water associations, respectively, as they are determined by the equilibrium between the open and occupied association sites. These bulk parameters can be extracted from bulk MD simulations, and thus they are not free parameters. Their EDL counterparts are determined by their bulk value and state variables of the EDL, i.e., they are also not free parameters. An analogous version of these association probability equations uses their EDL quantities and are assumed to hold and smoothly vary in space across the EDL.

### Sticky-Cation Approximation

The complexity of this model can be reduced by utilizing the so-called “sticky-cation” approximation, first introduced in ref 12. where it was asserted and shown that in lithium-based WiSEs the cation associations are sufficiently strong as to fully populate its first solvation shell, i.e., on the time scales of interest the lithium ions always have their max amount of associations. Physically, one can motivate this assumption for Li^+^ as it is the smallest ion present producing the largest local electric field. Thus, Li^+^ is expected to have the strongest and most persistent associations with species in its solvation shell. Therefore, it follows that its first shell will be completely filled. This treatment relaxes the previous treatment of the first solvation shell of Li^+^ for WiSE in the EDL seen in McEldrew et al.,^11^ where four waters and Li^+^ were assumed to form a single effective cation. Our current treatment allows the cation to bind to water, but also the anions to form aggregates. This assumption fundamentally reduces down to the constraint *p*_+–_ + *p*_+0_ = 1. Additionally, this leads to singularities in the law of mass action equations, eqs 19 and 20; this can be overcome by taking the ratio of these equations,

$$\lambda =\frac{\lambda_{+-}}{\lambda_{+0}}=\frac{p_{-+}(1-p_{0+})}{p_{0+}(1-p_{-+})}$$ 21

where λ is the cation association constant ratio. Furthermore, as this assumption is incompatible with having bare cations, eq 14 must be replaced; this is achieved by considering the equilibrium between the fully hydrated cation in the bulk and the EDL,

$${\overset{®}{\phi }}_{10f_{+}}=\phi_{10f_{+}}\exp(-\beta e\Phi +(\xi_{+}+f_{+})\Lambda )$$ 22

where ϕ_10*f*_+__ = (1 + *f*_+_/ξ_+_)­ϕ_+_(1 – *p*_+–_)^*f*_+_^. Additionally, as the sticky cation approximation requires that *s* = *f*_+_ *l* – *l* – *m* + 1, the sticky-cation cluster distribution simplifies to

$$c_{lm}=\frac{\phi_{0}\alpha_{0}W_{lm}}{\lambda }{\left(\lambda \frac{\psi_{+}\alpha_{+-}}{\phi_{0}\alpha_{0}}\right)}^{l}{\left(\lambda \frac{\psi_{-}\alpha_{-}}{\phi_{0}\alpha_{0}}\right)}^{m}$$ 23

where

$$W_{lm}=\frac{(f_{+}l-l)!(f_{-}m-m)!}{l!m!(f_{+}l-l-m+1)!(f_{-}m-l-m+1)!}$$ 24

Here, α_0_ = 1 – *p*_0+_, α_+–_ = (1 – *p*_+–_)^*f*_+_^, and α_–_ = (1 – *p*_–+_)^*f*_–_^ are the fraction of free water molecules, fully hydrated cations, and free anions, respectively. Additionally, an analogous set of equations exists for the EDL. Lastly by using *p*_+–_ + *p*_+0_ = 1 with eqs 17, 18, and 21, one can explicitly solve for the association probabilities in terms of λ, ψ_+_, ψ_–_, and ϕ_0_, the equations for which are shown in the SI.

### Modified Poisson–Boltzmann

To predict WiSEs behavior in the EDL, we can derive our modified Poisson–Boltzmann (PB) equation by taking the functional derivative of the free energy with respect to the electrostatic potential,

$$\nabla \cdot (\epsilon \nabla \Phi )=-\rho_{e}=-\frac{e}{v_{0}}({\overset{®}{c}}_{+}-{\overset{®}{c}}_{-})$$ 25

where

$$\epsilon =\epsilon_{0}\epsilon_{r}+P\frac{{\overset{®}{c}}_{001}}{v_{0}}\frac{L(\beta P|\nabla \Phi |)}{\left|\nabla \Phi \right|}$$ 26

and *L*(*x*) = coth­(*x*) – 1/*x* is the Langevin function. Here, we can define the inverse Debye length, $\kappa =\sqrt{e^{2}\beta (c_{+}+c_{-})/v_{0}\epsilon_{0}\epsilon_{r}}$ , which will be used to express the distances from the electrode as dimensionless values. Simple modified PB approaches model the correlations of point-like charges interacting through a uniform dielectric media, which is only technically valid in the dilute electrolyte limit.^39^ While fluctuations from the dipole moment of free water molecules are accounted for here, nonlocal effects still exist that are not captured in this simple modified PB equation. Other modified PB equations have been developed to correct for the effects of finite ion sizes, Coulomb correlations, and nonlocal dielectric responses.^22,31,33,34,40,41^ Similar to the theory of SiILs in the EDL,^29^ our simple model may indirectly capture these corrections through the short-range associations that promote the formation of ionic clusters with solvent decorations. These clusters have a “spin-glass” ordering, i.e. ions favor oppositely charged neighbors.^42^ The procedure implemented to solve our system of equations coupled to the modified PB equations is discussed in detail in the SI.

### EDL Prediction

Beyond the spatial profiles, cluster distributions, and how aggregated the WiSE is in the EDL, one can extract other informative quantities such as the screening length and the length scale of the aggregates. The screening length can be extracted by fitting an exponential function to the electrostatic potential at small potentials for various molalities. Obtaining the screening length allows one to test the consistency of the model’s electrostatic predictions. The length scale of the aggregates ( $l_{A}$ ) can be obtained in the pregel regime by solving,

$$l_{A}^{3}=v_{0}\sum_{lms}(\xi_{+}l+\xi_{-}m+s{)}^{2}{\overset{®}{c}}_{lms}$$ 27

The $l_{A}$ may be used to understand more thoroughly the structuring near charged interfaces, allowing for qualitative comparison against experimental results.^19^

A valuable aspect of mean-field models is their tendency to provide reasonable predictions for integrated quantities such as the excess surface concentrations,^11,43,44^ the interfacial concentration of water,^11^ and the differential capacitance. The excess surface concentrations provide an integrated perspective on how the composition of the electrolyte is affected by being in the presence of a charged interface,

$$\Gamma_{i}(q_{s})=\frac{1}{v_{0}}\int_{0}^{\infty }({\overset{®}{c}}_{i}(x,q_{s})-c_{i}^{\mathrm{bulk}})dx$$ 28

where *q_s_* is the surface charge density at the interface, *c*_*i*_^bulk^ is the dimensionless concentration of the bulk electrolyte solution, and where *x* is the dimensional distance from the interface. We obtain these predictions by directly integrating the numerical solutions from the modified PB equations and the MD simulations.

The interfacial concentration of water provides deeper insight into the average composition of the EDL and the amount of free interfacial water present. This measurement can provide insight into how accessible water is to undergo reactions at the interface.^11^ This quantity can be obtained from both theory and MD simulations by integrating a distance $l_{w}$ from the charged interface and normalizing their bulk value over this distance,

$${\tilde{\rho }}_{w,n}^{ads}(q_{s})=\frac{\int_{0}^{l_{w}}{\overset{®}{c}}_{n}(x,q_{s})dx}{l_{w}c_{0}^{\mathrm{bulk}}}$$ 29

Here $l_{w}$ was chosen to be 5 Å, and n indicates the form of water, i.e., total is *c̅*_0_, free is *c̅*_001_, and bound is *c̅*_0_ – *c̅*_001_.

The differential capacitance, *C*, also known as the double layer capacitance, can be calculated as,

$$C=\frac{dq_{s}}{d\Phi_{s}}$$ 30

Here, Φ_*s*_ is the electrostatic potential at the charged interface, equivalent to the potential drop across the EDL.

## Results

Here, we will mainly discuss the EDL properties of 15m water-in-LiTFSI (12m water-in-LiTFSI is shown in the SI), describing and comparing the theory’s predictions under the sticky cation approximation (nonstick case shown in SI) against the predictions from MD simulations and later experimental data. The theory and its predictions discussed here build strongly on previous studies of WiSEs in bulk, such as ref 12 and concentrated electrolytes in the EDL, see refs 27 and 29. In the SI, we have included the MD simulation methodology, experimental protocols, sticky cation approximation, numerical maps of how the WiSEs properties change as a function of the electric potential and the magnitude of the electric field, and the results under different conditions.

### Bulk WiSE

Before investigating the EDL properties of WiSE, it is first prudent to review the aggregation behavior in the bulk. In order to model the bulk, one must first find the salt’s functionalities, which can be determined from the coordination number distributions. Here we found that in our simulations of pregel water-in-LiTFSI, Li^+^ has an average coordination number greater than 4 (approximately 4.5). This is in agreement with experiments, where they find the first hydration shell of Li^+^ contains 4–5 water molecules as determined by neutron diffraction experiments.^45^ This finding is also similar to previous work,^12^ in which they set the functionality to be 4 and created the sticky cation approximation, i.e., the cation is always fully associated, as it better modeled the simulation data. As our finding suggested a Li^+^ functionality of either 4 or 5, we tested and verified the adequacy of using the sticky-cation approximation in the EDL with functionality of 4, and nonsticky case with functionality 5. This was also done with 12m water-in-LiTFSI and is discussed in detail in the SI. Based on the behaviors of TFSI^–^ associations, its maximum cation coordination is 3.^12^ Therefore, we concluded Li^+^ functionality to be 4 under the sticky-cation approximation and the functionality of TFSI^–^ to be 3, both values agree with the previous study.^12^

The association probabilities can also be extracted from the simulations, which are discussed in detail in the SI. From the bulk association probabilities and mass action laws, we were able to extract the cation association constant ratio, λ, using eq 21 to find it is 0.231 for 15m water-in-LiTFSI. From the molality, we can obtain the volume fraction of each species in the WiSE. Using the volume fractions, functionalities, and λ one can predict the bulk association probabilities from our theory. Lastly, we can also predict the gel-point, which is given by 1 – (*f*_+_ – 1)­(*f*_–_– 1)*p*_+–_^*^ *p*_–+_^*^ = 0.^23^ This criterion comes from calculating the critical probabilities for which the ionic backbone of these clusters can become infinitely large. The proximity of *p*_+–_ *p*_–+_ to *p*_+–_^*^ *p*_–+_^*^ provides insight into how large the aggregates are and how close the solution is to gelation. For 15m LiTFSI, we find both from the sticky-cation theory and simulations that the WiSE is just under the gelation point.

Since the theory is fully parametrized for bulk 15m water-in-LiTFSI, we can investigate the bulk cluster distribution using eq 23. In Figure 2, we show schematics of some of the most common clusters in the LiTFSI WiSE: cations hydrated with 4 water molecules, free anions, hydrated ion pairs, and sample multi-ion clusters. In Figure 2, the bulk cluster distribution from the sticky-cation theory for 15m water-in-LiTFSI is shown. An informative quantity is *c*_10*f*_+__/*c*_010_, which tells us how positively or negatively biased the “free” species are. Additionally from this quantity, one can deduce the sign of the net charge bias of the clusters. Moreover, it can be shown to depend only on the ratio of anion to cation functionality (*f*_–_/*f*_+_), for the derivation see SI. Here one can note that the distribution beyond the hydrated Lithium and the free TFSI^–^ is marginally biased toward net negative clusters, which occurs because the functionality of cations is larger than anions. This preference for slightly negative ionic aggregates is balanced by the excess amount of hydrated cations compared to free anions, giving overall electroneutrality in the bulk. Intuitively, the negative cluster bias can be noted that there are more “free” cations than “free” anions. Since the bulk must satisfy global electroneutrality, i.e., *c*_+_ = *c*_–_, then the difference in the “free” species *c*_10*f*_+__ – *c*_010_ must be compensated in the multi-ion clusters (*c*_10*f*_+__ + *c*_+_^*Agg*^ = *c*_010_ + *c*_–_^*Agg*^). For this reason, *c*_10*f*_+__ – *c*_010_ > 0 ⇒ *c*_–_^*Agg*^ – *c*_+_^*Agg*^ > 0, i.e., the multi-ion clusters must have a net negative bias. An alternative visual representation and explanation of this cluster bias are presented in the SI for both the theory and MD.

**Figure 2:**
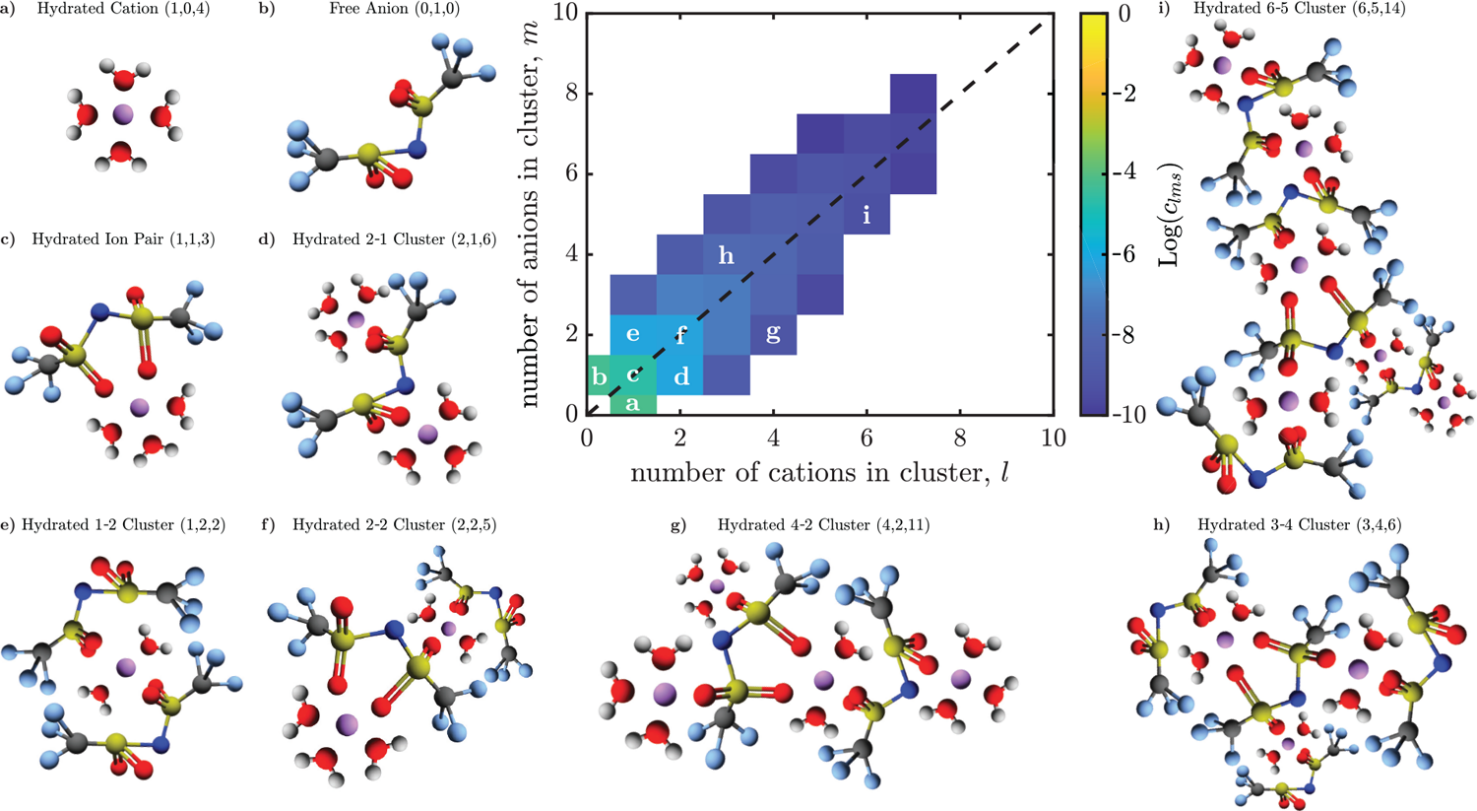
Cluster distribution of bulk 15m water-in-LiTFSI. Here we use *f*_+_ = 4, *f*_–_ = 3, ξ_0_ = 1, ξ_+_ = 0.4, ξ_–_ = 10.8, ϵ_*r*_ = 10.1, λ = 0.231, *P* = 4.995 D, and *v*_0_ = 22.5 Å^3^. The cluster distribution is surrounded by a sample of schematics of the common clusters in bulk 15m water-in-LiTFSI visualized using Avogadro 1.2.0.^46^ These sample clusters are shown in (a–i). (a) Hydrated Cation (1,0,4). (b) Free Anion (0,1,0). (c) Hydrated Ion Pair (1,1,3). (d) Hydrated 2–1 Cluster (2,1,6). (e) Hydrated 1–2 Cluster (1,2,2). (f) Hydrated 2–2 Cluster (2,2,5). (g) Hydrated 4–2 Cluster (4,2,11). (h) Hydrated 3–4 Cluster (3,4,6). (i) Hydrated 6–5 Cluster (6,5,14).

### Anode EDL

In Figure 3, we show the predicted properties of the EDL for negatively charged interfaces from both MD and theory. All plots are shown as a function from the charged interface, in dimensionless units of distance, which is normalized by the inverse Debye length, κ. Here 1/κ = λ_*D*_ ∼ 0.5 Å with ϵ_*r*_ = 10.1 and *P* = 4.995 D, which respectively comes from the relative dielectric constant and the dipole moment of water-in-LiTFSI obtained in previous MD simulations of water-in-LiTFSI solutions.^11^ The gray regions in the simulation plots below indicate the area closest to the interface where one species did not exist in the simulations, determined from the center-of-mass. In this region, extracting the probabilities from MD simulations is not well-defined, and moreover, the theory is expected to break down near this condensed layer. The deviations are expected in the condensed layer, also known as a Helmholtz layer, as a result of the surface, nonlocal, and finite size effects leading to oscillations and species exclusion.

**Figure 3:**
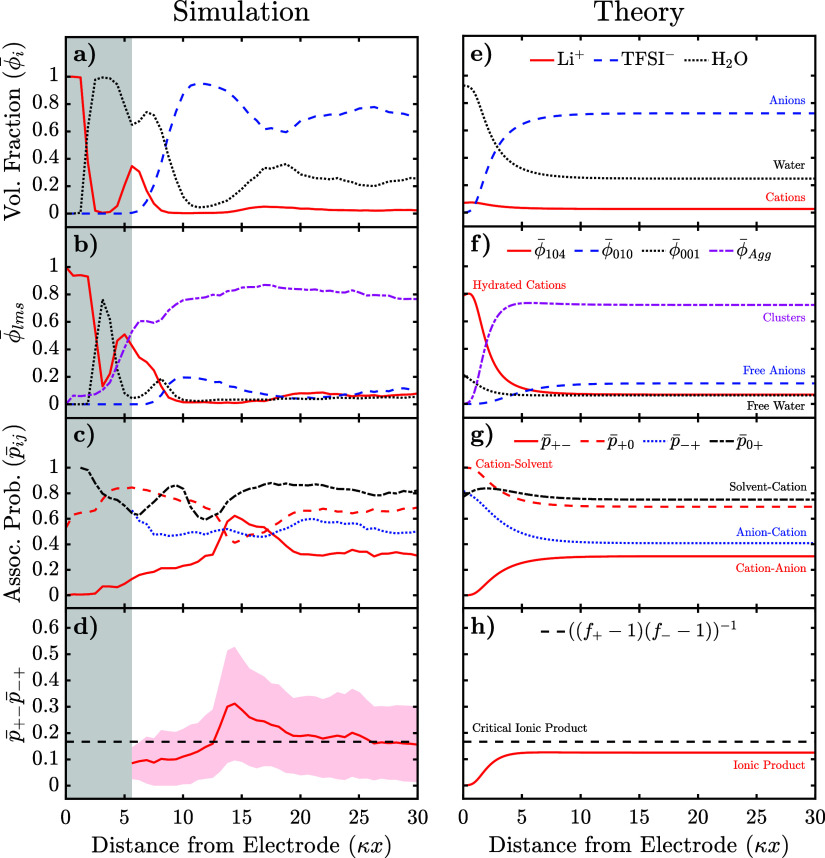
Distributions of properties of 15m WiSEs in the EDL as a function from the interface, in dimensionless units, where κ is the inverse Debye length. (a–d) are the results from MD simulations, and (e–h) are the corresponding predictions from theory. The gray region indicates the minimum distance from the electrode at which a species was never found. (a,e) Total volume fraction of each species. (b,f) Volume fractions of hydrated cations (Simulation ϕ̅_10*x*_ & Theory ϕ̅_104_), free anions, free water, and aggregates. (c,g) Association probabilities. (d,h) Product of the ionic association probabilities, *p̅*_+–_ *p̅*_–+_, where the dashed line indicates the critical line for gelation. Here we use *f*_+_ = 4, *f*_–_ = 3, ξ_0_ = 1, ξ_+_ = 0.4, ξ_–_ = 10.8, ϵ_*r*_ = 10.1, λ = 0.231, *P* = 4.995 D, *v*_0_ = 22.5 Å^3^, and *q*_s_ = −0.2 C/m^2^.

In Figure 3a,e the volume fraction of the Li^+^ (ϕ̅_+_), TFSI^–^ (ϕ̅_–_), and H_2_O (ϕ̅_0_) from, respectively, the simulation and theory are displayed. In Figure 3a, one can identify three distinct regions for the cations, with minimal population in between. The first layer is found at the interface where the cations have saturated, i.e., its volume fraction reaches 1, followed by a depleted region where water dominates. Following this hydration layer, another cation peak is found with water at 6λ_*D*_, followed by a large volume fraction of anions. Lastly, there is a small third layer of cations at 17λ_*D*_, after which the volume fraction of cations fluctuates around the bulk value. In Figure 3a, the water forms two distinct layers, the first being the hydration layer around 3–7λ_*D*_, which follows the saturated layer of cations and is smeared out into the second cation peak before being depleted by the large anion layer. The second water peak is after the anion layer around 19λ_*D*_ and decays into the bulk oscillations of the system. Lastly, the anions are depleted just before the condensed layer; before this, they peak around 11λ_*D*_ and subsequently fluctuate around their bulk value.

These MD results can be compared against the theory in Figure 3e. The theory predicts an increase in the cation volume fraction approaching the interface before negligibly decreasing close to the interface, which agrees with the simulation trends, albeit without the oscillations and surface structuring observed in the simulations. Similarly, the water volume fraction slowly increases until close to the interface where it rapidly increases. This predicted trend by the theory agrees roughly with the simulations, as the oscillations and surface structuring, respectively, leads to fluctuations throughout the EDL and more refined structuring in the condensed layer. Lastly, the anion volume fraction slowly decreases until it rapidly goes to zero closer to the interface. In this case, the theory more accurately captures the trends seen in the simulations; this result follows from the anions not being present in the condensed layer making the diffuse nature of our theory even more apt. However, we still see deviation from the simulations through oscillations, but this is expected from the local approximations used in our theory not capturing the overscreening behavior of the concentrated electrolyte (see Discussion Section). The value of the presented theory is not in predicting the exact distribution of each species in the EDL, but in investigating how the associations change within the EDL, which will now be described.

Considering the volume fraction of specific clusters in the EDL provides deeper insight into the structure of the volume fraction of each species. Observe first the free anion volume fraction for the simulation in Figure 3b, ϕ̅_010_, is found to be significantly smaller than ϕ̅_–_ as much of the anions are in clusters. In Figure 3f, the decaying trend in ϕ̅_010_ toward the electrode’s surface is replicated qualitatively by the theory.

Second, let us consider the free water volume fraction, ϕ̅_001_. From the simulation in Figure 3b, a sharp peak in ϕ̅_001_ is observed in the middle of the condensed layer at 3λ_*D*_. A little outside of the condensed layer, ϕ̅_001_ peaks again at 8λ_*D*_. Following this, ϕ̅_001_ decays and fluctuates around its bulk value. In Figure 3f, the theory predicts ϕ̅_001_ monotonically increases as it approaches the charged interface, albeit the absolute values are quite different. This enrichment of ϕ̅_001_ near the negatively charged interface qualitatively captures the behavior of ϕ̅_001_ observed in the simulation.

Third, one can consider the volume fraction of aggregates containing more than one ion, ϕ̅_*Agg*_. From the simulation in Figure 3b, one can observe that this profile slightly increases entering the EDL followed by a gradual decay before a rapid decay to a near zero value through the condensed layer. In Figure 3f, this qualitative trend is found in the theory with the local maximum in aggregation being notable in this curve around 6λ_*D*_. This local maximum emerges from both the cations and anions existing in similar and appreciable amounts, instead of there being one dominant ion present, which maximizes the aggregation emerging. This kind of phenomenon has been predicted to occur in other concentrated electrolytes such as Salt-in-Ionic-Liquids.^29^

Lastly, the volume fraction of various degrees of hydration (*x*) cations, ϕ̅_10*x*_, from the simulation is seen in Figure 3b. At the surface, ϕ̅_10*x*_ saturates, excluding free water from the interface. Following this layer, ϕ̅_10*x*_ decays slightly before rapidly increasing, producing a second ϕ̅_10*x*_ peak in the condensed layer. This second ϕ̅_10*x*_ peak occurs at 5λ_*D*_, which is around the same location as the second ϕ̅_+_ peak. After this peak, ϕ̅_10*x*_ is depleted until further away from the interface where ϕ̅_10*x*_ obtains its third and final peak in the EDL at 21λ_*D*_ after which it fluctuates around its bulk value. All the peaks in ϕ̅_10*x*_ correspond to the peaks in ϕ̅_+_ in Figure 3a, but the peaks in ϕ̅_10*x*_ are broader and dissipate slower. For the theory, as we are considering the sticky-cation approximation, the only hydrated cation cluster is x = f_+_, so ϕ̅_10*x*_=ϕ̅_104_. In Figure 3f, the theory predicts that the volume fraction of hydrated cations increases when approaching the negatively charged interface, with the fastest increase within 5λ_*D*_ of the interface. Close to the interface, a local maximum in ϕ̅_104_ is predicted. The theory is able to capture qualitatively the increase of ϕ̅_104_ in the diffused EDL and dominate presence of ϕ̅_104_ close to the interface.

At this time, let us consider how the negatively charged electrode impacts the association probabilities, which no prior theory of WiSEs in EDL has been able to predict. From the simulation in Figure 3c, one can note that the association probability of cations being bound to an anion, *p̅*_+–_, increases initially after entering the EDL, before gradually decaying to zero as the interface is approached. The association probability of cations being bound to a water, *p̅*_+0_ initially decreases before increasing around 15λ_*D*_ from the interface, which is the same turning point as seen for *p̅*_+–_. Upon entering the condensed layer, *p̅*_+0_ decays; this behavior appears to result from the excess amount of Li^+^’s in the condensed layer without enough water to fully fill their first solvation shell. Note in this analysis, associations of species with the wall are not considered. From the theory in Figure 3g, *p̅*_+–_ gradually decays before quickly decaying near the interface, and *p̅*_+0_ gradually increases before quickly increasing near the interface. The general trends of *p̅*_+–_ and *p̅*_+0_ in the EDL seen in the simulation can be captured by the theory.

Next, one can consider in Figure 3c that the simulation of the anion–cation association probability *p̅*_–+_, appears to slightly increase while undergoing broad fluctuations when approaching the negatively charged interface and is ill-defined in the condensed layer. While the direction of *p̅*_–+_ in the simulation is captured in the theory seen in Figure 3g, the magnitude of the change in *p̅*_–+_ is larger in the theory.

Lastly, in Figure 3c, the water-cation association probability from the simulation, *p̅*_0+_ is shown. Here, *p̅*_0+_ slightly increases until ∼17λ_*D*_. After which *p̅*_0+_ strongly oscillates with a negative trend until the condensed layer. In the condensed layer, *p̅*_0+_ strongly increases toward 1. The theory can be seen in Figure 3g where *p̅*_0+_ slowly increases until close to the interface, where it then decreases slightly. Overall the theory can capture the rough behavior of *p̅*_0+_ seen in the simulation.

Finally, one can consider how ionic associations are impacted by the EDL through the product of the cation–anion and anion–cation association probabilities, *p̅*_+–_ *p̅*_–+_, another observable which prior theories have not be capable of providing insight on for WiSE in the EDL. From the simulation in Figure 3d, one can observe an initial increase in *p̅*_+–_ *p̅*_–+_ where it even crosses the critical value for gelation and reaches a maximum around 14.5λ_*D*_. Following this maximum, the electrostatic potential and electric field strength achieve sufficiently strong values to melt the induced gel. Comparing this result against the theory in Figure 3h, *p̅*_+–_ *p̅*_–+_ increases very slightly, peaking around 7λ_*D*_, before rapidly decaying to zero, shown in greater resolution in the SI. Once again, the theory appears to predict qualitative trends in the WiSE solution in the EDL, but the overall changes are smoother. Note that the association probabilities can also be combined to compute how the association constants vary in the EDL, as we show in the SI, where good agreement with the theory is found.

Overall, by considering the simulation results and theory’s predictions near a negatively charged interface in Figure 3, one can consider the EDL to be structured by the following regions: (1) a condensed cation layer with some bound water molecules, (2) a hydration layer filled with free water and some hydrated cations, (3) a cation rich layer with fully hydrated cations, aggregates, and trace amounts of free water, (4) a small peak in aggregation, and (5) bulk region. Additionally the qualitative trends in the diffuse sections of the EDL produced by the theory appear to agree with the MD simulation results sufficiently to provide previously inaccessible insights into the association environments in the EDL, both in terms of ionic associations and solvent associations.

### Cathode EDL

A similar analysis can be conducted for a positively charged electrode in Figure 4. The total volume fractions obtained with MD simulation can be seen in Figure 4a. In Figure 4a, as one approaches the interface, the volume fraction of Li^+^, ϕ̅_+_, fluctuates around its bulk value before increasing slightly for a short region and then dropping to zero, which is consistent with overscreening. While the overarching trend in ϕ̅_+_ depleting near the interface is captured by the theory, as seen in Figure 4e, the higher order correlation effects such as overscreening are not captured, which will be discussed in greater detail later. Analogous to the Anode EDL, there exists a condensed layer where significant deviations are expected and can be improved in future theory extensions as discussed previously in the Anode EDL subsection.

**Figure 4:**
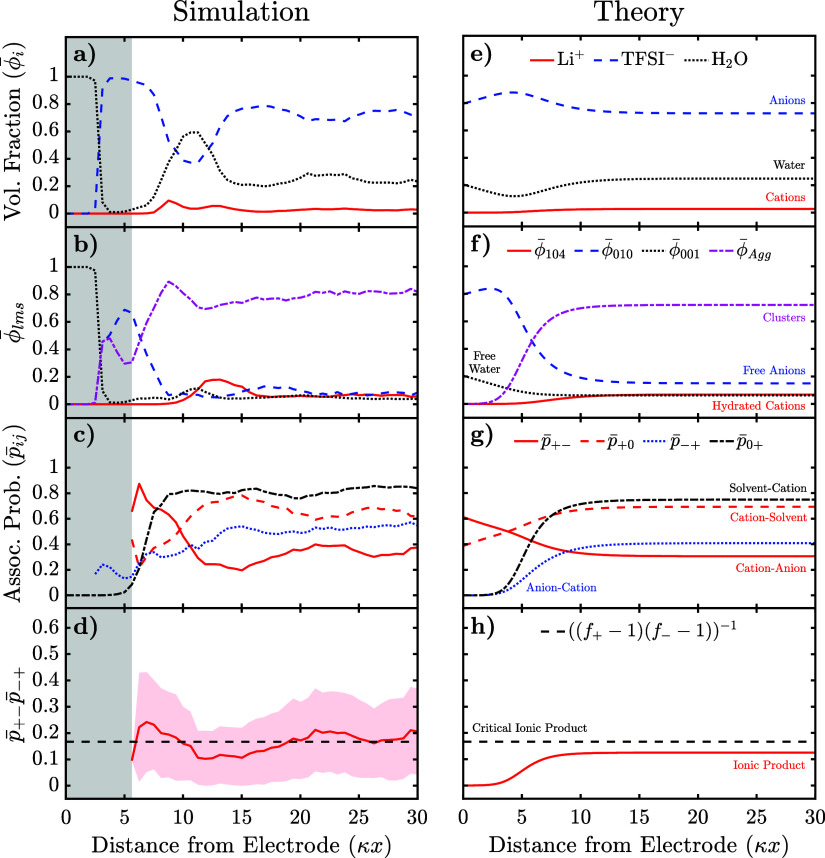
Distributions of properties of 15m WiSEs in the EDL as a function from the interface, in dimensionless units, where κ is the inverse Debye length. (a–d) are the results from MD simulations, and (e–h) are the corresponding predictions from theory. The gray region indicates the minimum distance from the electrode at which a species was never found. (a,e) Total volume fraction of each species. (b,f) Volume fractions of hydrated cations (Simulation ϕ̅_10*x*_ & Theory ϕ̅_104_), free anions, free water, and aggregates. (c,g) Association probabilities. (d,h) Product of the ionic association probabilities, *p̅*_+–_ *p̅*_–+_, where the dashed line indicates the critical line for gelation. Here we use *f*_+_ = 4, *f*_–_ = 3, ξ_0_ = 1, ξ_+_ = 0.4, ξ_–_ = 10.8, ϵ_*r*_ = 10.1, λ = 0.231, *P* = 4.995 D, *v*_0_ = 22.5 Å^3^, and *q*_s_ = 0.2 C/m^2^.

Considering the simulation prediction for the volume fraction of H_2_O, ϕ̅_0_, in Figure 4a one notes the two peaks. The first being the hydration layer at the interface followed by a depletion region where the first anion layer can be found. This layer is followed by the second peak at 11λ_*D*_ in ϕ̅_0_ before it drops to its bulk value. The theory predicts an initial decay in ϕ̅_0_ entering the EDL before quickly increasing close to the interface. This behavior qualitatively captures the key features of the ϕ̅_0_ curve from the MD simulation.

Now, one can consider the behavior of the volume fraction of TFSI^–^, ϕ̅_–_, in the EDL displayed in Figure 4a. Upon entering the EDL, ϕ̅_–_ retains its bulk value before decaying to about half its bulk value. Following this region, there is an anion layer with little water present around 5λ_*D*_. Entering the condensed layer close to the interface, ϕ̅_–_ goes to zero as the hydration layer excludes the anions from the interface. Considering the theory’s prediction for ϕ̅_–_ in Figure 4e, the anions initially increase their presence in the EDL before gently decaying near the interface.

Next, one can consider the volume fraction of a specific cluster near a positively charged electrode. First, from simulation in Figure 4b, the volume fraction of free anions, ϕ̅_010_, stays near the bulk value in the EDL until reaching the anion layer around 5λ_*D*_ where it then increases significantly, but seemingly a little less than half of these anions remain in a cluster. Following this, ϕ̅_010_ decays quickly to zero in the hydration layer. The theory’s prediction is seen in Figure 4f, with ϕ̅_010_ increasing and reaching a local maximum close to the interface before slowly decaying. Here, the theory appears to capture the general behavior but lacks the ability to account for the finite nature of these clusters, which is expected given the current theory’s point-like treatment of species. These effects, along with specific surface interactions, become increasingly important at closer distances to an interface.

Second by simulation in Figure 4b, one can see that the volume fraction of free water’s, ϕ̅_001_, enhancements in the diffuse part of the EDL are in line with ϕ̅_0_’s but scaled down significantly as it happens that most of the water is bound in the bulk solution. However, close to the interface, a “hydration” layer is filled with free water molecules. Similar to the theory’s prediction for the ϕ̅_010_, its prediction for ϕ̅_001_ is consistent with a qualitative trend from the MD simulation, with the condensed layer producing more complicated effects, such as the “hydration” layer at the interface.

Third, one can consider how EDL influences multi-ion aggregates obtained through simulation in Figure 4b. In Figure 4b one can observe that the volume fraction of aggregates with more than one ion, ϕ̅_*Agg*_, is zero through the “hydration” layer after which it takes on half of its bulk value in the anion layer, before returning to around its bulk value. This result demonstrates how the TFSI^–^ extended nature allows it to form clusters across the EDL. This deviation occurs as the anion can form associations with cations further away from its center-of-mass, which hinders local theories’ ability to capture oscillations as short-ranged ordering is averaged out. As expected in Figure 4f, the theory predicts ϕ̅_*Agg*_ to decay toward the interface monotonically. This decay loosely agrees with the trend seen in the MD simulation, without the strong oscillations.

Lastly, one can consider the simulation’s prediction for the volume fraction of hydrated cations, ϕ̅_10*x*_, in Figure 4b. Approaching the interface, ϕ̅_10*x*_ fluctuates around its bulk value, until it increases and obtains a peak value around 13λ_*D*_ from the interface before rapidly decaying to zero. This peak at 13λ_*D*_ is at a similar location to the second local maximum of ϕ̅_+_ in Figure 4a but has a larger peak amplitude. Considering ϕ̅_+_ in Figure 4a with higher resolution, there are two local maximum’s at 8λ_*D*_ and 13λ_*D*_. The first maximum at 8λ_*D*_ appears to correspond to cations in multi-ion clusters as the peak is missing from the ϕ̅_10*x*_’s profile, but is present in the ϕ̅_*Agg*_ profile. From this information, we can infer that the majority of the cations present in the second peak in ϕ̅_+_ around 13λ_*D*_ exist as hydrated cations. For the theory as we are considering the sticky-cation approximation, the only hydrated cation cluster is x = f_+_, hence ϕ̅_10*x*_=ϕ̅_104_. The theory in Figure 4f predicts that the hydrated cations monotonically decay, which qualitatively captures the diffuse EDL’s global behavior, but misses this local maximum.

Now, let us consider how the association probabilities are influenced near a positively charged electrode. This is another key prediction that could provide insight into the various properties of WiSEs and one which prior theories of WiSEs in the EDL were incapable of predicting. In Figure 4c from the simulations, one can note that the association probability of cations being bound to an anion, *p̅*_+–_, fluctuates around its bulk value until it quickly increases close to the condensed layer. In Figure 4g, the theory predicts that *p̅*_+–_ increases into the EDL in a similar fashion. Comparing *p̅*_+–_ profile from the simulation and the theory displays an adequate match in the qualitative trends. In Figure 4c, the value of the association probability of cations being bound to water, *p̅*_+0_, fluctuates around its bulk value before quickly decreasing near the condensed layer, in a roughly reversed response to the EDL than *p̅*_+–_. In Figure 4g, the theory predicts an equivalent behavior for *p̅*_+0_. Hence, the theory is able to qualitatively capture the trends in *p̅*_+0_ seen in the simulation.

Next as seen in the MD simulation in Figure 4c, the association probability of anions being bound to a cation, *p̅*_–+_, slowly decreases through the EDL. The decay of *p̅*_–+_ near the interface is more gentle in the simulation compared to the theory’s prediction. In Figure 4g, the theory predicts *p̅*_–+_ to behave similarly to the simulation trends with minor decreases in the bulk of the EDL. Lastly through simulation in Figure 4c, the association probability of waters being bound to a cation, *p̅*_0+_, fluctuates around its bulk value for much of EDL before rapidly decaying to zero before and through the condensed layer. From the theory in Figure 4g, *p̅*_0+_ is observed to have a strikingly similar behavior to the simulation prediction with negligible decay in much of the EDL before rapidly decaying close to the interface. Overall, the theory appears to adequately agree with the qualitative trends presented in the MD predictions for the association probabilities in the EDL.

Finally, let us consider how ionic associations are impacted by the EDL through the product of the ionic association probabilities, *p̅*_+–_ *p̅*_–+_, is impacted by the positively charged interface, once again an interesting prediction which previous theories of WiSEs in the EDL are unable to predict. In Figure 4d, *p̅*_+–_ *p̅*_–+_ can be seen fluctuating, in a seemingly decreasing fashion. Here, it appears to generally exist close to the critical threshold. In Figure 4h, *p̅*_+–_ *p̅*_–+_ is predicted to gently decay before rapidly decaying close to the interface. The differences between these curves further support the important role the condensed layer may have on the structure of the EDL itself and how these effects propagate into the bulk.

Both the simulation and theory can give a detailed picture of the EDL structure near a positively charged electrode. Here, we have found it has four distinct regions: (1) a hydration layer of free water molecules, (2) an anion-rich layer filled with free anions and anions associated with cations from further away from the interface, (3) an enriched cluster and hydrated cation layer, and (4) the bulk. Overall, the theory appears to adequately capture the qualitative trends from the MD simulations, while its spatial profiles are compressed at points due to a short screening length.

### Size and Structure of EDL Aggregates

One direct way associations impact the EDL is through the length scale of aggregates, $l_{A}$ , which can be experimentally obtained through SFA and AFM measurements. In Figure 5, $l_{A}$ predicted from theory near a negatively charged electrode is displayed along with its predicted value from MD simulations, and experimentally from force–distance measurements by AFM on mica^19^ in the inset. In Figure 5, the MD simulation predicts that for a negatively charged surface $l_{A}$ fluctuates around its bulk value far from the interface before gradually decaying as it approaches the interface. In the condensed layer, $l_{A}$ rapidly decays, taking on a final value around the length scale of a fully hydrated Li cation. The theory captures the general trend, however, the bulk value deviates from the MD results.

**Figure 5:**
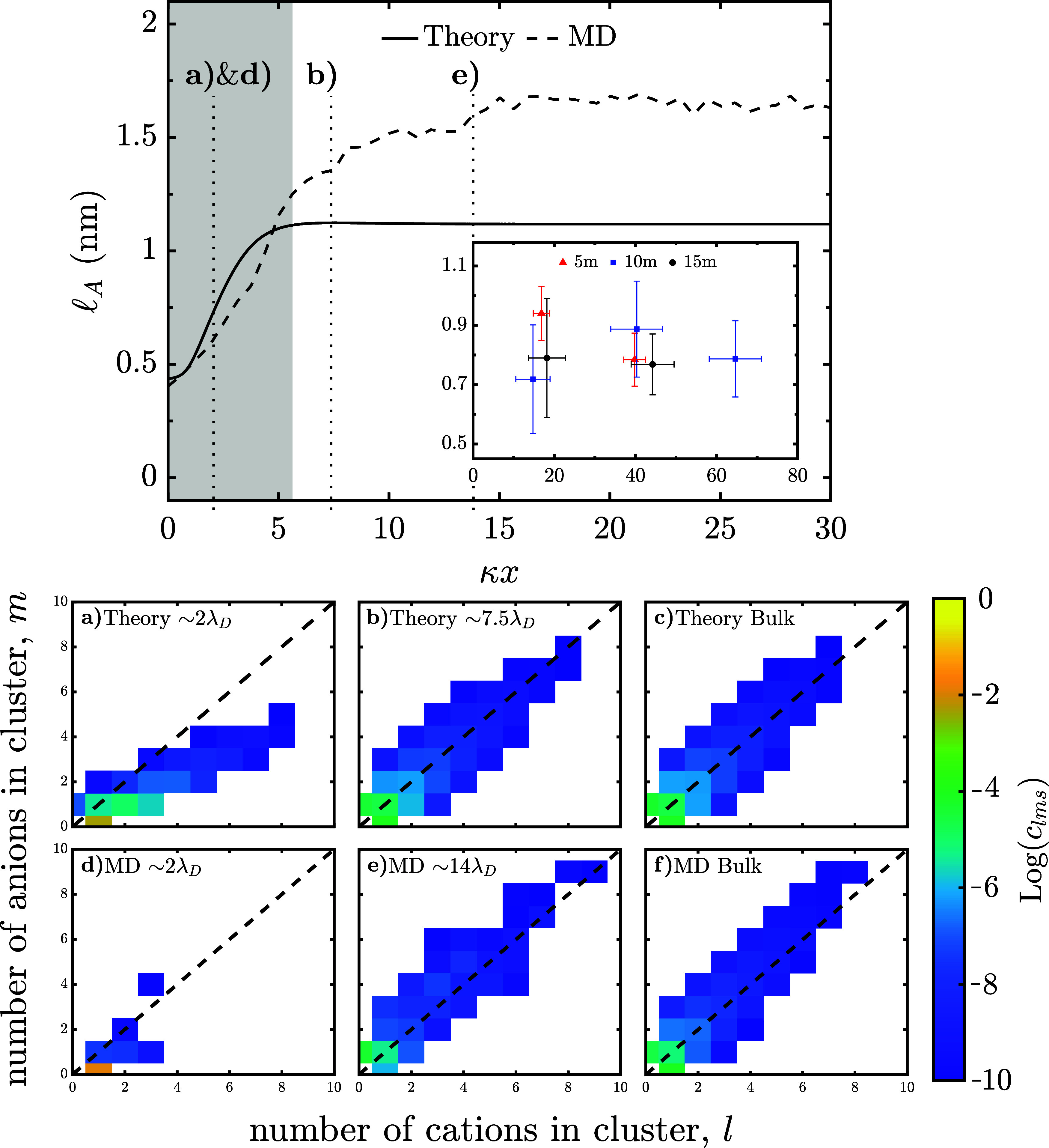
Aggregation length scale and cluster distributions for WiSEs in the EDL. Aggregate length scale of 15m water-in-LiTFSI at *q*_s_ = −0.2 C/m^2^ as a function of distance from the interface in dimensionless units, where κ is the inverse Debye length. The inset is the experimental aggregate length scale for water-in-LiTFSI at mica surface as a function of distance from the interface in dimensionless units.^19^ (a,d) Cluster distribution ∼ 2λ_*D*_ from the *q*_s_ = −0.2 C/m^2^ interface. (b) Theory cluster distribution ∼ 7.5λ_*D*_ from the *q*_s_ = −0.2 C/m^2^ interface. (c,f) Cluster distribution in the bulk. (e) MD cluster distribution ∼ 14λ_*D*_ from the *q*_s_ = −0.2 C/m^2^ interface. For the theory, we use *f*_+_ = 4, *f*_–_ = 3, ξ_0_ = 1, ξ_+_ = 0.4, ξ_–_ = 10.8, ϵ_*r*_ = 10.1, λ = 0.231, *P* = 4.995 D, and *v*_0_ = 22.5 Å^3^.

Turning to the inset of Figure 5, the experimental data^19^ predicts a lower length scale compared to the MD simulation and the theory. Moreover, the experiments show that clusters are located further away from the surface. Experimentally, this finding may be the result of AFM as the position of the surface has an uncertainty, meaning it is possible that the tip cannot displace the last layer of strongly bound cations. Additionally, the deviations might be due to a difference in the surface charge of mica compared to the surface charge in simulation and theory. One could expect the magnitude of the surface charge for mica to be up to 0.33 C/m^2^. The experimental data measures the distances in the force peak heights from the AFM/SFA measurements, which can be interpreted as representing the minimum size of the ions/clusters in that region that are squeezed out together. However, the experimental measurements in the diffuse portion of the EDL appear to suggest the presence of a local maximum in $l_{A}$ , indicating enhanced aggregation compared to the bulk before decaying toward the surface. The local maximum seen in 10m measurements^19^ is in line with this hypothesis. This qualitative comparison suggests an experimental agreement with the theory’s and MD simulation’s predictions of the local enhancement in associations near a negatively charged interface.

Moving to the bulk, one can compare the MD and the theory predictions for $l_{A}$ against previous experimental measurements from scattering^13^ and SFA.^19^ Here, we find that for 15m water-in-LiTFSI, the characteristic length scale of the clusters should be around 1–1.7 nm. This result is consistent with findings from scattering that found the length scale of nanoheterogeneity from 9 to 14m water-in-LiTFSI being around 1–2 nm,^13^ and this was later found to be consistent against SFA results.^19^ The deviation between the MD and theory predictions for the bulk can be understood through their respective cluster distributions, shown in Figure 5c for the theory and Figure 5f for the MD. Comparing Figure 5c and f, one can observe that the MD distribution has a slightly heavier tail than the theory. This deviation leads to the larger $l_{A}$ in the MD compared to the theory. Additional discussion about the deviations and bias in the bulk distributions is discussed in greater detail in the SI.

One of the powerful aspects of our theory is that we are able to investigate in more detail the predicted cluster distributions in different regions of the EDL. In our previous descriptions of the EDL we only described what happens to the aggregates and free species, as is often done, but we can resolve clusters in more detail. In Figure 5a,d & b,e we show the cluster distribution for the positions in the EDL of the negative electrode that are indicated in Figure 5. In Figure 5a,d, the cluster distribution is ∼2λ_*D*_ from the interface is considered. In Figure 5a the theory predicts that the cluster distribution here is strongly skewed toward positively charged clusters, with the majority of the clusters being hydrated cations. Similarly, in Figure 5d, the MD predicts a strong skew toward positive clusters with almost all of them existing as hydrated cations. Here we can observe why the theory & MD agree at the interface, as the majority of the clusters are the hydrated states. Additionally, analyzing the cluster distribution at the regions where *p̅*_+–_ *p̅*_–+_ are maximized can provide deeper insight into the underlying physics. In the theory, this occurs at ∼7.5λ_*D*_, which is shown with higher resolution in the SI. As shown in Figure 5b, the cluster distribution is slightly elongated compared to the theory’s bulk distribution as displayed in Figure 5c. In the MD, the maximum is at ∼14λ_*D*_. The MD’s cluster distribution shown in Figure 5e, is stretched compared to the MD’s bulk distribution as displayed in Figure 5f. In both cases, the theory and MD display elongated cluster distributions around the maximum in *p̅*_+–_ *p̅*_–+_, which indicates that larger clusters are more prevalent here compared to the bulk. This finding is consistent given the connection between $l_{A}$ and the association probabilities. One might note the lack of a clear maximum for $l_{A}$ in the MD that originates from the degree of hydration being variable in the MD compared to the theory.

Next, one can consider the aggregation length scale near a positively charged interface, as shown in Figure 6. The MD simulation predicts that $l_{A}$ fluctuates with a decaying trend before rapidly decaying in the condensed layer to its final value around the length scale of water molecules. The theory captures the general trend with both the final bulk and interfacial value deviating from the MD results. The bulk deviation between the theory’s and MD’s predictions comes from the heavier tail in the MD cluster distribution as discussed previously. The deviation at the interface could result from the distinct interactions expected at the interface. This reasoning aligns with the “hydration” layer seen in the MD, but not in the theory. This deviation is also reflected in the differences in the predicted cluster distributions, shown in Figure 6a for the theory and Figure 6d for the MD.

**Figure 6:**
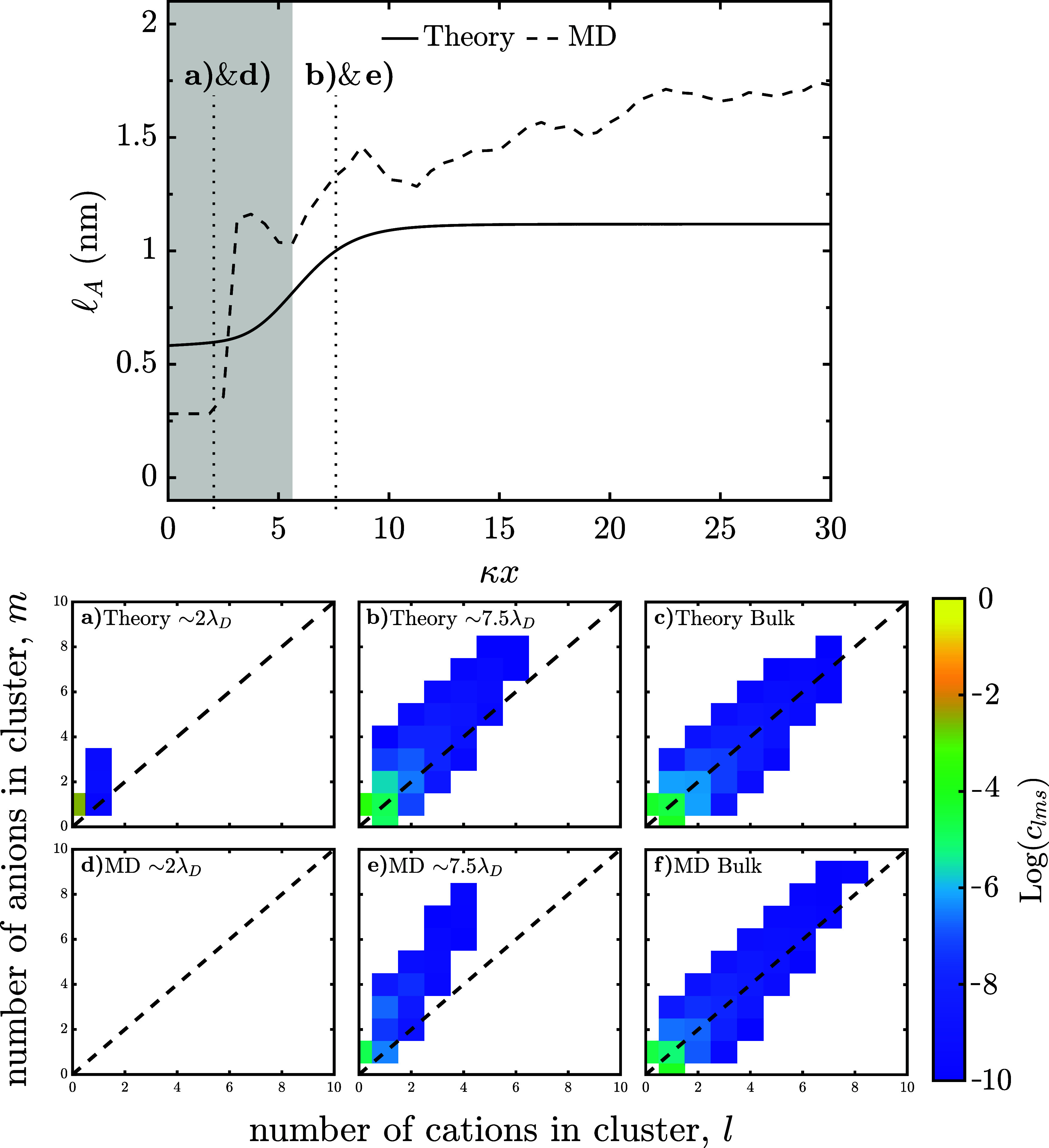
Aggregation length scale and cluster distributions for WiSEs in the EDL. Aggregate length scale of 15m water-in-LiTFSI at *q*_s_ = 0.2 C/m^2^ as a function of distance from the interface in dimensionless units, where κ is the inverse Debye length. (a,d) Cluster distribution ∼ 2λ_*D*_ from the *q*_s_ = 0.2 C/m^2^ interface. (b,e) Cluster distribution ∼ 7.5λ_*D*_ from the *q*_s_ = 0.2 C/m^2^ interface. (c,f) Cluster distribution in the bulk. For the theory, we use *f*_+_ = 4, *f*_–_ = 3, ξ_0_ = 1, ξ_+_ = 0.4, ξ_–_ = 10.8, ϵ_*r*_ = 10.1, λ = 0.231, *P* = 4.995 D, and *v*_0_ = 22.5 Å^3^.

Similar to the negative electrode, one can consider the changes in the cluster distribution as one approaches a positively charged electrode in Figure 6a,b for the theory, and in Figure 6d,e for the MD. The cluster distribution at ∼2λ_*D*_ from the interface is shown in Figure 6a,d, for the theory and MD respectively. In Figure 6a, the theory predicts that the clusters will mainly be free anions, with minor amounts of small negatively charged clusters present. However, from Figure 6d, the MD predicts no ions are present at ∼2λ_*D*_ from the interface reflecting the presence of the “hydration” layer discussed earlier. Further away from the interface at ∼7.5λ_*D*_ is depicted in Figure 6b,e, for the theory and MD respectively. In Figure 6b, the theory predicts the cluster distribution is slightly shifted in favor of negatively charged clusters, with the most common cluster being the free anion. From Figure 6e, the MD predicts the cluster distribution to be strongly shifted in favor of the negatively charged clusters, with free anions being most common. While the MD appears less elongated compared to the theory at ∼7.5λ_*D*_, the more substantial bias to negatively charged clusters leads to it having a larger $l_{A}$ compared to the theory. In both the theory and the MD, the cluster distribution at ∼7.5λ_*D*_ is shifted in favor of negatively charged clusters with the most common cluster being the free anion.

### Integrated Quantities

#### Differential Capacitance

Next, we can consider how the differential capacitance of water-in-LiTFSI varies as a function of electrostatic potential in the theory, as shown in Figure 7a. Here, we have introduced the α-parameter,^37^ which accounts for short-ranged correlations between ions and stretches the theory’s voltage range to that from MD simulations and experiments. We set α to be 0.1, which has proven to be a reasonable value.^38^ At low potentials in Figure 7a, we see that the theory predicts the differential capacitance for pregel water-in-LiTFSI at 12m and 15m takes on a strongly asymmetric camel shape,^30,31,47^ with the larger peak occurring in the negative potential and a satellite peak occurring at large positive potential. One can associate each of these peaks with distinct circumstances, visualized and discussed in greater detail in the SI. The large negative peak is associated with the cation enrichment and is further amplified by the enhanced dielectric function. The moderate positive peak is associated with anion enrichment, but lacks the same dielectric enhancement as seen in the negative peak. The satellite peak at large positive potentials is associated with water enrichment and dielectric enhancement. This water-induced asymmetric satellite is analogous to having hydrophilic anions or hydrophobic cations, which is the case discussed in previous work.^48^ In the previous work, Budkov et al.^48^ showed that the interactions from water with hydrophobic cations or hydrophilic anions lead to the asymmetric peak observed at low positive potentials.^49^ In our work, this peak emerges at a large positive potential as a result of initial water diminution at a low positive potential, followed by its enhancement at a large positive potential.

**Figure 7:**
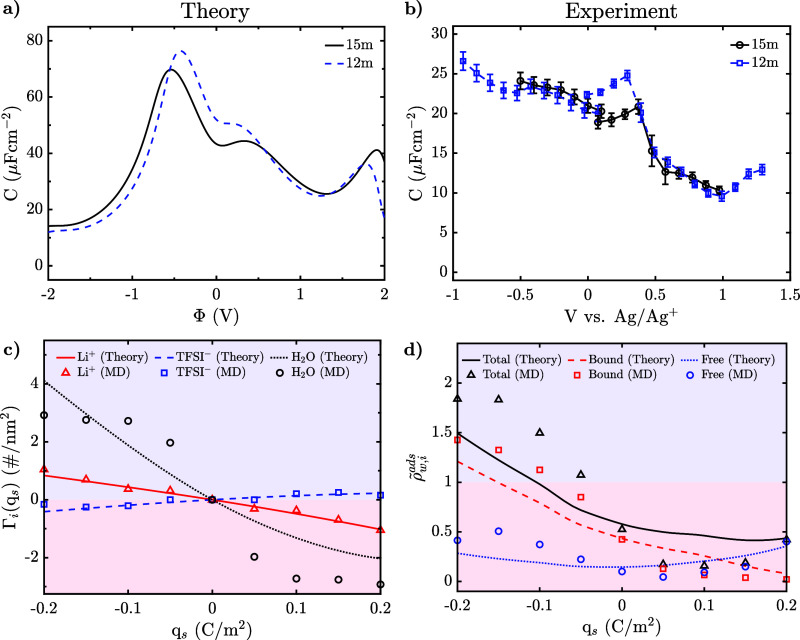
EDL predictions of WiSEs. (a) Theory prediction for the differential capacitance of water-in-LiTFSI as a function of the electrostatic potential, α = 0.1. (b) Experimental measurement of the differential capacitance of water-in-LiTFSI as a function of the applied voltage. (c) Excess surface concentrations for 15m water-in-LiTFSI as a function of surface charge. (d) Interfacial concentration of water for 15m water-in-LiTFSI as a function of surface charge. Here we use *f*_+_ = 4, *f*_–_ = 3, ξ_0_ = 1, ξ_+_ = 0.4, ξ_–_ = 10.8, ϵ_*r*_ = 10.1, and *P* = 4.995 D. For 15m, λ = 0.231 and *v*_0_ = 22.5 Å^3^. For 12m, λ = 0.226 and *v*_0_ = 22.9 Å^3^.

Additionally, in Figure 7a, one can consider the effects of concentration on the predicted differential capacitance from the theory. Here, we see that increasing the molality stretches out the profile horizontally, as seen from the peaks of the camel shape separating in voltage and the water-satellite peak moving to a higher voltage. Moreover, the amplitude of the camel valley in the differential capacitance increases with increasing concentration, causing a larger camel shape. These features are consistent with the differential capacitance curve becoming more camel-like, which has been correlated with a decrease in the number of free charge carriers in the electrolyte.^35^ Additionally, we see the water-satellite peak’s amplitude increase with molality. Molalities effect on the screening length, and therefore the Debye capacitance, is discussed in the SI in more detail.

Here we also experimentally investigated the differential capacitance of water-in-LiTFSI, at different molalities, as a function of the applied voltage, as shown in Figure 7b. The differential capacitance was extracted from electrochemical impedance spectroscopy (EIS) measurements performed in a three-electrode cell, where we started from the open circuit potential and went to positive and negative applied voltages. By conducting cyclic voltammograms for LiTFSI at various concentrations, we were able to ensure our EIS measurements were collected within the electrochemical stability window. The differential capacitance was extracted by analyzing EIS data using three different methods, two Nyquist plot-based fitting methods and a Cole–Cole plot-based fitting method, all of which produced similar trends. Shown in Figure 7b is the extracted differential capacitance by fitting to the equivalent circuit, as first introduced by Brug et al.,^50^ in the limit of dominant double layer resistance. For the differential capacitance predictions using the other methods, see the SI. The experimental protocol and analysis, along with predictions for 1m and 21m LiTFSI, are discussed in-depth in the SI.

In Figure 7b, one can note that the experimental differential capacitance for both 12m and 15m water-in-LiTFSI appears to have a camel-shaped curve. At both concentrations, 12m and 15m, we see a peak at moderately positive applied potentials at 0.29 V vs Ag/Ag^+^ and peaking around 24.78 μF cm^–2^ and at 0.37 V vs Ag/Ag^+^ and peaking around 20.85 μF cm^–2^ respectively.^30^ As the concentration increases, the magnitude of the differential capacitance curves decreases slightly everywhere, but the positive peak at 15m is smaller than the 12m. Additionally, both the 12m and 15m differential capacitance curves appear to decay monotonically after their positive peak, with some increase being measured at large positive potentials, and the differential capacitance profile appears to increase with increasingly negative applied potentials. As these profiles are collected within the electrochemical stability window, this increase in differential capacitance at negative applied potential is believed to be part of a larger negative peak at a larger negative potential. This result suggests the differential capacitance has a camel-shaped curve centered close to 0 V vs Ag/Ag^+^ or at slightly negative applied potential.

Now, one can compare the theory’s predictions against experimental data for a more robust evaluation of the theory. Here, we find that in both the theory in Figure 7a and the experimental data in Figure 7b, as the molality of the solution increases, the magnitude of the differential capacitance decreases. This effect is clearest in the positive potential peaks. The general shape of the differential capacitance between the theory and the experiments suggests a camel shape with a water-satellite peak at a large positive potential. Note that the most prominent peak in the theory, found at negative potentials, is not explicitly seen in the experiments. However, the peak’s existence is reflected in the increasing differential capacitance at increasing negative applied potentials that suggests its presence at larger negative potentials. Moreover, comparing the 1m case results for theory and experiments in the SI shows the absence of the positive differential capacitance peak, further supporting that this experimental peak corresponds to the positive peak in the theory’s prediction around 0.5 V. Lastly, the magnitude of the differential capacitance differs between the theory’s predictions and the experimental data, which can be attributed to the assumptions of the employed theory (discussed later), not fitting the α and not including a Stern layer while calculating the capacitance. These deviations are expected given the sophisticated nature of obtaining differential capacitance profiles experimentally and a well-known weakness of simple mean-field models, both of which are discussed in greater detail in the following section. Overall, we observe similar trends in the differential capacitance profiles in the theory and the experimental measurements.

#### Excess Surface Concentration

For reactive interfaces, understanding the excess surface concentrations, defined by eq 28, can provide insight into the local reaction environment.^11^ The excess surface concentrations from MD simulations and the theory’s predictions for 15m water-in-LiTFSI at a variety of surface charges are shown in Figure 7c. For Li^+^, the MD simulations predict that its concentration will be enhanced at negative surface charges and diminished at positive surface charges. This prediction for Li^+^ is accurately captured by the theory. For TFSI^–^, the MD simulations predict that the excess surface concentration will be diminished at negative surface charges and enriched at positive surface charges. Additionally, the excess TFSI^–^ surface concentration will achieve its maximum value of reduction around −0.15 C/m^2^ and the maximum enhancement around 0.15 C/m^2^. The theory can accurately capture the general trend for the modulation of excess surface TFSI^–^ although it does miss the local maximum and minimum observed from the simulations. Lastly, water is predicted to accumulate strongly at negative surface charges and be depleted at positive surface charges. Once again, the theory captures the general trend with strong enrichment and reduction at the respective surface charges. Overall, the theory appears to be capable of capturing the general trends in the accumulation and depletion of each species near the surface.

#### Interfacial Concentration of Water

Lastly using the results from our MD simulations, we tested the theory’s prediction for the interfacial concentration of water, defined by eq 29 and shown in Figure 7d. From the simulations, one observes the asymmetric response in the system, which is expected from the excess surface measurements, and the existence of a depletion region near each charged interface. At increasingly negative surface charges, the total amount of water found close to the interface is enriched compared to the bulk concentration in the simulations. At increasingly positive surface charges in the simulations, the average total water concentration gradually diminishes before increasing moderately around 0.1 C/m^2^. Note this later moderate enhancement in total water and, more importantly, free water at larger positive potentials is consistent with the water-satellite peak found at large positive potentials in the theory and experiments. In Figure 7d, the theory predicts the total amount of water increases with increasing negative surface charge, becoming enriched at a later point than seen in the simulations. Similarly the theory predicts the average total concentration of water is diminished at increasing positive surface charge. Hence, the theory appears to predict the qualitative trends seen in the MD simulations of the interfacial concentration of total water while deviating from the quantitative values and local minimum at moderate positive surface charges. Strengthening this finding, one can turn to prior experimental studies of WiSE using Surface-enhanced infrared absorption spectroscopy (SEIRAS), which can probe species enrichment near charged interfaces.^18,51^ From these experiments, they found that in WiSE less water at positive than negative potentials,^18^ and a similar result was found for WiSE with divalent cations.^51^ Therefore, the overall enrichment (depletion) at a negatively (positively) charged interface from the MD and theory is in qualitative agreement with experimental results obtained by SEIRAS.^18,51^

We can further decompose the total water into that bound to Li cations and free water. In Figure 7d from the MD results, the amount of bound water monotonically increases at increasingly negative surface charges and obtains a larger average concentration than bulk water around −0.1 C/m^2^. For increasing positive surface charges the amount of bound water slowly decreases monotonically. Similarly, the theory predicts the monotonic increase in the amount of bound water near the increasingly negatively charged interface and its decrease near the increasingly positively charged interface. These qualitative trends agree with the MD simulations. The modulation of interfacial free water from MD and theory can also be seen in Figure 7d, wherein the MD simulations it increases with increasing negatively charged interfaces with a maximum near −0.15 C/m^2^ after which it slightly decreases. Considering now increasing positive surface charges, the amount of free water initially decreases before rapidly increasing and becoming the majority of the total water near the interface. The theory predicts as the surface becomes increasingly negatively charged, the interfacial concentration of free water shows a slight decrease with a minimum at −0.01 C/m^2^ followed by a monotonic increase. For increasing positive surface charge, the theory predicts a monotonic increase in the amount of free water. The theory adequately predicts the qualitative trends for the amount of interfacial free water. Overall as shown, the theory appears to capture the qualitative trends from the MD simulations for the interfacial concentration of water of 15m water-in-LiTFSI. Note that the amount of free water becomes dominant to the bound water at 0.1 C/m^2^ in the MD results and a little after 0.1 C/m^2^ in theory, which further demonstrates the utility of the theory in capturing the qualitative trends and behavior of WiSEs.

## Discussion

Through testing our theory against MD simulations, we have observed the limitations of the theory in capturing overscreening and nonlocal effects as well as surface effects. These are expected technical limitations from the theory presented here and are seen through the theory’s predictions deviating quantitatively and from the finer structure of the MD profiles. Additional conceptual and technical limitations of this style of theory are outlined in ref 27.

The first category of limitations are nonlocal effects and overscreening. Overscreening is the phenomenon where an excess amount of counterions are pulled into the EDL, leading to a layer of co-ions being dragged into the EDL to compensate for their excess charge. Conceptually, overscreening is a representation of thermo-reversible associations in the WiSE, as the alternating structure of cations and anions is similar to the layering ions in overscreening.^40^ However, the internal structure of the clusters is not explicitly modeled in our theory, leading to layering of ions being averaged-out. Hence we do not explicitly obtain decaying oscillations in the charge density as the result of the theory’s simple construction. Generally, this limitation is reflected in the theory being unable to capture the oscillations, local maximums, and specific layering associated with overscreening given the simple local point-like formulation of the modified PB equations, which can be accounted for with more intricate and nonlocal approaches.^22,31,33,34,40,41^ The importance of the interplay between associations, overscreening, and steric effects was highlighted in ref 40 and discussed in ref 27. Developing more sophisticated theories that are able to succinctly capture and balance the short-ranged associations, nonlocal correlations, steric effects, and surface-specific interactions will be crucial for further resolving concentrated electrolytes such as WiSEs near charged interfaces.

The nonlocal effects may also be critical to capturing the consequences of the finite nature of these clusters. For example as shown in Figure 3h, the theory prediction for the product of the ionic association probabilities, *p̅*_+–_ *p̅*_–+_, is smaller than from the simulation in Figure 3d. This deviation could be an artifact of neglecting nonlocal effects by treating clusters as points. Additionally, these effects can lead to nonmonotonic electrostatic potential. Now, considering that WiSEs are expected to display some induced associations at a slightly negative potential, these oscillations in potential could lead to electric field induced associations occurring at both charged electrodes. This effect could, in fact, be seen in the MD simulations in Figure 4d, where the fluctuations from nonlocal effects and overscreening are captured. This may explain the regions of induced associations seen in Figure 4d, which depicts *p̅*_+–_ *p̅*_–+_ in the EDL near a positively charged electrode.

The most significant limitation in our theory comes from the overly short screening length, λ_*s*_, being predicted as ∼1.1 Å for 15m water-in-LiTFSI. This λ_*s*_ is smaller than an individual Li^+^ (∼1.6 Å), and this is much smaller than the length scale of aggregates in the system in the theory for 15m water-in-LiTFSI being ∼11 Å (∼16 Å from the simulations). This result suggests that the theory is not acting self-consistently in regard to its electrostatic predictions; this is a well-known challenge of mean-field lattice gas models and is discussed in detail in ref 31. This common failing of mean-field theories can be corrected through more sophisticated modified PB equations that can capture higher-order correlations and nonlocal effects, or partially corrected through introducing α.^37^ This inconsistency can also be addressed to some degree by modifying local mean-field models, such as by including higher order local terms, as done in BSK theory,^22^ or by modifying the Coulomb interactions.^41^ However, to overcome this inconsistency, one typically needs to employ a nonlocal model that can capture the entropic effects of excluded volume in a holistic fashion. This inconsistency suggests using caution when using the theory’s predicted spatial profiles of its species and cluster near the charged interface.^31^ However, as discussed earlier from a qualitative perspective, the theory can capture the trends and overarching behavior seen in the spatial profiles of species and clusters near the charged interface, despite lacking the sophisticated nonlocal effects and overscreening seen in MD simulations. Moreover, a valuable power of mean-field models is their tendency to adequately predict integrated quantities even with mean-field models’ inconsistencies. For this reason, one could expect the theory’s predictions of the double-layer capacitance, excess surface concentrations, and interfacial concentration of water to be reasonable, as we demonstrated. Furthermore, one could expect, as demonstrated, the general trends in WiSE properties in the EDL to be qualitatively captured along with the length scale of aggregates and, most importantly, how the association probabilities change *within* the EDL.

The other category of limitations are introduced by surface effects which may dominate the physics in the condensed layer. Even though the theory can reasonably describe the diffuse EDL, it cannot capture the condensed layer where there are interfacial layers of ions and water. This breakdown is expected as the cluster distribution should deviate from the diffuse and bulk as a result of the specific interactions with the electrode creating a significant change in the coordination and cluster distribution. In short, in the condensed region, the local solvation environment is expected to be disrupted by the interaction with the interface. These changes can be directly seen by the gray regions representing the condensed layer from the MD simulations in the left-hand column of Figures 3 and 4. However, the intricacies of the condensed layer seen in our MD simulations could be further compounded by the lack of sufficient statistics for co-ions in the EDL.

Experimentally benchmarking any theory or simulation is desirable and timely as different approaches will capture different properties of the system. In this current work, we have been able to test the theory’s prediction of the aggregate length scale and differential capacitance against experimental data. The aggregation length scale was obtained via the extraction of an estimate of the average length scale of the nanoheterogeneities in bulk solution through AFM.^19^ Besides the experimental data highlighted here, AFM measurements^18^ on gold positively biased (+0.3 V) and 21m LiTFSI show the presence of clusters with a max size of 0.8 nm close to the surface, then with sizes ranging from 0.3 to 0.6 nm closer to the surface. This finding indicates our results are in qualitative agreement with additional experimental studies,^18^ although data at lower concentrations have yet to be obtained. Scattering experiments provide an alternative way to measure cluster sizes and the average length scale of nanoheterogeneities in the bulk.^13,15,52^ This previous experimental data,^19^ supported the theory’s prediction that negatively charged interfaces can lead to an enhancement in associations along with the qualitative trends in the aggregation length scale itself compared against MD simulations and the theory. Additional experimental investigations for a more diverse set of electrolytes could provide novel insights into the role of associations and, in turn, the role of the local solvation structure on interfacial electrochemical reactions and operation of energy storage devices, using traditional to concentrated electrolytes.

Comparing the theory’s predicted differential capacitance against the experimental data gave a promising conclusion. Here, we found that some of the trends seen in the theory’s predictions of the differential capacitance held such as displaying camel shaped profiles with a water-satellite and the decreasing magnitude of the profiles from 12m to 15m water-in-LiTFSI. The main challenge is that a rigorous extraction of the differential capacitance profiles requires a sophisticated approach incorporating the physical details and structuring of the EDL into its differential capacitance fitting. While the current analysis here has focused on extracting trends and qualitative profiles, a more quantitative and detailed investigation into these EIS measurements could be deeply illuminating. In general the difficulty in obtaining robust and exact differential capacitance profiles is well established. Beyond this challenge, the choice of electrode material and surface roughness is expected to affect the differential capacitance measurement. In the SI, these effects are highlighted. Given the simple nature of the model, it is expected to fail in terms of the absolute value as seen for the overall differential capacitance curves; however as discussed, the model’s ability to predict the general trends is desirable. Additionally, the structuring of the condensed layer is expected to play an important role in the overall capacitance measurements. Hence the condensed layer acts as one limiting factor to the current theory. Even with these deviations, the ability of the simple theory to capture some of the key trends and structure of differential capacitance remains promising.

The potential utility of the perspective our theory provides may extend beyond the limited experimental analysis shown here. Recently various investigations into the behavior of concentrated electrolytes near interfaces have worked to resolve experimentally and computationally the mystery beyond the unique interfacial properties.^10,19,21,53^ A common theme throughout these investigations arrive at is the importance of the local structuring, orientation, and interactions near the electrode. This idea is a core element of our theory. While the model is expected to fail in some ways the value in it is the first-principles intuition it provides, which appears to be shared by top-down investigations. This perspective may aid in understanding experimental findings and improving electrolyte design.

### Implications for Energy Applications

Understanding the structure of the EDL is essential for capturing the equilibrium properties of WiSEs and developing deeper insight into interfacial reactions occurring at electrode surfaces as well as the stored charge.^2,5,8,9,11,12^ Regarding the former, there is a strongly asymmetric response of the water, where it mainly depletes at the cathode side giving rise to the extended cathodic stability, but accumulates on the anode side. However, the increase in water on the anode side mainly corresponds to bound water, and McEldrew et al.^12^ found that solvated water has a lower activity, which means it is less likely to react. However, at very large potentials (both positive and negative), there is eventually an increase in the interfacial free water that may be able to react. These findings are in line with the current understanding behind the expanded ESW in WiSEs.^10,11^

Additionally, the theory captures the association probability of the species in the EDL providing insight into the local solvation environment. The species at the interface are precursors for interfacial reactions. This information on the local solvation environment and species activities could provide deeper insight into which species are likely to undergo decomposition into the SEI.^54^ For example, we have found, from both theory and simulation, that at the anode there is a slight increase in aggregation at moderate potentials. Previously, McEldrew et al.^12^ found that the activity of the salt increases with concentration/aggregation, which suggests that these additional aggregates could assist in the formation of the passivating SEI layers. Understanding which species are contributing to the formation of the SEI could support rational electrolyte design.^12,54–56^ By improving electrolyte formulation, the passivation layers produced could be more efficient and effective. Furthermore, aggregation occurring close to the electrode/electrolyte interface may also have implications for the metal cation mobility. Additional aggregation of the electrolyte and the exclusion of the IL cation from the anode can help hinder dendritic growth and form a more compact, homogeneous and stable SEI.^57^ Moreover, our theory’s ability to provide a deep understanding of how these cluster distributions are modulated in the EDL, as well as by the composition and other experimentally tunable variables, makes it valuable for better understanding the role of electrolytes’ local solvation structure in energy storage.

The existing studies, however, do not address how the unique microenvironment experienced by the interfacial ions and water molecules affects their reactivity and charge transfer with the electrode and other molecules. Recent experiments have revealed that the effect of the WiSE EDL on the interfacial reactivity of redox species is significant.^18,51^ These works used an ultramicroelectrode to carry out CVs, enabling faster diffusion and a higher sensitivity to the faradaic reaction. In the WiSE LiTFSI, the CVs showed a peak on the anodic scan attributed to the oxidation of Fe­(CN)_6_^4–^. This peak was not present in 1m LiTFSI, which indicates that the interphase concentration of Fe­(CN)_6_^4–^ is greatly increased in the WiSE, and is attributed to the confinement effect provided by the WiSE at the interface with the electrode. They also found that the addition of Zn^2+^ led to a decrease of the surface-confined peak.^18^ The findings suggested that the confinement effect is reduced by Zn^2+^ and is enhanced by Ca^2+^. The results suggest that the WiSEs EDL and structure could be a tool to enable selectivity and tunability of interfacial reactions.

The screening length determines the stored charge in the EDL and it is a topic of controversy in the context of highly concentrated electrolytes. Experimental surface force measurements have found that concentrated electrolytes, such as in ionic liquids and water-in-Salt electrolytes, have extremely long force decay lengths.^19,52,58–60^ Gebbie et al.^58^ asserted that these forces were electrostatic in origin, and arose from the large renormalization of the concentration of free charge carriers. This statement would imply that the screening length is about 1–2 orders of magnitude larger than the Debye length. This phenomenon was originally named underscreening, but, as the topic is controversial and unresolved,^61,62^ it has been further classified as anomalous underscreening.^63,64^ This refinement was implemented as other experiments,^19,52^ simulations,^61,65^ and theories^27,37,40^ have been able to capture an uptick in the screening length. However, the scaling seen in these works is less than originally reported.^58,66^ Moreover, as we show, we only find a modest increase in the screening length, still remaining smaller than 1 nm, which does not suggest these long force decay lengths solely arise from electrostatics. Even with the lack of consensus through studying the aggregation of ions and decoration by solvent, this approach has been widely successful in capturing the bulk and transport properties in WiSEs^12^ and concentrated electrolytes.^25^ Recent studies^29,52,67^ have been converging toward an alternative hypothesis that it is steric interactions, also known as hard-core interactions, contributing toward the long-ranged interactions seen in various concentrated electrolytes and not a purely electrostatic phenomenon.^62,68^ We believe the theory presented here could aid in further resolving these kinds of measurements,^19,52,58–60^ as discussed in ref 29.

While our analysis here, has focused on the equilibrium properties and structuring of WiSEs, one can expand these results and theory to capture dynamics. The theory can be extended to investigate WiSE’s transport properties by adapting the methodology outlined in ref 24. Moreover, as WiSEs have an active cation for intercalation, expanding the theory of coupled ion electron transfer reactions to incorporate the local solvation environment could be noteworthy.^69^

Finally, the mathematical analysis required for this theory extends beyond even the most advanced theories for patchy particle systems,^70^ while also taking into account electrostatic interactions. These extensions could be used to account for how other interactions or drivers of concentration localization influence the cluster of polymers. For example, in 3D-printing under electric fields or in synthesis requiring sol–gel equilibrium’s. While our theory borrowed core elements of polymer physics initially, the extensions seen here could find broader applications in polymer physics and other statistical physics.

## Conclusions

Here, we have developed a theory for EDL of WiSEs that accounts for thermoreversible associations, based on McEldrew et al.’s model for bulk WiSEs. We thoroughly tested this theory against MD simulations and found good qualitative agreement for many cases, such as the distributions of total species, the distribution of specific clusters (free species, hydrated cations, and multi-ion aggregates), and association probabilities. Additionally, our theory’s prediction for integrated quantities such as the excess surface concentrations and the interfacial concentration of water were found to be in reasonable agreement with MD simulations. This simple theory’s value is its ability to capture how the associations *within* the EDL change, not previously quantified with any theory, allowing more detailed predictions of cluster distributions and ionic network formation. We found that the way cluster size changes in the EDL is similar to the changes seen in AFM measurements and matches the qualitative trends in MD simulations. Moreover, our theory’s prediction of the differential capacitance was found to be in reasonable agreement with our experimental results. The mathematical analysis here goes beyond even the most advanced theories for patchy particle systems, in addition to taking into account electrostatic interactions. These ideas might find applications in other fields of statistical physics as well as in understanding experimental results.

As WiSEs are an exciting class of electrolytes for energy storage, with lots of simulations and experiments investigating these systems for a myriad of applications, having a theory to build intuition is critical. This work can be used for the following: provide insight into the local structure through the ionic aggregation and solvation of species near electrodes, aid in predicting the formation of the solid electrolyte interphase (SEI), and shed light on surface force measurements near electrified interfaces. Overall, the applications of this theory are extensive and we hope it will inspire additional studies into the interfacial behavior of electrolytes as well as support rational electrolyte design. Looking forward, we believe developing the approach to understand the kinetics of solvation/desolvation, charging dynamics, and coupled-ion-electron transfer reactions at interfaces could be interesting areas for exploration.

## Supplementary Material



## Notes

Discussion of the theory and its evaluation, the molecular dynamics simulation methodology, the data analysis for the simulations, an additional comparison of theory as well as approximations, the experimental methods, and additional predictions and discussions of results from the theory and comparison of theory and experimental data (PDF)
