# Supplementary material for: Ionic Associations and Hydration in the Electrical Double Layer of Water-in-Salt Electrolytes
Source: ACS Appl Mater Interfaces. 2025 May 7;17(20):29515–34. doi: 10.1021/acsami.5c01781 (PMC12100604; doi:10.1021/acsami.5c01781)
Supplement: Supplementary file 1 [file am5c01781_si_001.pdf]

Supporting Information - Ionic Associations and Hydration in the Electrical Double Layer of Water-in-Salt Electrolytes

Daniel M. Markiewitz,[1] Zachary A. H. Goodwin,[2,3,*] Qianlu Zheng,[4] Michael McEldrew,[1] Rosa M. Espinosa-Marzal,[4,5] and Martin Z. Bazant[1,6,†]

[1]*Department of Chemical Engineering, Massachusetts Institute of Technology, Cambridge, Massachusetts 02139, USA*

[2]*John A. Paulson School of Engineering and Applied Sciences, Harvard University, Cambridge, Massachusetts 02138, United States*

[3]*Department of Materials, University of Oxford, Parks Road, Oxford OX1 3PH, United Kingdom*

[4]*Department of Civil and Environmental Engineering, University of Illinois Urbana–Champaign, Urbana, IL, 61801 USA*

[5]*Department of Materials Science and Engineering, University of Illinois Urbana–Champaign, Urbana, IL, 61801 USA*

[6]*Department of Mathematics, Massachusetts Institute of Technology, Cambridge, Massachusetts 02139, USA*

(Dated: April 16, 2025)

[*] zac.goodwin@materials.ox.ac.uk

[†] bazant@mit.edu

I. EXTENDED THEORY SECTION

In this section, the theory is discussed in greater detail, along with its implementation, and additional results.

A. Sticky Cation Approximation Bulk Bias Derivation

Here we prove in the bulk how the functionality of cations being larger than anions leads to the clutter distribution being marginally biased towards net negative clusters. The analysis presented holds for both the pre-gel and post-gel regimes. However in the post-gel regime, the individual bias of the clusters in the solution phase and the gel phase represents an additional level of resolution one could consider. To understand this result, in the sticky-cation formalism, one can note that any cation that is not in its hydrated state (c_{10f_+}) must belong to a multi-ion cluster. This likewise holds for the free anions (c_{010}). This implication means that the ratio of positive to negative charges stored in the clusters will be inversely proportional to the ratio between the hydrated cations and free anions (c_{10f_+}/c_{010}). Hence if c_{10f_+}/c_{010} is equal to 1 the clusters have a net neutral bias, greater than 1 the clusters have a net negative bias, and less than 1 the clusters have a net positive bias. This result can be intuitively tested through limiting cases. First, consider the case where c_{10f_+}=c_{010}. Here, there are an equal number of cations and anions found in clusters as in the bulk c_+=c_-. Hence, one can conclude that the multi-ion clusters must contain equal amounts of cations and anions, i.e. the clusters would have no net charge bias. Second, consider the case where c_{10f_+} is nonzero and c_{010}=0. Here, there are more anions in the clusters than cations. From this implication, one can conclude that the multi-ion clusters will have a negative bias. Lastly, consider the case where c_{10f_+}=0 and c_{010} is nonzero. In this case, there will be more cations in the clusters than anions. This implication allows one to conclude that the clusters will have a positive bias. Note that this ratio is analogous to the Boltzmann closure relationships, but here we can see it can provide additional insightful information on the bias of the multi-ion clusters. In general, one should distinguish between the gel and the multi-ion clusters as the gel is its own phase. For this reason, we will restrict the analysis here to the pre-gel regime for simplicity, although this form of analysis could be extended for additional information.

At this point, we can begin our proof that for water-in-salt electrolytes (WiSEs) in the pre-gel bulk, under the sticky-cation formalism, the net charge bias of the multi-ion cluster depends solely on the ratio of the anion to cation functionalities. First, recall what we just proved for the values of c_{10f_+}/c_{010}:

$$\frac{c_{10f_+}}{c_{010}} = \begin{cases} \text{multi-ion clusters have net positive bias,} & < 1 \\ \text{multi-ion clusters have no net charge bias,} & = 1 \\ \text{multi-ion clusters have net negative bias,} & > 1 \end{cases} \tag{S1}$$

To make any additional discernment one must expand c_{10f_+} and c_{010} where we use their volume fraction equations and convert them into concentration,

$$\frac{c_{10f_+}}{c_{010}} = \frac{c_+(1-p_{+-})^{f_+}}{c_-(1-p_{-+})^{f_-}} = \frac{(1-p_{+-})^{f_+}}{(1-p_{-+})^{f_-}}. \tag{S2}$$

To resolve this question, convert Eq. (S2) into an equation that depends only on one unknown. Here we choose p_{-+}, where p_{+-} is eliminated through recalling and using the conservation of cation-anion associations in the bulk will be $f_+p_{+-} = f_-p_{-+}$,

$$\frac{c_{10f_+}}{c_{010}} = \frac{(1-\frac{f_-}{f_+}p_{-+})^{f_+}}{(1-p_{-+})^{f_-}}. \tag{S3}$$

To proceed, one must recall that taking the logarithm of c_{10f_+}/c_{010} and multiplication by a strictly positive constant will preserve order,

$$\frac{1}{f_+}\log\left(\frac{c_{10f_+}}{c_{010}}\right) = \begin{cases} \text{multi-ion clusters have net positive bias,} & < 0 \\ \text{multi-ion clusters have no net charge bias,} & = 0 \\ \text{multi-ion clusters have net negative bias,} & > 0 \end{cases} \tag{S4}$$

This allows us to rewrite Eq. (S3) to be only a function of p_{-+} and f_-/f_+,

$$\frac{1}{f_+}\log\left(\frac{c_{10f_+}}{c_{010}}\right) = \log\left(1-\frac{f_-}{f_+}p_{-+}\right) - \frac{f_-}{f_+}\log\left(1-p_{-+}\right). \tag{S5}$$

Now for the last key step, one must observe that the derivative of Eq. (S5) with respect to p_{-+}, is monotonic where it's defined on our regime, i.e. $p_{-+} \in [0, \min(f_+/f_-, 1)]$,

$$\frac{\partial}{\partial p_{-+}}\left(\log\left(1-\frac{f_-}{f_+}p_{-+}\right) - \frac{f_-}{f_+}\log\left(1-p_{-+}\right)\right) = \frac{f_-}{f_+}\left(\frac{1}{1-p_{-+}} - \frac{1}{1-\frac{f_-}{f_+}p_{-+}}\right) \tag{S6}$$

The monotonicity of Eq. (S6) will be critical. To see this first, it is helpful to note that not that when Eq. (S5) is zero when p_{-+}=0. Hence as Eq. (S6) is monotonic in the domain of interest, this implication requires that the sign of Eq. (S5) is determined by the sign of Eq. (S6). The sign of Eq. (S6) is clearest at its upper bound, i.e. $p_{-+} = \min(f_+/f_-, 1)$. Here Eq. (S6) will only not diverge if $f_+/f_- = 1$, meaning that in this case Eq. (S5) is zero throughout its domain, i.e. the multi-ion clusters have no net bias. Now, let us consider the cases where Eq. (S6) diverges to $\pm\infty$. Here, if $f_-/f_+ > 1$ then the second term will diverge first with increasing p_{-+}, meaning Eq. (S6) diverges to $-\infty$. This result means that Eq. (S5) will decrease as p_{-+} increases, i.e. Eq. (S5) will be less than zero throughout its domain besides at p_{-+}=0. This finding means that for $f_-/f_+ > 1$ multi-ion clusters will have a net positive bias. Now, let us consider if $f_-/f_+ < 1$ then the first term will diverge first with increasing p_{-+}, meaning Eq. (S6) diverges to ∞. This result means that Eq. (S5) will increase as p_{-+} increases, i.e. Eq. (S5) will be greater than zero throughout its domain besides at p_{-+}=0. This finding means that for $f_-/f_+ < 1$ multi-ion clusters will have a net negative bias. From this we can conclude,

$$\text{multi-ion clusters charge bias} = \begin{cases} \text{net positive}, & \frac{f_-}{f_+} > 1 \\ \text{net neutral}, & \frac{f_-}{f_+} = 1 \\ \text{net negative}, & \frac{f_-}{f_+} < 1 \end{cases} \tag{S7}$$

Therefore, as $f_+ = 4$ and $f_- = 3$ in the main paper, the distribution beyond the hydrated lithium and the free $TFSI^-$ is expected to be marginally biased towards net negative clusters. Note in our proof for the bulk under the sticky-cation formalism there are two main edge cases from limiting conditions. Both cases result in multi-ion clusters being ill-defined or neutral bias; if one asserts no multi-ion clusters being present means they have net neutral bias. First, if any ionic species functionality is zero, then there are no multi-cluster ions, so this measure is ill-defined. Second, if p_{-+}=0, it would appear that there is no net bias. However considering p_{-+}=0's implications, this means there are no multi-ion clusters present, which means this measure is ill-defined once again. In both of these edge cases, they occur when no associations are present, which means for all enlightening parameter choices for our theory, this measure is informative and applicable.

Alternatively, one can qualitatively determine the sign and relative degree of the multi-ion cluster charge bias graphically by finding how unbalanced the cluster distribution is from the

neutral line, i.e. clusters with the same number of cations and anions. For minor deviations, this can be hard to visually see in the cluster distribution graph. To support this graphical method, one can perform a weighted least squares fit to the function $m = slope * (l - 1) + 1$, where the weights are the value for c_{lms} and we exclude the free species as we want to understand the multi-ion clusters charge bias. This definition still works in the non-sticky and MD simulations, as the varying degree of hydration acts as additional points and weights to the regression. The reason for fixing the line at (1,1) is that it simplifies the graphical analysis; if the slope is greater than 1 it is negatively biased, and if it is less than 1 it is positively biased. Furthermore, its limits agree with intuition, as a slope of zero implies the maximum positive bias of 1 anion to always more than 1 cation, and similar to an infinite slope that implies the maximum negative bias of 1 cation to always more than 1 anion. We demonstrate this in Fig. S1 for 15m water-in-LiTFSI for both theory and MD. In this figure, we can see in both cases the slopes are greater than 1 indicating a negative multi-ion charge bias and that this bias is more prevalent in the MD simulations. This deviation is expected as the theory treats the individual species as point particles; whereas, the MD has real molecules and since the $TFSI^-$ molecule is large and expansive, intuitively it is consistent with further promoting clustering and aggregation occurring, meaning the clusters would be even more negatively biased than the theory would expect. Of course, this is not a quantitative measure to gain that one should use the quantitative equation, $(c_+ - c_{10f_+})/(c_- - c_{010})$ for the cluster bias and c_{10f_+}/c_{010} for the free species bias. For the theory in 15m water-in-LiTFSI, $c_{10f_+}/c_{010} = 1.119$ and $(c_+ - c_{10f_+})/(c_- - c_{010}) = 0.969$. For the MD simulations in 15m water-in-LiTFSI, $c_{10x}/c_{010} = 1.618$ and $(c_+ - c_{10x})/(c_- - c_{010}) = 0.893$. Quantitatively reflecting the bias we saw graphically in Fig. S1 for both the theory and the MD.

B. Short-range correlation parameter

Incorporating the α-parameter from Ref. 1 is straight-forward, as it arrives through the excess chemical potential. The resulting changes to the overall system of equations are

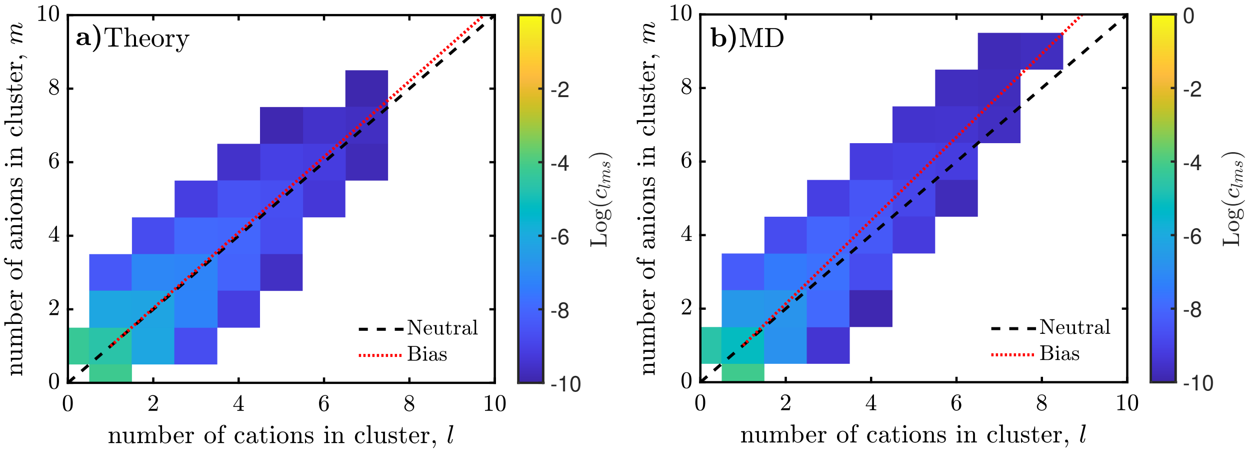

Figure S1. Bulk cluster distribution and multi-ion cluster charge bias. When the bias line is above the neutral line, it is negatively biased, and when it is below the neutral line, it is positively biased. a) Theory's prediction under the sticky-cation formalism for 15m WiSE. b) MD simulation's prediction for 15m water-in-LiTFSI. For the theory, we use $f_+ = 4$, $f_- = 3$, $\xi_0 = 1$, $\xi_+ = 0.4$, $\xi_- = 10.8$, $\epsilon_r = 10.1$, $\lambda = 0.231$, $P = 4.995$ Debye, and $v_0 = 22.5$ Å^3.

minor, only the general cluster chemical potential equation is shown here,

$$\begin{aligned}\beta\bar{\mu}_{lms} =& (l-m)\beta e\alpha\Phi - \ln\left(\frac{\mathrm{Sinh}(\beta P|\nabla\Phi|)}{\beta P|\nabla\Phi|}\right)\delta_{l,0}\delta_{m,0}\delta_{s,1} + 1 + \ln(\bar{\phi}_{lms}) + \beta\Delta_{lms} \\ &- (\xi_+ l + \xi_- m + s)\Lambda + (\xi_+ l + \xi_- m + s)\beta\bar{d}'\end{aligned} \tag{S8}$$

where $\bar{d}' = \bar{c}_+^{gel}\partial\bar{\Delta}_+^{gel} + \bar{c}_-^{gel}\partial\bar{\Delta}_-^{gel} + \bar{c}_0^{gel}\partial\bar{\Delta}_0^{gel}$, with the derivative being with respect to $\bar{\phi}_{lms}$. This modification can be carried through the subsequent steps to arrive at the modified system of equations. The only change to the system of equations will be $\alpha\Phi$ in place of Φ. Note this means $\nabla\Phi$ is unaffected for the fluctuating Langevin dipole terms. This extension can be directly incorporated into the general and sticky cation system of equations.

C. System of Equations

As discussed in the main text, to connect the bulk equations to quantities within the EDL and the modified Poisson-Boltzmann equation, we need to solve a system of equations. Generally, these equations consist of the Boltzmann closure relations, of which there are 3, described in the main text, which connect the free species in the bulk to the free species in the EDL, to the electrostatic potential and its derivative, in a consistent way. As we do

not restrict species to be the same size, we also enforce incompressibility, which is another equation that is introduced through a Lagrange multiplier and appears through $\tau = \exp(\Lambda)$. Note τ is implicitly dependent on the composition, Φ, and $\nabla\Phi$. This reliance leads to the cluster distribution function, $\bar{c}_{lms}$, to be implicitly dependent on $\nabla\Phi$. The implicit nature emerges since $\bar{\phi}_{001}$ and $\bar{\lambda}_{+0}$ are the only terms with an explicit $\nabla\Phi$ dependence in the cluster distribution function; however as they appear together as $\bar{\phi}_{001}\bar{\lambda}_{+0}$, this combination leads to the explicit dependence on $\nabla\Phi$ canceling out. A similar cancellation exists for the sticky-cation approximation form of the cluster distribution function. These free species concentrations are intrinsically linked to the conservation of associations and the mass action laws, both of which are also required to solve the EDL system of equations. To remove the dependence of the equations on the free species, and incorporate the conservation of associations and the mass action laws, we can substitute $\phi_{100} = \phi_+(1 - p_{+-} - p_{+0})^{f_+}$ [or $\phi_{10f_+} = \phi_+(1 + f_+/\xi_+)(1 - p_{+-})^{f_+}$ for the sticky case], $\phi_{010} = \phi_-(1 - p_{-+})^{f_-}$, and $\phi_{001} = \phi_0(1 - p_{0+})$. The conservation of associations and the mass action laws introduce another 4 equations. In total, we have 8 unknown variables and 8 equations, and therefore the system can be solved. For the non-sticky case, we have

$$\bar{\phi}_+ = \frac{\phi_{100}\exp(-e\beta\Phi)\tau^{\xi_+}}{(1 - \bar{p}_{+-} - \bar{p}_{+0})^{f_+}} = \frac{\xi_+}{f_+}\bar{\gamma}_{100}\tau^{\xi_+}\frac{1}{(1 - \bar{p}_{+-} - \bar{p}_{+0})^{f_+}} \tag{S9}$$

$$\bar{\phi}_- = \frac{\phi_{010}\exp(\beta e\Phi)\bar{\tau}^{\xi_-}}{(1 - \bar{p}_{-+})^{f_-}} = \frac{\xi_-}{f_-}\bar{\gamma}_{010}\tau^{\xi_-}\frac{1}{(1 - \bar{p}_{-+})^{f_-}} \tag{S10}$$

$$\bar{\phi}_0 = \frac{\mathrm{Sinh}(\beta eP|\nabla\Phi|)}{\beta eP|\nabla\Phi|}\frac{\phi_{001}\tau}{(1 - \bar{p}_{0+})} = \bar{\gamma}_{001}\tau\frac{1}{(1 - \bar{p}_{0+})} \tag{S11}$$

$$\bar{\phi}_+ + \bar{\phi}_- + \bar{\phi}_0 = 1 \tag{S12}$$

$$\frac{f_+\phi_+p_{+-}}{\xi_+} = \frac{f_-\phi_-p_{-+}}{\xi_-} = \zeta \tag{S13}$$

$$\frac{f_+\phi_+p_{+0}}{\xi_+} = \phi_0 p_{0+} = \Gamma \tag{S14}$$

$$\lambda_{+-}\zeta = \frac{p_{+-}p_{-+}}{(1 - p_{+-} - p_{+0})(1 - p_{-+})} \tag{S15}$$

$$\lambda_{+0}\Gamma = \frac{p_{+0}p_{0+}}{(1 - p_{+-} - p_{+0})(1 - p_{0+})} \tag{S16}$$

where $\bar{\gamma}_j$ has been introduced and can be considered the number of association sites per lattice site in the EDL *unadjusted by the Lagrange multiplier* for the bare cations (fully

hydrated cations), anions, and solvent species. For the sticky-cation formulation,

$$\bar{\phi}_+ = \frac{\phi_{10f_+}\exp(-\beta e\Phi)\tau^{(\xi_+ + f_+)}}{(1+f_+/\xi_+)\,\bar{p}_{+0}^{f_+}} = \frac{\xi_+ + f_+}{f_+}\bar{\gamma}_{10f_+}\tau^{\xi_+ + f_+}\frac{1}{(1+f_+/\xi_+)\,\bar{p}_{+0}^{f_+}} \tag{S17}$$

$$\bar{\phi}_- = \frac{\phi_{010}\exp(\beta e\Phi)\tau^{\xi_-}}{(1-\bar{p}_{-+})^{f_-}} = \frac{\xi_-}{f_-}\bar{\gamma}_{010}\tau^{\xi_-}\frac{1}{(1-\bar{p}_{-+})^{f_-}} \tag{S18}$$

$$\bar{\phi}_0 = \frac{\mathrm{Sinh}(\beta eP|\nabla\Phi|)}{\beta eP|\nabla\Phi|}\frac{\phi_{001}\tau}{(1-\bar{p}_{0+})} = \bar{\gamma}_{001}\tau\frac{1}{(1-\bar{p}_{0+})} \tag{S19}$$

$$\bar{\phi}_+ + \bar{\phi}_- + \bar{\phi}_0 = 1$$

$$p_{+-} = \frac{\psi_0 - \psi_+ + \lambda(\psi_+ + \psi_-) - \sqrt{(\lambda(\psi_- - \psi_+) + \psi_0 + \psi_+)^2 + 4(\lambda-1)\psi_0\psi_+}}{2(\lambda-1)\psi_+} \tag{S20}$$

$$p_{-+} = \frac{\psi_0 - \psi_+ + \lambda(\psi_+ + \psi_-) - \sqrt{(\lambda(\psi_- - \psi_+) + \psi_0 + \psi_+)^2 + 4(\lambda-1)\psi_0\psi_+}}{2(\lambda-1)\psi_-} \tag{S21}$$

$$p_{+0} = 1 - \frac{\psi_0 - \psi_+ + \lambda(\psi_+ + \psi_-)}{2(\lambda-1)\psi_+} + \frac{\sqrt{(\lambda(\psi_- - \psi_+) + \psi_0 + \psi_+)^2 + 4(\lambda-1)\psi_0\psi_+}}{2(\lambda-1)\psi_+} \tag{S22}$$

$$p_{0+} = \frac{\psi_+}{\psi_0} - \frac{\psi_0 - \psi_+ + \lambda(\psi_+ + \psi_-)}{2(\lambda-1)\psi_0} + \frac{\sqrt{(\lambda(\psi_- - \psi_+) + \psi_0 + \psi_+)^2 + 4(\lambda-1)\psi_0\psi_+}}{2(\lambda-1)\psi_0} \tag{S23}$$

Where the last four equations come from the conservation of associations, the law of mass action on the number of associations, and the sticky cation approximation $p_{+-} + p_{+0} = 1$, we can obtain explicit expressions of our association probabilities in terms of the ψ_i and λ. Additionally, in both sets of equations, the last five equations hold in a nontrivial fashion for both the bulk and EDL quantities.

The above system of equations tends to be the clearest way to understand the underlying physics of the system and, hence, its predictions. However, one can reduce these systems of 8 equations down to a system of 2 equations, which can provide some insight into the system of equations being solved and an alternative way of obtaining the roots. This reduction of equations was motivated by and achieved through writing the unknowns in terms of the association probabilities. Initially, we will derive the relationship for the general case followed by the sticky cation approximation. For brevity, the application of algebraic manipulations of $\bar{\lambda}$ is utilized implicitly:

$$\bar{\lambda} = \frac{\bar{p}_{-+}(1-\bar{p}_{0+})}{\bar{p}_{0+}(1-\bar{p}_{-+})}. \tag{S24}$$

In order to derive these equations, we construct the Boltzmann closure relationships between the bare anions and the free solvent and between the bare cations and the free

solvent. These kinds of relationships were first introduced in Ref. 2,

$$\frac{\bar{\phi}_-(1-\bar{p}_{-+})^{f_-}}{\bar{\phi}_0(1-\bar{p}_{0+})} = \frac{\xi_-\bar{\gamma}_{010}}{f_-\bar{\gamma}_{001}}\tau^{\xi_- -1}, \tag{S25}$$

$$\frac{\bar{\phi}_+(1-\bar{p}_{+-}-\bar{p}_{+0})^{f_+}}{\bar{\phi}_0(1-\bar{p}_{0+})} = \frac{\xi_+\bar{\gamma}_{100}}{f_+\bar{\gamma}_{001}}\tau^{\xi_+ -1}. \tag{S26}$$

These expressions can be further reduced to be explicitly in terms of probabilities, the compressibility constraint, and $\bar{\gamma}_i$'s through using the conservation of association equations, Eq. (S13) & (S14), to convert the volume ratios into probability ratios. The idea of this is to utilize substitutions and transformations where possible to reduce the number of unknowns and equations needed to be solved. Note this idea will be used multiple times to simplify our system of equations. Applying this procedure and using Eq. (S24) to simplify our equations we find:

$$\frac{1}{\bar{\lambda}}\frac{\bar{p}_{+-}}{\bar{p}_{+0}}(1-\bar{p}_{-+})^{f_- -1} = \frac{\bar{\gamma}_{010}}{\bar{\gamma}_{001}}\tau^{\xi_- -1}, \tag{S27}$$

$$\frac{\bar{p}_{0+}}{1-\bar{p}_{0+}}\frac{(1-\bar{p}_{+-}-\bar{p}_{+0})^{f_+}}{\bar{p}_{+0}} = \frac{\bar{\gamma}_{100}}{\bar{\gamma}_{001}}\tau^{\xi_+ -1}. \tag{S28}$$

To further reduce these equations, one can note that the formulas for $\bar{p}_{ij}$ in terms of $\bar{\eta} = (1-\bar{p}_{+-}-\bar{p}_{+0})$ (the probability a cation association site is empty), τ, and $\bar{\gamma}_i$ can all be written down in terms of the two unknowns $\bar{\eta}$ and τ. Thus, as we will show this allows one to collapse the 8 equations into 2 equations. This will be accomplished through a series of substitutions where we show all the probabilities can be written in terms of $\bar{\eta}$ and τ. This finding is sufficient to show the reduction to 2 equations as we already know the volume fractions can be written in terms of the probabilities and τ, i.e. 8 equations to 5 immediately and with these manipulations to 2. One can start by substituting the cation form of ζ from Eq. (S13) into Eq. (S15) and using Eq. (S9) for $\bar{\phi}_+$ as well as algebraic manipulation,

$$\bar{p}_{0+} = \frac{\lambda_{+-}\bar{\gamma}_{100}\tau^{\xi_+}}{\bar{\lambda}\bar{\eta}^{f_+ -1}+\lambda_{+-}\bar{\gamma}_{100}\tau^{\xi_+}}, \tag{S29}$$

which by conversion through the algebraic manipulations of $\bar{\lambda}$ becomes,

$$\bar{p}_{-+} = \frac{\lambda_{+-}\bar{\gamma}_{100}\tau^{\xi_+}}{\bar{\eta}^{f_+ -1}+\lambda_{+-}\bar{\gamma}_{100}\tau^{\xi_+}}. \tag{S30}$$

By substituting Eq. (S29) into Eq. (S28) we obtain,

$$\bar{p}_{+0} = \bar{\lambda}_{+0}\bar{\gamma}_{001}\bar{\eta}\tau, \tag{S31}$$

which by the definition of $\bar{\eta}$ means,

$$\bar{p}_{+-} = 1 - \left(1 + \bar{\lambda}_{+0}\bar{\gamma}_{001}\tau\right)\bar{\eta}. \tag{S32}$$

We produce our new polynomial in $\bar{\eta}$ whose roots contains the solutions by substituting Eq. (S30)-(S32) into Eq. (S27),

$$\left(1 + \lambda_{+-}\bar{\gamma}_{010}\tau^{\xi_-} + \bar{\lambda}_{+0}\bar{\gamma}_{001}\tau\right)\bar{\eta}^{(f_+-1)(f_--1)+1} - \bar{\eta}^{(f_+-1)(f_--1)} + \bar{\eta}\sum_{k=0}^{f_--2}\binom{f_- - 1}{k}\left(\lambda_{+-}\bar{\gamma}_{100}\tau^{\xi_+}\right)^{(f_--1)-k}\bar{\eta}^{k(f_+-1)} = 0. \tag{S33}$$

Lastly, an analogous equation for the incompressibility constraint is required; here, $\bar{p}_{ij}$ will kept as functions of $\bar{\eta}$ and τ for simplicity. This is accomplished by substituting Eq. (S9)-(S11) into Eq. (S12),

$$\frac{\xi_+}{f_+}\bar{\gamma}_{100}\tau^{\xi_+}\frac{1}{(1-\bar{p}_{+-}-\bar{p}_{+0})^{f_+}} + \frac{\xi_-}{f_-}\bar{\gamma}_{010}\tau^{\xi_-}\frac{1}{(1-\bar{p}_{-+})^{f_-}} + \bar{\gamma}_{001}\tau\frac{1}{(1-\bar{p}_{0+})} = 1. \tag{S34}$$

Hence, Eqs. (S33)-(S34), can be solved in their reduced form for $\bar{\eta}$ and τ, which uniquely determine the composition of the WiSE in the EDL. It's important to note the unique property belonging to Eq. (S33) as it is a polynomial, which means it will have $(f_+ - 1)(f_- - 1) + 1$ roots for a given τ. This information can be further refined by noting for $\bar{\eta}$ to be meaningful that it must take on a value between zero and one inclusively. Thus by utilizing Descartes' rule of sign [3] and noting that any valid τ must be a finite positive value, one can prove that for $f_+, f_- > 2$, this polynomial will have at most two positive roots and has at least one zero root which appears to corresponds to sticky cation approximation's case. The zeros correspond to the sticky cation approximation as that solution corresponds to singularities that are introduced from $\eta = 0$, which alternatively can be written as $\bar{p}_{+-} + \bar{p}_{+0} = 1$ which is the sticky cation approximation. Therefore when zero roots occur, one needs to use the sticky cation version of the polynomial to account for the singularity in the equations introduced with the zero root.

For the lower functionality cases, it's more case-specific: (1) when either $f_+ = 1$ or $f_- = 1$ there is one positive root, (2) when $f_+ = 2$ and $f_- > 2$, there are at most 2 positive roots and at least one zero root, (3) when $f_+ = f_- = 2$ there is one positive root and one zero root, and (4) when $f_- = 2$ and $f_+ > 2$ there are at most 2 positive roots and at least 1 zero root. It is important to note that the negative coefficient term in this polynomial will

remain negative outside of limiting cases where it goes to zero. Additionally when $f_+ = 2$ and $f_- > 1$, the coefficient that is subtracted from one in the summation leads to a strictly negative net coefficient in the non-limiting or non-enforced sticky cases. We obtain this conclusion as for the coefficient to greater or equal to zero, then (1-$\bar{p}_{-+})^{f_- -2}\bar{p}_{+-}\bar{p}_{-+}$-1 ≥ 0. This exact result also holds for the stick cation case, but only here does the limiting cases have the capabilities to satisfy the previous inequality.

This analysis can be repeated for the sticky cation approximation. The approximation dictates that $\bar{p}_{+-} = 1 - \bar{p}_{+0}$, using this expression helps to reduce the complexity of the intermediate expressions. To derive the reduced equations here, one must construct the Boltzmann closure relationships. In this case, the expressions are between 1) the bare anions and the free solvent and 2) between the fully hydrated cations and the free solvent.

$$\frac{\bar{\phi}_-(1-\bar{p}_{-+})^{f_-}}{\bar{\phi}_0(1-\bar{p}_{0+})} = \frac{\xi_- \bar{\gamma}_{010}}{f_- \bar{\gamma}_{001}} \tau^{\xi_- -1}, \tag{S35}$$

$$\frac{\bar{\phi}_+ \bar{p}_{+0}^{f_+}}{\bar{\phi}_0(1-\bar{p}_{0+})} = \frac{\xi_+ \bar{\gamma}_{10f_+}}{f_+ \bar{\gamma}_{001}} \tau^{\xi_+ + f_+ -1}. \tag{S36}$$

These expressions can be further reduced to be explicitly in terms of probabilities, the compressibility constraint, and $\bar{\gamma}_i$'s through using the conservation of association equations, Eq. (S13) & (S14), to convert the volume ratios into probability ratios. Here we also invoke $\bar{p}_{+-} = 1 - \bar{p}_{+0}$. This derivation follows a similar style as the general formalism. Applying the procedure and using Eq. (S24) to simplify our equations we find:

$$\frac{1}{\bar{\lambda}} \frac{1-\bar{p}_{+0}}{\bar{p}_{+0}} (1-\bar{p}_{-+})^{f_- -1} = \frac{\bar{\gamma}_{010}}{\bar{\gamma}_{001}} \tau^{\xi_- -1}, \tag{S37}$$

$$\frac{\bar{p}_{0+}}{1-\bar{p}_{0+}} \bar{p}_{+0}^{f_+ -1} = \frac{\bar{\gamma}_{10f_+}}{\bar{\gamma}_{001}} \tau^{\xi_+ + f_+ -1}. \tag{S38}$$

To reduce this system of equations, one can note that formulating $\bar{p}_{ij}$ in terms of $\bar{p}_{+0}$, τ, and our $\bar{\gamma}_i$ can collapse the system from 8 to 2 equations and unknowns. This procedure is accomplished in a more direct fashion but similar style to the general case, as Eq. (S38) can be directly manipulated to give an equation for $\bar{p}_{0+}$ in terms of $\bar{p}_{+0}$, τ, and our $\bar{\gamma}_i$'s,

$$\bar{p}_{0+} = \frac{\bar{\gamma}_{10f_+} \tau^{\xi_+ + f_+ -1}}{\bar{\gamma}_{001} \bar{p}_{+0}^{f_+ -1} + \bar{\gamma}_{10f_+} \tau^{\xi_+ + f_+ -1}}, \tag{S39}$$

which by conversion through the algebraic manipulations of $\bar{\lambda}$ produces a formula for $\bar{p}_{-+}$,

$$\bar{p}_{-+} = \frac{\bar{\lambda} \bar{\gamma}_{10f_+} \tau^{\xi_+ + f_+ -1}}{\bar{\gamma}_{001} \bar{p}_{+0}^{f_+ -1} + \bar{\lambda} \bar{\gamma}_{10f_+} \tau^{\xi_+ + f_+ -1}}. \tag{S40}$$

By substituting Eq. (S40) into Eq. (S37) we obtain,

$$\frac{1}{\bar{\lambda}}\frac{1-\bar{p}_{+0}}{\bar{p}_{+0}}\left(\frac{\bar{\gamma}_{001}\bar{p}_{+0}^{f_+-1}}{\bar{\gamma}_{001}\bar{p}_{+0}^{f_+-1}+\bar{\lambda}\bar{\gamma}_{10f_+}\tau^{\xi_++f_+-1}}\right)^{f_--1}=\frac{\bar{\gamma}_{010}}{\bar{\gamma}_{001}}\tau^{\xi_--1}. \tag{S41}$$

This can be algebraically manipulated to produce our new polynomial whose roots are the solutions to $\bar{p}_{+0}$,

$$\begin{aligned}(\bar{\lambda}\bar{\gamma}_{010}\tau^{\xi_-}+&\bar{\gamma}_{001}\tau)(\bar{\gamma}_{001}\tau)^{f_--1}\bar{p}_{+0}^{(f_+-1)(f_--1)+1}-\bar{\gamma}_{001}^{f_-}\tau^{f_-}\bar{p}_{+0}^{(f_+-1)(f_--1)}\\&+\bar{\lambda}\bar{\gamma}_{010}\tau^{\xi_-}\bar{p}_{+0}\sum_{k=0}^{f_--2}\binom{f_--1}{k}(\bar{\gamma}_{001}\tau)^k(\bar{\lambda}\bar{\gamma}_{10f_+}\tau^{\xi_++f_+})^{(f_--1)-k}\bar{p}_{+0}^{k(f_+-1)}=0.\end{aligned} \tag{S42}$$

Lastly, an analogous equation enforcing incompressibility must be constructed keeping $\bar{p}_{ij}$ as functions of $\bar{p}_{+0}$ and τ for simplicity. This is accomplished by substituting Eq. (S17)-(S19) into Eq. (S12),

$$\frac{\xi_+}{f_+}\bar{\gamma}_{10f_+}\tau^{\xi_++f_+}\frac{1}{\bar{p}_{+0}^{f_+}}+\frac{\xi_-}{f_-}\bar{\gamma}_{010}\tau^{\xi_-}\frac{1}{(1-\bar{p}_{-+})^{f_-}}+\bar{\gamma}_{001}\tau\frac{1}{(1-\bar{p}_{0+})}=1. \tag{S43}$$

Here Eq. (S42)-(S43) can be solved in their reduced form for the $\bar{p}_{+0}$ and τ which uniquely determines the composition of the WiSE in the EDL. This new polynomial for $\bar{p}_{+0}$, Eq. (S42) brings with it the useful properties discussed earlier which can aid in solving and understanding WiSEs.

For this and the prior case, the solution roots can be found, and each is tested to find a valid solution. Here to support computational efficiency, this was done by using the previous root solutions as the root to test as the valid solution. For the work presented here, this closest root on a sufficiently fine grid produced a valid solution. Therefore, the other potentially valid solutions were neglected. Most importantly, the predictions from this reduced system were tested in all of the previous equations to validate sufficient convergence against the initial system of equations.

D. EDL Calculations

In order to solve the sticky cation system of equations with the modified Poisson-Boltzmann (PB) equation, we utilize the following procedure. Initially, we calculate the bulk properties using Eqs. (S20)-(S23) with the cation association ratio. In Section I C, the system of 8 equations, Eqs. (S17)-(S23), needs to be solved to obtain the 8 unknowns, i.e.,

the volume fractions, association probabilities and the Lagrange multiplier. Therefore, we obtain the relationships between $\bar{\phi}_+, \bar{\phi}_-, \bar{\phi}_{001}$ and Φ,$\nabla\Phi$, on a regular grid of Φ & $\nabla\Phi$, which can then be used to solve the modified PB equation. For quicker computational, we created additional mappings of Φ & $\nabla\Phi$ to all the unknown variables and composite variables, such as ρ_e & ϵ, to solve the modified PB equation numerically. This procedure is similar to that of Ref. 4, but with 2D maps depending on Φ & $\nabla\Phi$.

Similarly for the non-sticky system of equations with the modified PB equation, we can utilize an analogous procedure. Here, we calculate the bulk properties using Eqs. (S13)-(S16) with the association constants. Again, in Section I C, the system of 8 equations, Eqn. (S9)-(S16), are stated which need to be solved to obtain the 8 unknowns Once again one can establish mappings between $\bar{\phi}_+, \bar{\phi}_-, \bar{\phi}_{001}$ and Φ,$\nabla\Phi$, on a regular grid of Φ & $\nabla\Phi$, which can then be used to solve the modified PB equation. Additional mappings of Φ & $\nabla\Phi$ to all the unknown variables and composite variables, such as ρ_e & ϵ, were conducted to simplify numerically solving the modified PB equation numerically.

To solve for various profiles in the EDL as well as the screening length, differential capacitance, excess surface concentrations, and interfacial concentration of water, we use the following steps similar to the methodology utilized in Ref. 2 & 4:

1. First, we numerically solve the system of equations in this work. The polynomial formulation was used for $\bar{\phi}_+$, $\bar{\phi}_-$, $\bar{\phi}_0$, dimensionless ρ_e, $\bar{p}_{+-}$, $\bar{p}_{+0}$, $\bar{p}_{-+}$, $\bar{p}_{0+}$, τ, and dimensionless ϵ over a range of electrostatic potential and electric field strength values. This was done for a grid of dimensionless electrostatic potential ($\Phi e\beta$) and dimensionless electric field strength ($e\beta\lambda_D\nabla\Phi$) to create a refined mesh; for our purposes here, a spacing of 0.01 was used. After solving the WiSE at a set composition and λ, the refined maps for $\bar{\phi}_+$, $\bar{\phi}_-$, $\bar{\phi}_0$, dimensionless ρ_e, $\bar{p}_{+-}$, $\bar{p}_{+0}$, $\bar{p}_{-+}$, $\bar{p}_{0+}$, τ, and ϵ were saved, allowing one to interpolated solution for these quantities from these maps for a given electrostatic potential and electric field strength. Sample maps are shown at the end of this section in Fig. S2-S4.

2. Using the dimensionless ρ_e and dimensionless ϵ maps (shown in (Fig. S4), we can then numerically solve the modified PB equation to get a solution for the electrostatic potential and electric field profile in the EDL. Our boundary condition for a charge

surface is,

$$(\epsilon \nabla \Phi)\big|_s = -q_s \mathbf{n}. \tag{S44}$$

The boundary condition for the bulk is,

$$\Phi(\mathbf{r} \to \infty) = 0. \tag{S45}$$

3. The electrostatic potential and electric field profile in the EDL along with our interpolation maps allows us to predict profiles of the various quantities of interest in the EDL: dimensionless charge density, total volume fractions of each species, volume fraction of free (or fully hydrated) cations, volume fraction free anions, volume fraction of free water, volume fraction aggregates as well as individual clusters volume fractions, association probabilities, and the product of the ionic association probabilities. Lastly from the individual clusters' volume fractions, one can convert into the dimensionless cluster concentrations and, hence, numerically evaluate the simplified form of Eq. (S50) till the evaluation converges to determine the length scale of the aggregates. Here, we summed over all valid Sticky Cation Cayley tree clusters containing up to 100 cations and up to 100 anions. Additionally, using the individual clusters' volume fractions, one can obtain the dimensionless concentrations, which allows one to create the cluster distribution plots for $l + m > 0$.

4. To determine the screening length (λ_s), we applied a ± 0.001 V electrostatic potential boundary condition at the surface. We obtained the screening length from the electrostatic potential profile in the EDL by fitting an exponential decay to the profile and extracting the exponential decay constant for a range of molalities as shown in Fig. S9. In Fig. S9, it was constructed using a 0.001 V electrostatic potential boundary condition. It is worth noting that the screening lengths obtained by ± 0.001 V solutions are very similar. Additionally, these profiles are within the pre-gel regime for the screening length plot in Fig. S9. As λ_s is plotted against molality and the system's MD parameters fluctuate slightly with the molality, the average value of v_0 and λ was used to generate this figure. Lastly, to determine the bulk WiSE composition, we utilized the following formula to determine the ϕ_+ from the molality (m) and the molar mass of the solvent (M_s), as from bulk electroneutrality and incompressibility

this fully determines the bulk composition:

$$\phi_+ = \left(1 + \frac{1}{\xi_+ \mathrm{M}_s \mathrm{m}} + \frac{\xi_-}{\xi_+}\right)^{-1} \tag{S46}$$

5. To obtain our differential capacitance predictions, we introduce the α-parameter and set it to 0.1 to bring the voltage range of the profile more in line with real-world systems. Following this and similar to previous steps, we solved the new system to obtain solution maps to the modified system of equations. From these maps, one could solve the modified PB equation. Then, we solved for the potential at the interface (Φ_s) over a range of surface charge densities (q_s) for our boundary conditions, here we used a fine grid spacing of approximately 0.0001 $\mathrm{C/m^2}$. Note the solution to the system impacts the value of the dimensional surface charge density; hence, this spacing for q_s was found numerically. From this map, we next constructed splines to calculate how q_s depends on Φ_s. Using these splines, we calculated the differential capacitance by numerically taking the derivative of q_s with respect to Φ_s using finite differences.

6. The excess surface concentrations can be obtained in our current theory via numerical integration of the difference between the species' concentration and its bulk concentration. Note to obtain the concentration, one can convert the volume fractions to the dimensionless concentration as $c_i = \phi_i/\xi_i$ then divide by v_0 to obtain the concentration. In this work, the numerical integration was conducted on the fine spacing produced by the numerical solver for the boundary value problem.

7. Obtaining the interfacial concentration of water is more intricate as the depletion region is important for correctly evaluating this quantity. Hence to make the predicted values from the theory more analogous to the predictions from the MD simulations, the depletion region found for the MD cases was used to shift the theory's dimensionless concentration before integration. As this region has zero value, one can reduce the upper integration bound accordingly, which was done here. Note for the zero charge surface, the depletion region was not symmetric; hence, the average value was used. With the properly shifted dimensionless concentration of water profiles, which are obtained by converting from volume fraction, one can numerically integrate these curves up from 0 to ℓ_w (here 5 Å) and divide by ℓ_w as well as the bulk value of total water. Additionally, as one needs to have it end at a specific distance, the water curves

were used to construct splines, which can be numerically integrated over the specific domain with a refined spacing.

As just discussed earlier, the first step towards obtaining our theory's predictions is to generate numerical solutions to the system of equations. Examples of these maps for 15m water-in-LiTFSI under the sticky-cation formalism are shown below in Fig. S2-S4, along with Fig. S5, which highlights how close to the gelation the system is where negative implies gelation has occurred.

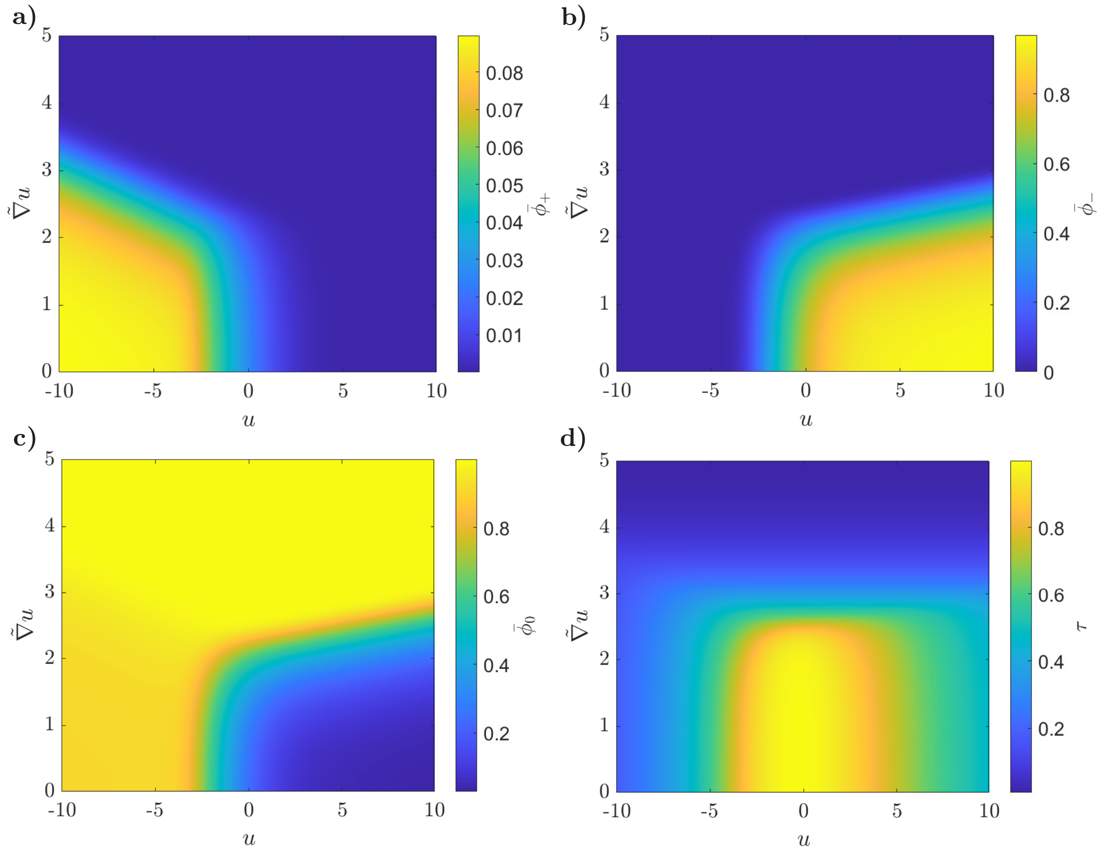

Figure S2. Numerical solution map for the species volume fractions ($\bar{\phi}_i$) and exponential of the Lagrange multiplier (τ) in our system of equations under the sticky-cation formalism for 15m WiSE. a) Volume fraction of cations ($\bar{\phi}_+$). b) Volume fraction of anions ($\bar{\phi}_-$). c) Volume fraction of solvent ($\bar{\phi}_0$). d) Exponential of the Lagrange multiplier (τ). Shown here is how these unknowns vary with the dimensionless electrostatic potential (u) and dimensionless electric field strength ($\tilde{\nabla}u$). Here we use $f_+ = 4$, $f_- = 3$, $\xi_0 = 1$, $\xi_+ = 0.4$, $\xi_- = 10.8$, $\epsilon_r = 10.1$, $\lambda = 0.231$, $P = 4.995$ Debye, and $v_0 = 22.5$ Å^3.

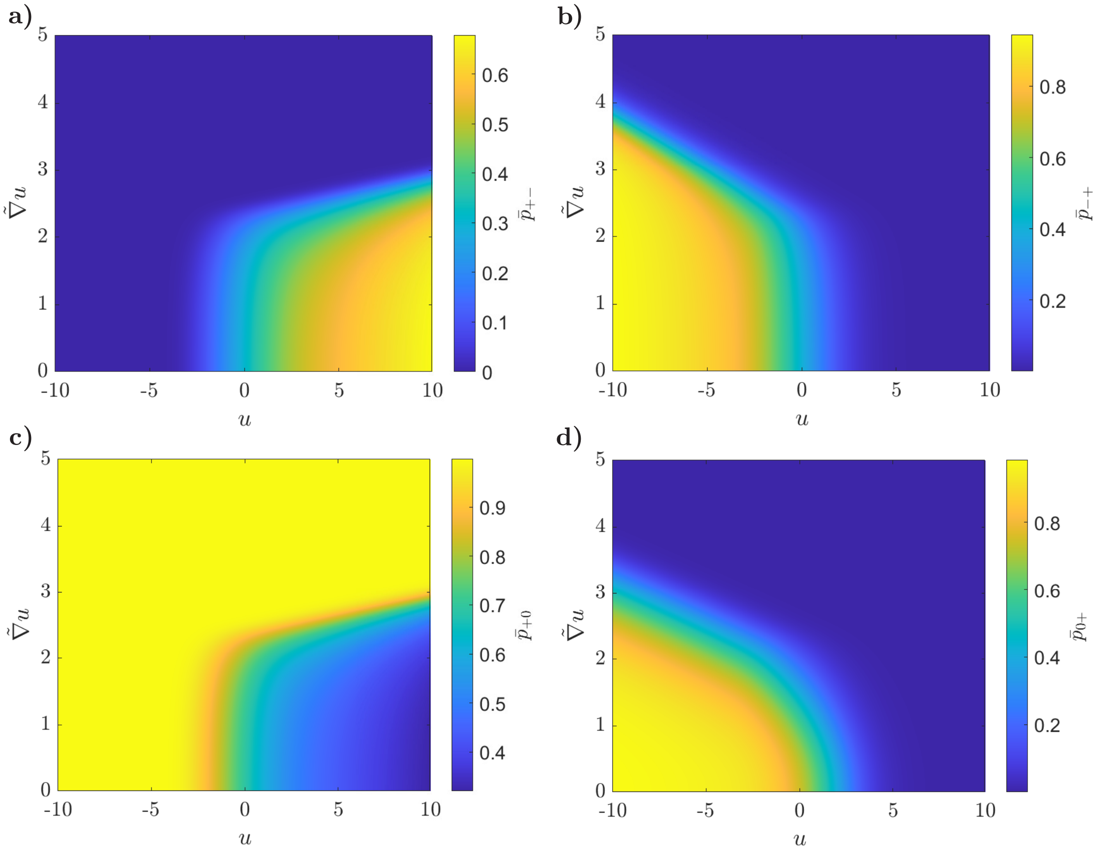

Figure S3. Numerical solution map for the association probabilities ($\bar{p}_{ij}$) in our system of equations under the sticky-cation formalism for 15m WiSE. a) Association probability of cations being bound to anion ($\bar{p}_{+-}$). b) Association probability of anions being bound to cation ($\bar{p}_{-+}$). c) Association probability of cations being bound to water ($\bar{p}_{+0}$). d) Association probability of water being bound to cations ($\bar{p}_{0+}$). Shown here is how the association probabilities vary with the dimensionless electrostatic potential (u) and dimensionless electric field strength ($\tilde{\nabla}u$). Here we use $f_+ = 4$, $f_- = 3$, $\xi_0 = 1$, $\xi_+ = 0.4$, $\xi_- = 10.8$, $\epsilon_r = 10.1$, $\lambda = 0.231$, $P = 4.995$ Debye, and $v_0 = 22.5$ Å^3.

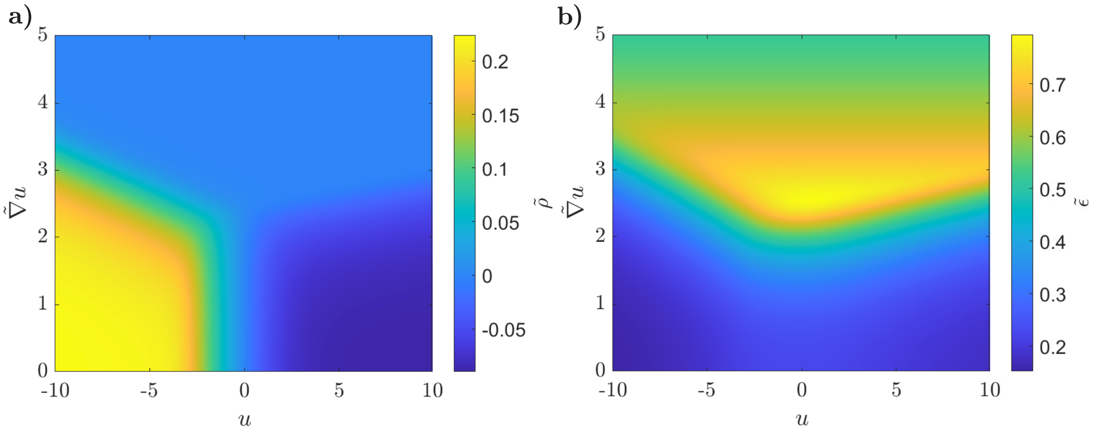

Figure S4. Numerical solution map for dimensionless charge density ($\tilde{\rho}$) and dimensionless dielectric constant ($\tilde{\epsilon}$) in our system of equations under the sticky-cation formalism for 15m WiSE. a) Dimensionless charge density ($\tilde{\rho}$). b) Dimensionless dielectric constant ($\tilde{\epsilon}$). Shown here is how these key unknowns for solving the modified-PB equation vary with the dimensionless electrostatic potential (u) and dimensionless electric field strength ($\tilde{\nabla}u$). Here we use $f_+ = 4$, $f_- = 3$, $\xi_0 = 1$, $\xi_+ = 0.4$, $\xi_- = 10.8$, $\epsilon_r = 10.1$, $\lambda = 0.231$, $P = 4.995$ Debye, and $v_0 = 22.5$ Å^3.

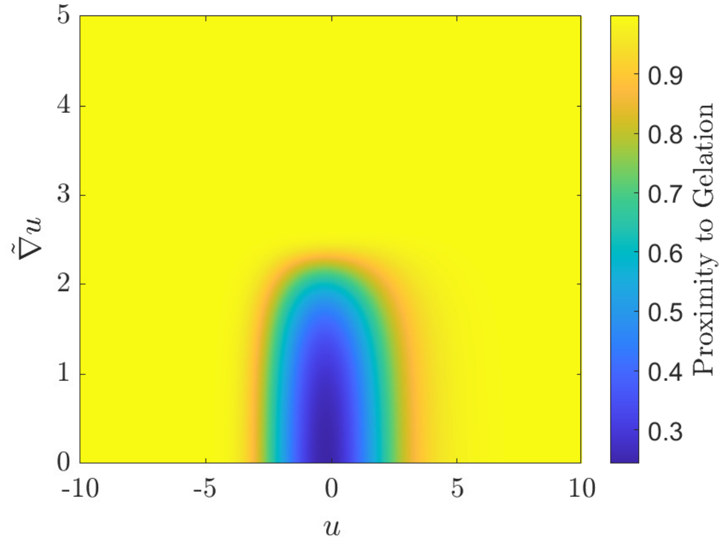

Figure S5. Numerical solution map for $1-(f_+-1)(f_--1)\bar{p}_{+-}\bar{p}_{-+}$ in our system of equations under the sticky-cation formalism for 15m WiSE. Shown here is how the proximity to gelation varies with the dimensionless electrostatic potential (u) and the negative dimensionless electric field strength ($\tilde{\nabla}u$). Here we use $f_+ = 4$, $f_- = 3$, $\xi_0 = 1$, $\xi_+ = 0.4$, $\xi_- = 10.8$, $\epsilon_r = 10.1$, $\lambda = 0.231$, $P = 4.995$ Debye, and $v_0 = 22.5$ Å^3.

E. Additional Theory Predictions

In the main text, we highlighted the ability of our theory to predict the increase in aggregation due to electric field-induced enhancement in associations. Shown below in Fig. S6 is a magnified view of this enhancement in 15m water-in-LiTFSI under the sticky cation approximation near a negatively charged electrode.

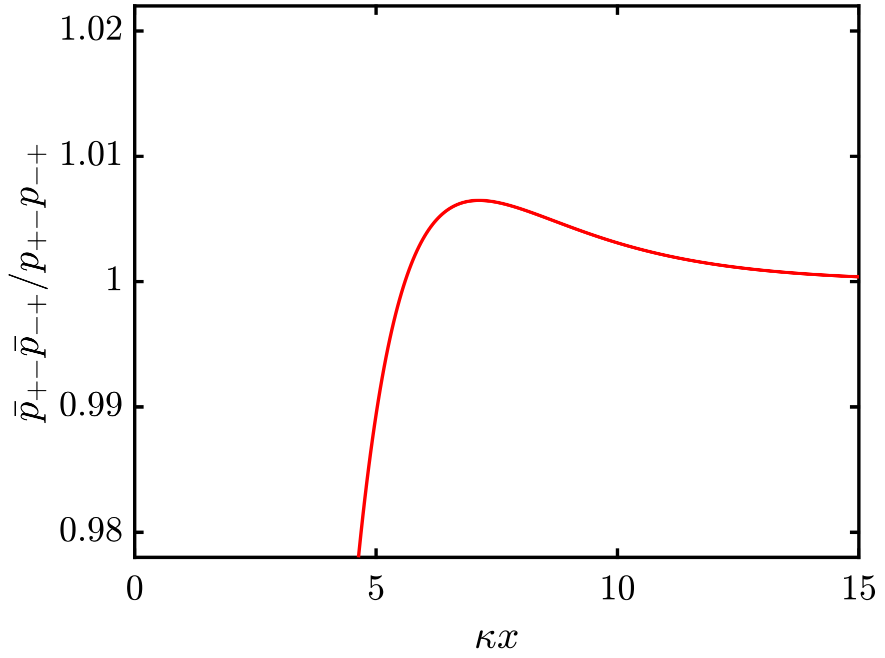

Figure S6. Electric field induced enhancement in associations in 15m WiSE. Shown is the product of the ionic association probabilities, $\bar{p}_{+-}\bar{p}_{-+}$, normalized by its bulk value $p_{+-}p_{-+}$. This figure is plotted only close to the charged surface to highlight the effect but was solved over the same regime as the figures comparing the MD simulation's spatial EDL profiles to the theory's predictions in the main text. Here we use $f_+ = 4$, $f_- = 3$, $\xi_0 = 1$, $\xi_+ = 0.4$, $\xi_- = 10.8$, $\epsilon_r = 10.1$, $\lambda = 0.231$, P = 4.995 Debye, $v_0 = 22.5$ Å^3, and q$_s$ = -0.2 C/m^2.

Next, let us discuss how the local association constant ($\bar{\lambda}$) varies through the EDL. As highlighted in the main text, the general trends in the $\bar{\lambda}$'s spatial profiles are consistent between the MD and the theory, with even the magnitudes agreeing to a reasonable degree. This result is shown for 15m LiTFSI at a surface charge density of $\mp$0.2 C/m^2 in Fig. S7. This agreement further supports the utility of our theory. Two main deviations between the theory and MD results can be seen in Fig. S7. First, at the condensed layer, the MD appears to diverge or become undefined. This deviation is expected given the structure of

the condensed layer, as co-ion concentrations vanish which leads to ill-defined association probabilities. Second, there are oscillations in the MD's $\bar{\lambda}$ not seen in the theory's prediction. Once again, this deviation is expected as the theory is a local formulation limiting the model from capturing oscillations in $\bar{\lambda}$ that are likely caused by electric field oscillations or species layering. Nonetheless, the model's ability to capture the trends in $\bar{\lambda}$'s spatial profile and order of magnitude is a promising result.

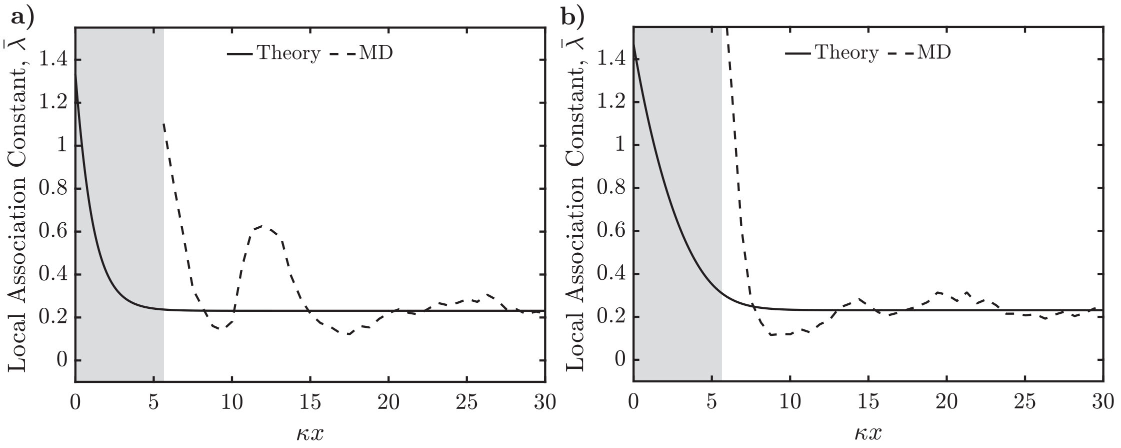

Figure S7. Local association constant ($\bar{\lambda}$) through the EDL in WiSEs. a) $\bar{\lambda}$ of 15m WiSE at q_s = -0.2 C/m^2 as a function of distance from the interface in dimensionless units, where κ is the inverse Debye length. b) $\bar{\lambda}$ of 15m WiSE at q_s = 0.2 C/m^2 as a function of distance from the interface in dimensionless units. Here we use $f_+ = 4$, $f_- = 3$, $\xi_0 = 1$, $\xi_+ = 0.4$, $\xi_- = 10.8$, $\epsilon_r = 10.1$, $\lambda = 0.231$, $P = 4.995$ Debye, and $v_0 = 22.5$ Å^3.

One can embed theory results like the differential capacitance into constructed meshes such as those shown in Fig. S2-S5 gaining insight into the system. For example in Fig. S8, the embedding provides insight into the factors that give rise to the local peaks in the differential capacitance profile. This embedding can be done with all the meshes for results with the same parametrization and for results that depend on the electrostatic profile. In this case, we focus on how the non-dimensional charge density (Fig. S8.a)) and dielectric constant (Fig. S8.b)) vary throughout the differential capacitance profile. First consider the peak at moderate negative potential, it occurs in a region of cation enrichment and has some dielectric enhancement that leads to the large peak we see at the same location in the differential capacitance. Second consider the peak at moderate positive potential, it occurs in a region of anion enrichment but with less significant dielectric enhancement leading to

the smaller peak we see at this same location in the differential capacitance. Third consider the peak at large positive potential, it occurs in a region of decreasing non-dimensional charge density and strongly increasing non-dimensional dielectric constant suggesting that this is a region of free water enrichment with significant dielectric enhancement. Additionally, these observed features can be further supported by considering other meshes similar to those shown in Fig. S2-S4. One can also utilize these meshes by constructing additional local quantity maps from these fundamental ones. For example, one could construct maps of the concentration of free water or ion pairs to provide additional insight into these systems. Moreover, one could also use these new meshes to develop intuition on key features in predicted profiles. This concept is demonstrated here as we embedded the differential capacitance profile into the non-dimensional charge density (Fig. S8.a)) and dielectric constant (Fig. S8.b)) meshes.

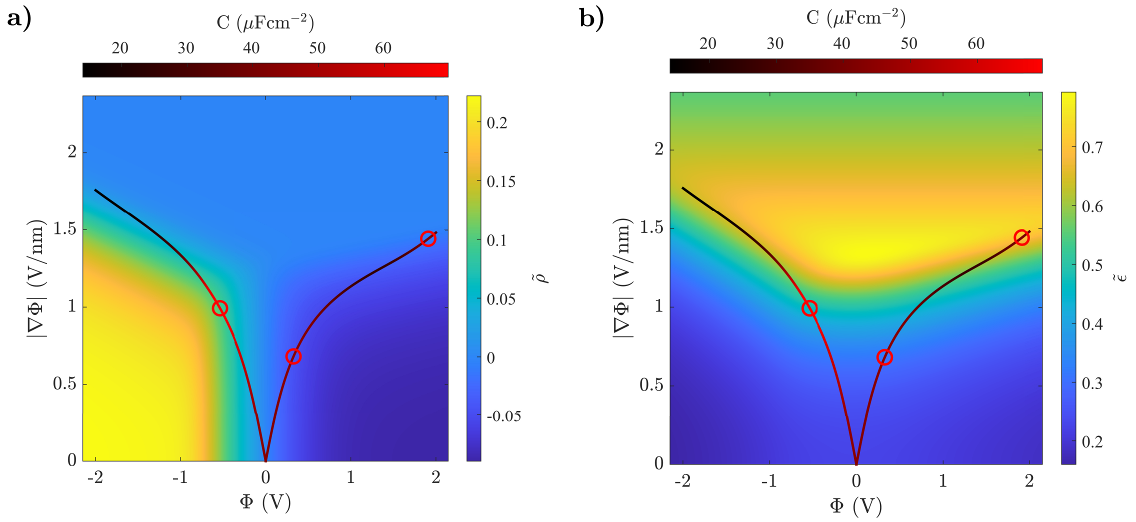

Figure S8. Differential capacitance profile (C) embedded into dimensionless charge density ($\tilde{\rho}$) and dimensionless dielectric constant ($\tilde{\epsilon}$) meshes for WiSE. This projection was constructed for 15m WiSE using the sticky-cation formalism. Local maximums in the differential capacitance profiles are marked by a red circle: a) Differential capacitance profile embedded in the dimensionless charge density ($\tilde{\rho}$). b) Differential capacitance profile embedded in the dimensionless dielectric constant ($\tilde{\epsilon}$). The maps are shown for variations in the electrostatic potential (Φ) and electric field strength ($\nabla\Phi$). Here we use $f_+ = 4$, $f_- = 3$, $\xi_0 = 1$, $\xi_+ = 0.4$, $\xi_- = 10.8$, $\epsilon_r = 10.1$, $\lambda = 0.231$, $P = 4.995$ Debye, $v_0 = 22.5$ Å^3, and $\alpha = 0.1$.

Now turning to the screening length, λ_s, varies with the molality in the pre-gel regime. In

Fig. S9, we can observe that the screening length decrease as the molality of LiTFSI increases up to 9 m, after which it is relatively constant. The enhancement of the screening length due to the associations can be seen in the inset of Fig. S9 where λ_s/λ_D is always greater than, or equal to, one. Additionally, we can observe around 9m that the contributions to the screening length from the associations begin to increase, leading to the screening length increasing more strongly with concentration.

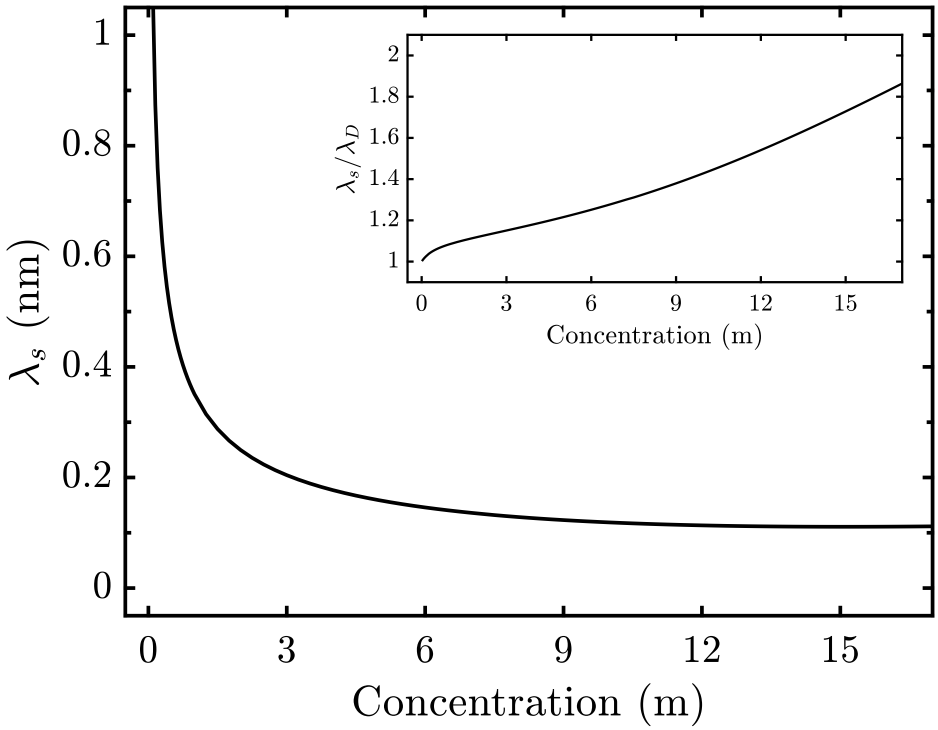

Figure S9. Screening length prediction for WiSEs. Screening length of WiSE as a function of molality. The inset shows the screening length normalized by the formal Debye length, $\lambda_D = \sqrt{v_0\epsilon/e^2\beta(c_+ + c_-)}$, as a function of molality. Here we use $f_+ = 4$, $f_- = 3$, $\xi_0 = 1$, $\xi_+ = 0.4$, $\xi_- = 10.8$, $\epsilon_r = 10.1$, $\lambda = 0.228$, $P = 4.995$ Debye, and $v_0 = 22.7$ Å^3.

II. EXTENDED SIMULATION SECTION

Presented here is an in-depth explanation and discussion of the analysis of the molecular dynamic (MD) simulations as well as the implementation of the sticky-cation formalism. For the MD simulations presented here, we utilized the same molecular dynamics procedure and force fields used in Ref. 5.

A. Molecular Dynamics Simulation Methodology

Here we performed classical atomistic MD simulations using LAMMPS [6], following the methodology outlined in Ref. 5, which we will briefly recap. Our simulations were for LiTFSI in water at concentrations of 12 m and 15 m in a slit geometry in contact with charged interfaces.

We simulated the EDL of this WiSE in the NVT ensemble at 300 K, where the geometry of the cell was taken to be $33 \times 33 \times 266$ Å^3, with two $33 \times 33 \times 33$ Å^3 electrodes sandwiching the electrolyte region was made up of fixed Lennard Jones (LJ) spheres arranged in an fcc lattice (100). For 15 m, the box contained 636 ion pairs, 2356 water molecules, and 4096 electrode atoms. For 12 m, the box contained 588 ion pairs, 2725 water molecules, and 4096 electrode atoms. The initial configurations for all simulations were generated using the open-source software, PACKMOL [7]. Surface charges of ± 0.2 C/m^2 were applied by placing partial charges on the first layer of the electrode atoms. Additionally identical simulations were conducted for 15 m with surface charge of ± 0.15 C/m^2, ± 0.1 C/m^2, ± 0.05 C/m^2, and 0 C/m^2.

For all Li^+ and $TFSI^-$ we employed the CL&P force field [8]. For water, we employed the spc/e force field. Inter-atomic interactions are determined using Lorentz-Berthelot mixing rules. For the electrode, we did not explicitly model the dynamics, omitting the need for an 'electrode'-'electrode' force field. The electrode only interacts with the fluid through coulomb and Lennard-Jones interactions, which were made to be the same no matter what atom is interacting with the electrode atom with LJ well depth $\varepsilon = 0.001$eV and LJ well distance $\sigma = 3$ Å. Long-range electrostatic interactions were computed using the Particle-Particle Particle-Mesh (PPPM) solver with cut-off of 12 Å, which maps particle charge to a 2D mesh in the transverse direction for the nano-slit simulation[9].

Equilibration runs of about 12 ns (1 fs time steps) were performed initially with no applied potential/charge. Then the surface charge was ramped up from zero, allowing for 12 ns of equilibration, and 4 (2) ns of production at each electrode surface charge for 15 m (12 m) collecting frames every 4 (2) ps, giving a total of 1000 frames in each case.

B. Parameters for Theory and Analysis of Simulation Data

To model a given WiSE, one needs to obtain predictions for the volume ratios (ξ_+ & ξ_-), the functionality of the cations and anions (f_+ & f_-), and association constants (λ_{+-} & λ_{+0}). We will discuss how these are calculated, and then explain how quantities within the EDL are computed, before moving to screening lengths and integrated quantities. A detailed depiction of the analysis of the MD data is shown in Fig. S10.

The volume ratios can be determined by summing over the van der Waals spheres of the elements that comprise the molecules, ensuring not to double count for overlapping contributions, and normalizing this volume by the volume of water (v_0). In this work for the volume fractions, we use the same values found in Ref. 10, $\xi_+ = 0.4$ & $\xi_- = 10.8$.

To compute the associations in this MD simulation, we first identified the threshold distance for which an association would be classified. This can also be accomplished via studying the spatial distribution functions of the associating molecules and counting the number of "hot-spots" that are present [11]. Alternatively, one can utilize kinetic criteria [12] or machine learning methods [13] to define associations between species in the electrolyte. Following the previous analysis of this type of system [5], with the same simulation procedure and force fields, the association threshold was determined to be 2.7 Å. This means that if Li^+ is within 2.7 Å of an oxygen atom belonging to $TFSI^-$ or H_2O, an association was present. Note we do not consider any association between water and $TFSI^-$ as these are rare in WiSEs considered here [5, 10].

The coordination number of each Li^+ was obtained by counting the number of Li-O (Water) associations as well as Li-O ($TFSI^-$). Here, if multiple oxygens' from a $TFSI^-$ associate to a single Li^+, it was only counted once, i.e., bi-dentate or multi-dentate associations are only counted as one. This kind of procedure was also used to evaluate the number of associations each Li^+ had. Similarly, the number of Li^+ associated to a single water molecule's oxygen was extracted. Lastly, we repeated this procedure for the $TFSI^-$, but here, we only counted the associations by the number of unique Li^+ associated to the oxygen atoms belonging to the $TFSI^-$. In all these cases, the associations were assigned to the molecule's center of mass, which was calculated directly from the position data. This approximation was made to treat the individual molecules discretely and at one point in space. By doing this, however, it means the associations that are spread out over space are localized to the

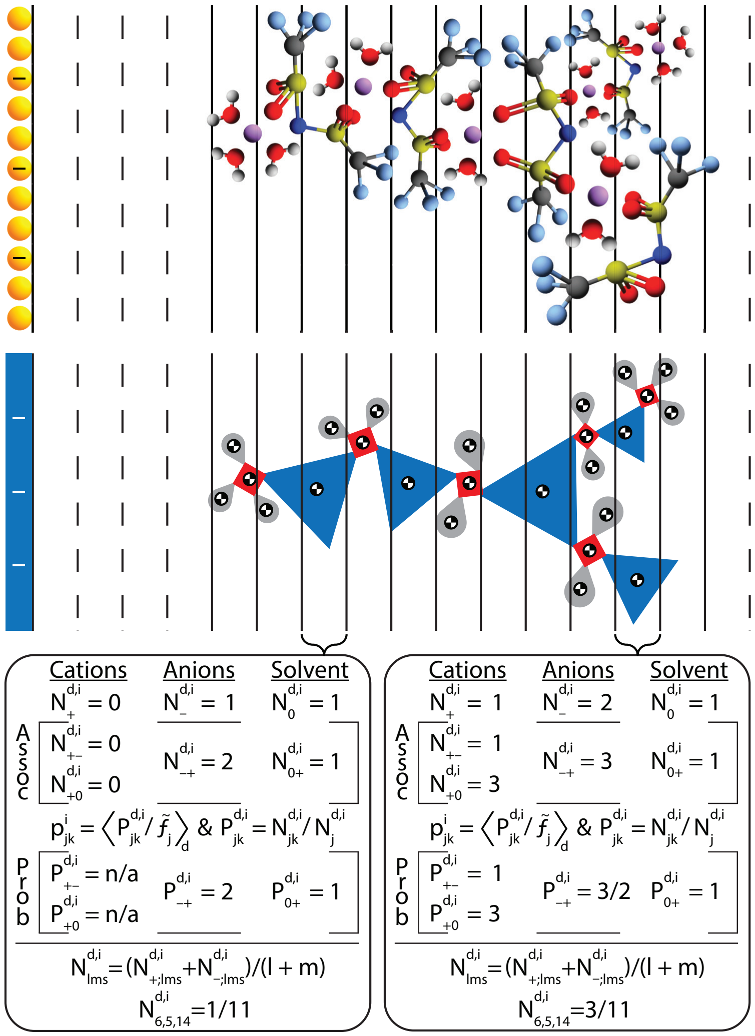

Figure S10. Schematic of Sample Molecular Dynamics Simulation Analysis. Here the top layer shows what a single cluster would look in our MD simulation. The second layer highlights how we decomposed the cluster, by localizing associations and positions to their center of masses. $\mathrm{N}_j^{d,i}$ indicates the number of species type j that are found in the ith single partition/bin for the dth data frame. $\mathrm{N}_{jk}^{d,i}$ represents the number of associations from species j to species k localized to the center of mass of species j. The number of species and associations can then be used by $\mathrm{N}_j^{d,i}/\mathrm{N}_{jk}^{d,i}$ to obtain the average association number $\mathrm{P}_{jk}^{d,i}$. This quantity is used to find the average association number per bin later by averaging it overall data frames and dividing by its functionality, $\tilde{f}_j$. Here we use $\tilde{f}_j$ since it can depart from the functionality when analyzing under different approximations like the sticky-cation formalism. Lastly, we calculate the approximate number density per bin of a lms-ranked cluster $\mathrm{N}_{lms}^{d,i}$ via the fraction of that clusters ionic backbone present in the bin.

center of mass.

Considering the total coordination number of Li^+ in the bulk, for 12 m and 15 m water-in-LiTFSI with electrodes with ± 0.2 C/m^2 surface charge in Fig. S11 and Fig. S12 respectively, one can note on average that they form more than four associations at both molalities. From this finding the Li^+ functionality should be $f_+ = \{4,5\}$. Previously in Ref. 10, they found that Li^+ in water-in-LiTFSI for a range of molalities had a coordination number slightly greater than four, leading to them choosing $f_+ = 4$. The functionality of the TFSI anions has been previously studied by analyzing their spatial distribution function and how their moieties interact with the lithium ions; it is set as $f_- = 3$ [10]. This comes from the partial negative charge distributed among the oxygen atoms of the $TFSI^-$ leading to the association being formed between Li-O. However, typically, when three Li^+ are associated with $TFSI^-$, it leads to two oxygen atoms being associated on a single Li^+. However, there are rare cases where four Li^+ can be associated with a single $TFSI^-$, but since they are rare, it is justifiable to set f_-=3.

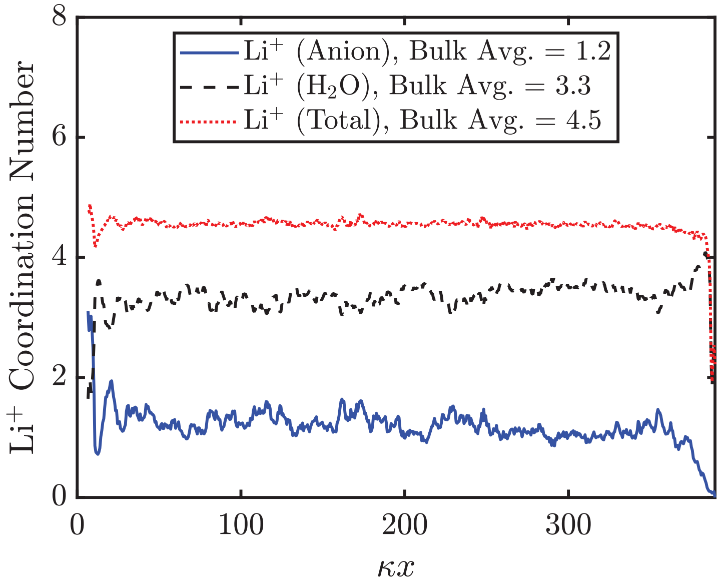

Figure S11. Lithium Coordination Number for 12 m water-in-LiTFSI for electrodes with $q_s = \pm 0.2$ C/m^2 surface charge. Here the left side of the plot corresponds to the interface with $q_s = 0.2$ C/m^2 and the right side with $q_s = -0.2$ C/m^2.

Following Ref. 10, we calculate the association probabilities through

$$p_{ij} = \left\langle \frac{\#\text{ of associations of type } ij}{f_i \cdot \#\text{ of molecules of type } i} \right\rangle \tag{S47}$$

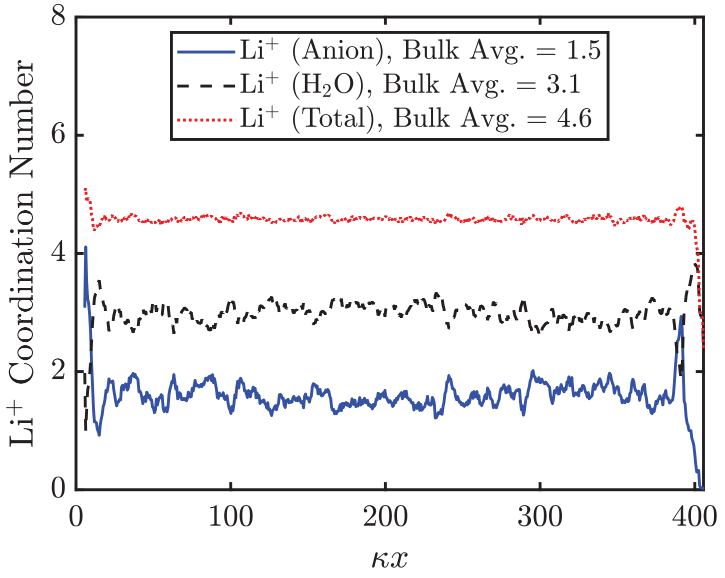

Figure S12. Lithium Coordination Number for 15 m water-in-LiTFSI for electrodes with $q_s = \pm 0.2$ C/m^2 surface charge. Here the left side of the plot corresponds to the interface with $q_s = 0.2$ C/m^2 and the right side with $q_s = -0.2$ C/m^2.

where there is an average temporally, and over space if in the bulk region. We also compute these probabilities as a function of position, where the normalization has to occur over only those bins/partitions that have species present. The process of extracting the association probabilities spatially is highlighted in Fig. S10. Note as shown a partition (or bin) can only produce a sample if the respective species are present, i.e., if no Li^+ is present, one cannot obtain a sampled value for $\bar{p}_{+-}$ and $\bar{p}_{+0}$. This makes sampling the association probabilities of the counter-ions in the EDL more challenging given the lower statistics available given their rare appearance close to the interface. In the sticky cation approximation, f_+ is replaced with the bulk coordination number of Li^+, to ensure the association probabilities are bounded with 0 and 1. Note, it is still possible to have a probability greater than one as uncommon coordination structures can exist in the EDL. An effect of this modification is that it slightly adjusts the extracted λ used for the sticky cation approximation.

From these association probabilities one can directly calculate the product of the ionic association probabilities, $\bar{p}_{+-}\bar{p}_{-+}$. Additionally, one can extract the variance of these sampled values over the production period. From this calculation and using the propagation of error, one can calculate the standard deviation for $\bar{p}_{+-}\bar{p}_{-+}$.

As discussed in the work of McEldrew *et al.* [10, 14], the association constants can be

obtained from Eqs. (S15)-(S16) using the previously computed ensemble average association probabilities. An equivalent procedure was utilized for the sticky case to extract λ using Eq. (S48) shown below,

$$\lambda = \frac{\lambda_{+-}}{\lambda_{+0}} = \frac{p_{-+}(1 - p_{0+})}{p_{0+}(1 - p_{-+})}. \tag{S48}$$

From this analysis we found that for 12 m $\lambda_{+-} = 24.1 \pm 0.66$ & $\lambda_{+0} = 106 \pm 1.7$ and 15 m $\lambda_{+-} = 36.9 \pm 1.7$ & $\lambda_{+0} = 159 \pm 5.18$. Additionally under the sticky cation approximation, we found that 12 m $\lambda = 0.226 \pm 0.0066$, 15 m $\lambda = 0.231 \pm 0.0066$. Alternatively, one can obtain predictions for these association constants without fitting from MD simulations by using integral equations and Wertheim's formalism [15–22]. The main drawback to this fitting-free approach is that can only be applied to certain simple cases [23].

To calculate the aggregates, we construct an adjacency matrix. This matrix represents the connectivity of the ionic species by labeling the species' local associations. Analyzing this adjacency matrix allows us to establish the *lms* rank of the cluster to which the ionic species and water belong. This information allows one to label each ionic species with its cluster identity; a similar treatment could be done for water or solvent molecules.

Next, we turn to describing in more detail how we calculate EDL quantities. As described above, we have outlined how the coordination numbers of Li^+ are dealt with, through localizing the associations on a Li^+. In what follows, we describe how we partition species and associations into bins as a function from the interface, which can then be compared against our theory.

Firstly, the volume fraction of each species was obtained by first calculating the average number of each species in a bin/partition. From this, we invoked the incompressibility compressibility constraint to normalize the total species volume by the total volume from the number density profiles to obtain the spatial species volume fractions profiles in the EDL. Here we selected bins to minimize the numerical artifacts of spatial profiles typically seen in larger bins. This leads to small bins with widths around 0.3 Å selected as a result of the overly small Debye length in these concentrated electrolytes. This partitioning is in line with previous studies [5].

An equivalent method was used to find the volume fraction of each cluster, where first, we obtained the average number of each type of cluster in each partition. Here, the cluster was segmented by the cations and anions that composed them as they can exist across multiple partitions; hence, accounting for the fractional amount of the cluster in each partition allowed

for a finer partition to be utilized. This is still an approximation as the bound water molecules are effectively shrink-wrapped to the ionic backbone in this method. Following the previous method, the volume of each cluster in the partition is normalized by the total volume in the partition, producing the volume fraction of each cluster.

To find the volume fraction of each cluster, where first, we obtained the average number of each type of cluster in each partition/bin. Here, the cluster was segmented by the cations and anions that composed them as they can exist across multiple partitions; hence, accounting for the fractional amount of the cluster in each partition allowed for a finer partition to be utilized. We achieved this here by counting what fraction of the ionic species that made up a cluster is found in that partition, highlighted in Fig. S10. If we required the partitions to contain the entire clusters, then only coarse partitioning of the MD simulation cell could be used. This is still an approximation as the bound water molecules are effectively shrink-wrapped to the ionic backbone of the cluster in this method, i.e. not explicitly counted for ion-containing clusters. Following the previous method, the volume of each cluster in the partition is normalized by the total volume in the partition, producing the volume fraction of each cluster. Note a more exacting way to analyze these clusters would be to forgo extracting the number density of the clusters initially. Through this approach, one would label all the species with the cluster to which they belong. Then, one can calculate what fraction of the volume in the partition they contribute from the total species volume fractions. Hence, this approach directly provides the volume fraction of each cluster in a partition without needing the number of densities. One can still extract the number densities by back-calculating from $\bar{\phi}_{lms}$ to $\bar{c}_{lms}$.

One needs to extract from the simulations the site size, which in the theory is set to the size of a water molecule (v_0). To determine this value, one first calculates the volume of the electrolyte simulation cell (V) from the initialization of the simulation cell. Then, by utilizing the incompressibility criteria, one can find,

$$1 = \phi_+ + \phi_- + \phi_0 = \frac{v_0}{V}\left(\xi_+ N_+ + \xi_- N_- + N_0\right). \tag{S49}$$

Since the number of molecules is known for each simulation's molality, and the electrolyte volume can be extracted, one can explicitly solve for v_0 from Eq. (S49). Using the volume ratios, as they are independent of the MD, the number of each species, and the volume of a single site (v_0), we can define the bulk species volume fractions for the simulations. This

composition represents the theory-equivalent bulk solution that can be tested against the simulation results.

From these volume fractions and the bulk dielectric constant found previously to be ϵ_r =10.1 for water-in-LiTFSI [5], one can calculate the Debye length (λ_D) for the simulations and the theory for which the distance from the charged interface is normalized by the inverse Debye length (κ). Performing this analysis for the 12 m and 15 m cases and comparing against theory, we found that for 12 m v_0 = 22.9 Å^3 with $\phi_+ \approx 0.0253$ and for 15 m v_0 = 22.5 Å^3 with $\phi_+ \approx 0.0268$. These values for the site volume roughly align with the volume of a single water molecule and fall roughly around that of the volume a bulk water molecule would take up, albeit slightly smaller, which is consistent with the more concentrated nature of the WiSEs.

To calculate the length scale of the aggregates, one can utilize the average volume fraction of each type of cluster in each partition to obtain $\bar{c}_{lms}$ in these partitions. As MD is restricted to finite domains, this restricts any potential gelation to existing as finite sized clusters, albeit with a large number of loops. Therefore we can explicitly use the pre-gel regime version of the length scale of the aggregates equation:

$$\ell_A^3 = \frac{v_0 \sum_{lms}(\xi_+ l + \xi_- m + s)^2 \bar{c}_{lms}}{\sum_{lms}(\xi_+ l + \xi_- m + s)\bar{c}_{lms}} = v_0 \sum_{lms}(\xi_+ l + \xi_- m + s)^2 \bar{c}_{lms}. \quad \text{(S50)}$$

Thus by explicitly doing these summations, the length scale of the aggregates in each partition can be calculated. For simplicity, these calculations were conducted using the pre-gel regime formula, although the oscillations in the EDL predict the segments of the system will gel. This treatment simplifies the comparison against the theory and experimental data.

Turning our attention to the integrated quantities. The excess surface concentrations, shown in Eq. (S51) where ∞ is the middle of the simulation cell, can be found by integrating the difference between the concentration of each species by their theoretical bulk reservoir, from the surface of each charged interface to the center of the simulation box. This analysis is done through spline interpolation and then integration of our numerical concentrations. Additionally, we exclude the depletion region where no species are present. This methodology allows us to compare these results directly against the theory without needing to implement a depletion region shift into the theory to make the extracted measurements have an equivalent meaning. Note that this treatment means that the left and right electrodes at no charge can produce different excess surface concentrations, and thus,

these two values are averaged to obtain the reported one.

$$\Gamma_i(q_s) = \frac{1}{v_0}\int_0^{\infty}\left(\bar{c}_i(x,q_s) - c_i^{\text{bulk}}\right)\,dx, \tag{S51}$$

Lastly to calculate the interfacial concentration of water, shown in Eq. (S52) where ℓ_w=5 Å one needs the bulk value of total water to normalize this quantity and the spatial profiles to integrate over. The bulk value was obtained from the middle third of the simulation cell, where we calculated the concentration of the total water molecules. Splines interpolate the profile to allow for numerical integration from the surface of the charged interface to 5 Å away from it. Enabling an analogous comparison against the MD prediction in the theory, the same depletion region in the MD is used in the theory, i.e. the region where no species are found near the charged interface. Since the theory was not developed to account for steric effects at the interfaces, which would create a deletion region, a synthetic one from the MD was incorporated. This shift was performed to enable a more direct comparison of the simulations and the theory's predictions. Lastly, as the shift values extracted at zero surface charge were not equal, their values were averaged.

$$\tilde{\rho}_{w,n}^{ads}(q_s) = \frac{\int_0^{\ell_w}\bar{c}_n(x,q_s)\,dx}{\ell_w c_0^{\text{bulk}}}. \tag{S52}$$

C. Stick Cation Testing and Validation for 12 m Water-in-LiTFSI

This section primarily focuses on the implications of making the sticky cation approximation and how it influences the theory and simulation results. For this reason, the discussion focuses mainly on the general trends and implications observed without going into fine details on the individual profiles in each of the figures as conducted in the main text. Presented here are the main EDL profiles at q_s = ±0.2 C/m^2 for 12 m water-in-LiTFSI, the general case plots are shown in Fig. S13 and Fig. S15, and the sticky cation approximation plots are shown in Fig. S14 and Fig. S16.

Initially, let us discuss what is expected to change between the general case and the sticky cation approximation. In the analysis of the MD simulation's data, the only change introduced by the sticky cation approximation is replacing f_+ with the bulk coordination number of Li^+, as discussed in the previous subsection. While this does not affect the extracted species volume fractions ($\bar{\phi}_i$) or the cluster volume fractions ($\bar{\phi}_{lms}$), it does impact the association probabilities of cations to anions ($\bar{p}_{+-}$) and cations to solvent ($\bar{p}_{+0}$). For

this reason, it is also expected to impact the product of the ionic association probabilities, $\bar{p}_{+-}\bar{p}_{-+}$, as well as λ, since it is calculated as a direct average instead of from the averages of λ_{+-} and λ_{+0}. In these three cases, it is expected to scale the curves by a factor of roughly $5/4.6 \approx 1.1$, which comes from the ratio of the general functionality $f_+ = 5$ to the bulk coordination number of Li^+ 4.6. Additionally, this change from the general case to sticky cation approximation will shift the critical $\bar{p}_{+-}\bar{p}_{-+} = (f_+ - 1)(f_- - 1)$ at which gelation is predicted to occur, owing to the different functionalities. In regards to the expected changes in the theory between these two cases, it is less clear, as it is governed by the solution to a system of polynomials with large order. However, one can loosely expect the trends and profiles predicted by the theory between the two cases to be roughly similar. However, the exact values may differ slightly as f_+ and λ are changing in addition to the enforcement of $\bar{p}_{+-} + \bar{p}_{+0} = 1$. The place of greatest change is expected to be in the association probabilities ($\bar{p}_{ij}$) profiles as in the sticky cation case $\bar{p}_{+-} + \bar{p}_{+0} = 1$ whereas in the general case this is not enforced. This can additional lead to changes in $\bar{p}_{+-}\bar{p}_{-+}$ if $\bar{p}_{+-}$ is strongly impacted. Similar to the changes in the simulations critical $\bar{p}_{+-}\bar{p}_{-+}$ threshold, the theory's threshold will change in the same manner.

Let us now consider the simulations for the negatively charged electrode. This can be done by comparing the simulation general case results shown in the left column of Fig. S13) against the sticky case results in the left column of Fig. S14). As expected, the species volume fraction profiles match exactly in Fig. S13.a) and Fig. S14.a). The cluster volume fraction profiles shown in Fig. S13.b) and Fig. S14.b) also match. Considering now Fig. S13.c) and Fig. S14.c) one can observe that the profiles for $\bar{p}_{-+}$ and $\bar{p}_{0+}$ are an exact match; however $\bar{p}_{+-}$ and $\bar{p}_{+0}$ in Fig. S14.c) appear to be the same profiles seen in Fig. S13.c) but scaled up by a constant. This difference is exactly as expected from the change in the MD data analysis. Lastly, one can note that the product of the ionic association probabilities, $\bar{p}_{+-}\bar{p}_{-+}$, shown in Fig. S14.d) is a scaled-up by a constant factor version of $\bar{p}_{+-}\bar{p}_{-+}$ in Fig. S13.d). Here, one can also note between Fig. S13.d) and Fig. S14.d) the critical threshold for $\bar{p}_{+-}\bar{p}_{-+}$ shifting up from the general case to the sticky cation case. Overall, the changes in the results and their profiles in the EDL do not appear to differ drastically between the general case and the sticky cation case in the MD simulations.

Next, let us consider how the theory's predictions change between the general case and the sticky cation case for the negatively charged electrode. This can be done by comparing

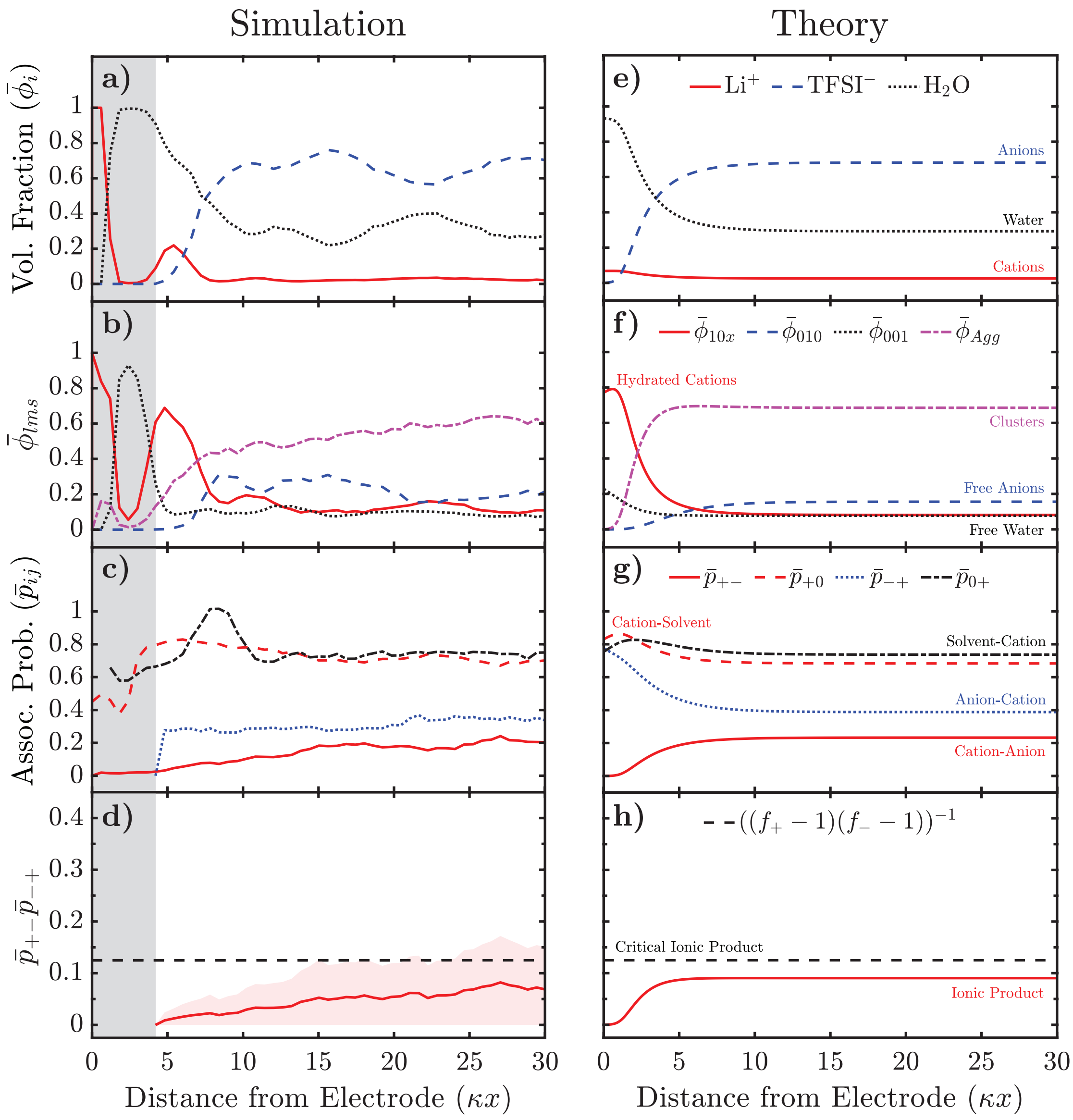

Figure S13. Distributions of properties of general 12m WiSEs in the EDL as a function from the interface, in dimensionless units, where κ is the inverse Debye length. a-d) are the results from MD simulations, and e-h) are the corresponding predictions from theory. The gray region indicates the minimum distance from the electrode at which a species was never found. a,e) Total volume fraction of each species. b,f) Volume fractions of hydrated cations, free anions, free water, and aggregates. c,g) Association probabilities. d,h) Product of the ionic association probabilities, $\bar{p}_{+-}\bar{p}_{-+}$, where the dashed line indicates the critical line for gelation. Here we use $f_+ = 5$, $f_- = 3$, $\xi_0 = 1$, $\xi_+ = 0.4$, $\xi_- = 10.8$, $\epsilon_r = 10.1$, $\lambda_{+-} = 24.1$, $\lambda_{+0} = 106$, $P = 4.995$ Debye, $v_0 = 22.9$ Å^3, and q$_s$ = -0.2 C/m^2.

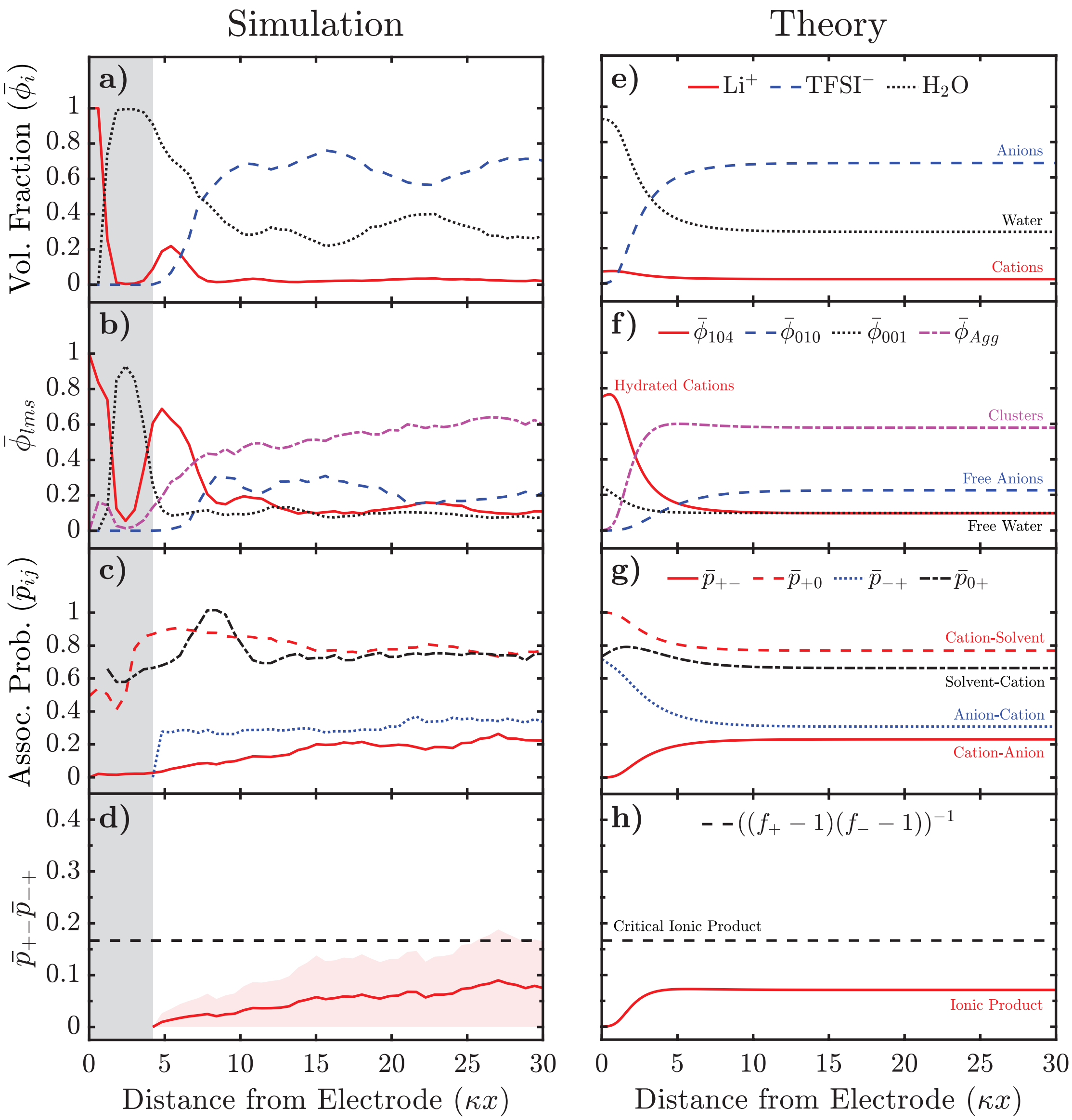

Figure S14. Distributions of properties of 12m WiSEs in the EDL using the sticky cation approximation as a function from the interface, in dimensionless units, where κ is the inverse Debye length. a-d) are the results from MD simulations, and e-h) are the corresponding predictions from theory. The gray region indicates the minimum distance from the electrode at which a species was never found. a,e) Total volume fraction of each species. b,f) Volume fractions of hydrated cations (Simulation $\bar{\phi}_{10x}$ & Theory $\bar{\phi}_{104}$), free anions, free water, and aggregates. c,g) Association probabilities. d,h) Product of the ionic association probabilities, $\bar{p}_{+-}\bar{p}_{-+}$, where the dashed line indicates the critical line for gelation. Here we use $f_+ = 4$, $f_- = 3$, $\xi_0 = 1$, $\xi_+ = 0.4$, $\xi_- = 10.8$, $\epsilon_r = 10.1$, $\lambda = 0.226$, $P = 4.995$ Debye, $v_0 = 22.9$ Å^3, and q$_s$ = -0.2 C/m^2.

the theory's general prediction shown in the right column of Fig. S13) to the right column of Fig. S14, which contains the theory's prediction for the sticky cation approximation. Viewing Fig. S13.e) and Fig. S14.e), one can compare the species volume fractions. In both Fig. S13.e) and Fig. S14.e), the Li^+ volume fraction profile as it approaches the interface appears to slowly increase before increasing faster then plateauing near the interface in nearly identical fashions. For the $TFSI^-$ volume fraction profile in both Fig. S13.e) and Fig. S14.e) appears to slowly decrease before rapidly decreasing close to the interface to a near zero value. In the case of H_2O volume fraction as it approaches the interface, it appears to slowly increase before rapidly increasing and plateauing near the interface in both cases as seen in Fig. S13.e) and Fig. S14.e). For the species volume fractions, both cases produced nearly identical predictions.

Considering the cluster volume fractions, one can note in both Fig. S13.f) and Fig. S14.f) that the strictly hydrated cation clusters slowly increase before rapidly increasing close to the interface before slightly decreasing from its peak value. Here while the trends are the same, the exact values close to the interface are a little different with the general case taking on slightly larger values compared to the sticky cation case. The volume fraction of bare anions ($\bar{\phi}_{010}$) appears to present the same trends in Fig. S13.f) and Fig. S14.f) where it is slowly decreasing before decreasing faster closer to the interface. Here, $\bar{\phi}_{001}$ appears to start at a notably higher value in the bulk in the sticky cation case compared to the general case, leading the sticky case's $\bar{\phi}_{010}$ profile appearing as a scaled-up version of the general case's $\bar{\phi}_{010}$ profile. The volume fraction of free water molecules ($\bar{\phi}_{001}$) seems to present the same trends in both Fig. S13.f) and Fig. S14.f) where it slow increases before quickly increasing near the interface. For the general case's $\bar{\phi}_{001}$, the profile seems to obtain a slightly lower value near the interface than in the sticky cation case. For the volume fraction of aggregates ($\bar{\phi}_{Agg}$) profile in the EDL, the two cases appear to have the same trends as displayed in Fig. S13.f) and Fig. S14.f) where it slow increases before achieving a local maximum after which it quickly decreases near the interface. For $\bar{\phi}_{Agg}$, one can note that the general case appears to start at a somewhat higher value in the bulk than in the sticky cation case. Additionally, the local maximum in $\bar{\phi}_{Agg}$ is slightly more notable in the sticky cation case compared to the general case. The trends and predictions for the cluster volume fractions appear to be similar between the general and the sticky cation cases, with minor deviations in the precise values.

Turning to the association probabilities displayed in Fig. S13.g) and Fig. S14.g) in both cases $\bar{p}_{+-}$ appears to slowly decrease before quickly decreasing towards zero close to the interface. The trends and values seen in the $\bar{p}_{+-}$ profile appear to agree in both the general and sticky cation cases. Considering now $\bar{p}_{+0}$ in the general case shown in Fig. S13.g), $\bar{p}_{+0}$ initially increases before quickly increasing close to the interface, then obtains its maximum value before decreasing slightly. In Fig. S14.g), $\bar{p}_{+0}$ in the sticky case also initially slowly increasing before rapidly increasing near the interface before plateauing around 1. This deviation between the general case and the sticky cation case is expected as $\bar{p}_{+-} + \bar{p}_{+0} = 1$ is enforced in the sticky cation case but not the general case, allowing for the more sophisticated profile in $\bar{p}_{+0}$ to exist independent of $\bar{p}_{+-}$ in the general case. Additionally, $\bar{p}_{+0}$ in the general case appears to take on a slightly lower value in the bulk compared to the sticky cation case. As seen in both Fig. S13.g) and Fig. S14.g), $\bar{p}_{-+}$ slowly increase before quickly increasing at a decelerating rate near the interface. While the trends match, the exact profiles appear to differ by scaling factor, i.e. the sticky case seems to be a slightly scaled-down version of the general case. Last for the association probabilities, $\bar{p}_{0+}$ demonstrated the same trends in its EDL profile as shown in both Fig. S13.g) and Fig. S14.g), where it is initially slowly increasing before increasing faster near the interface and achieving a maximum value before slightly decreasing. Once again while both the general and sticky cation trends are similar, here the sticky cation $\bar{p}_{0+}$ profile appears to be a scaled-down version of the general $\bar{p}_{0+}$ profile with a slightly more noticeable maximum. Overall, even with slight deviations between the association probability profiles in the general and sticky cation cases, they appear to produce similar trends and predictions.

Finally let us compare the product of the ionic association probabilities, $\bar{p}_{+-}\bar{p}_{-+}$, in the general and sticky cation cases shown in Fig. S13.h) and Fig. S14.h), respectively. In both cases $\bar{p}_{+-}\bar{p}_{-+}$ very slightly increases till obtaining a maximum after which is gradually decays to zero close to the interface. In Fig. S13.h) and Fig. S14.h), the trends for $\bar{p}_{+-}\bar{p}_{-+}$ are the same, but the exact value is slightly different with the general case taking on a slightly larger value in the bulk than the sticky cation case. Considering that trends in the predicted EDL quantities are rather close between the general and the sticky cation cases and that the slight deviations in values or trends do not significantly change the overarching predictions, this narrow difference suggests that the sticky cation approximation is reasonable for negatively charged electrodes.

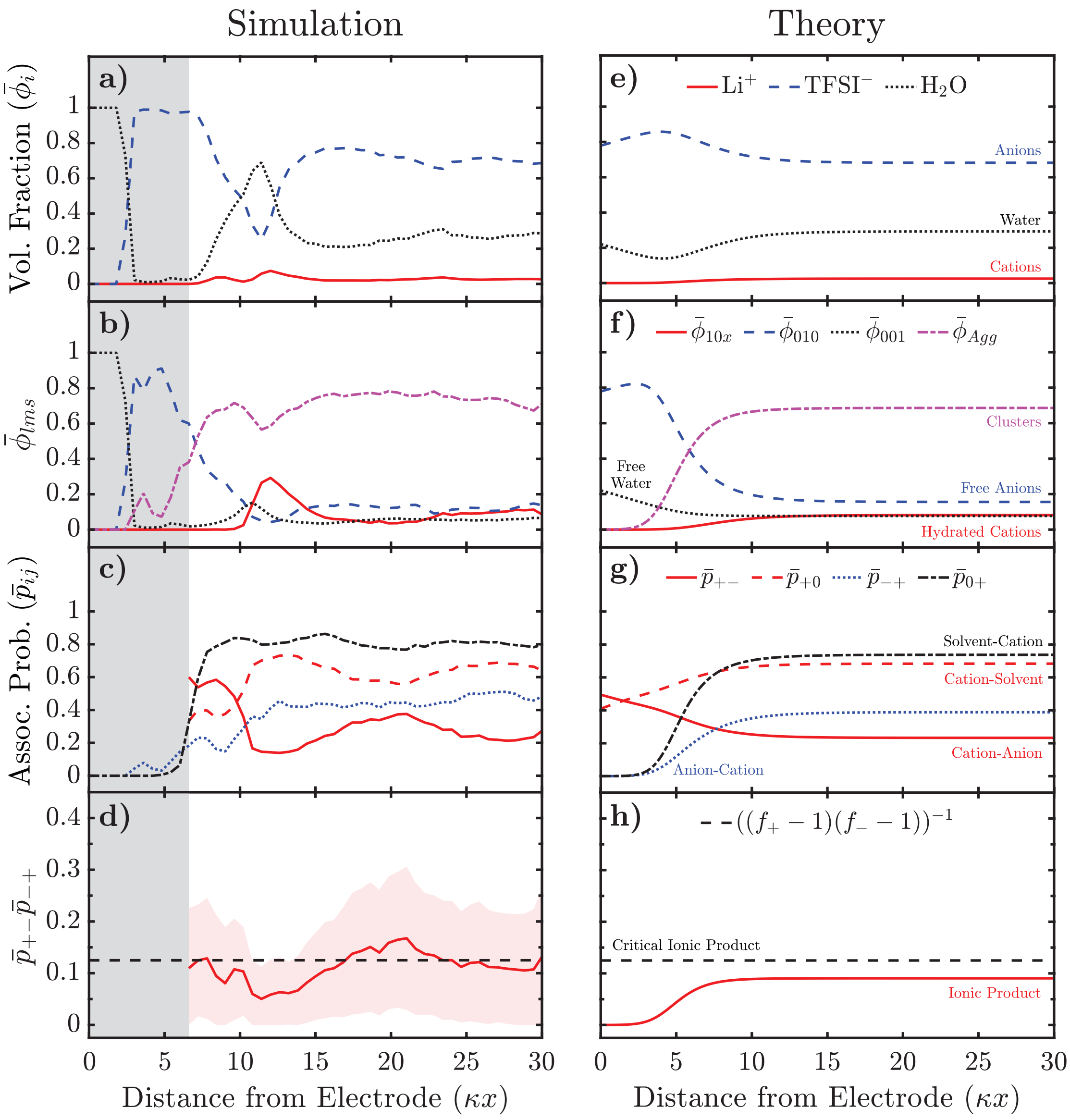

Figure S15. Distributions of properties of general 12m WiSEs in the EDL as a function from the interface, in dimensionless units, where κ is the inverse Debye length. a-d) are the results from MD simulations, and e-h) are the corresponding predictions from theory. The gray region indicates the minimum distance from the electrode at which a species was never found. a,e) Total volume fraction of each species. b,f) Volume fractions of hydrated cations, free anions, free water, and aggregates. c,g) Association probabilities. d,h) Product of the ionic association probabilities, $\bar{p}_{+-}\bar{p}_{-+}$, where the dashed line indicates the critical line for gelation. Here we use $f_+ = 5$, $f_- = 3$, $\xi_0 = 1$, $\xi_+ = 0.4$, $\xi_- = 10.8$, $\epsilon_r = 10.1$, $\lambda_{+-} = 24.1$, $\lambda_{+0} = 106$, $P = 4.995$ Debye, $v_0 = 22.9$ Å^3, and q$_s = 0.2$ C/m^2.

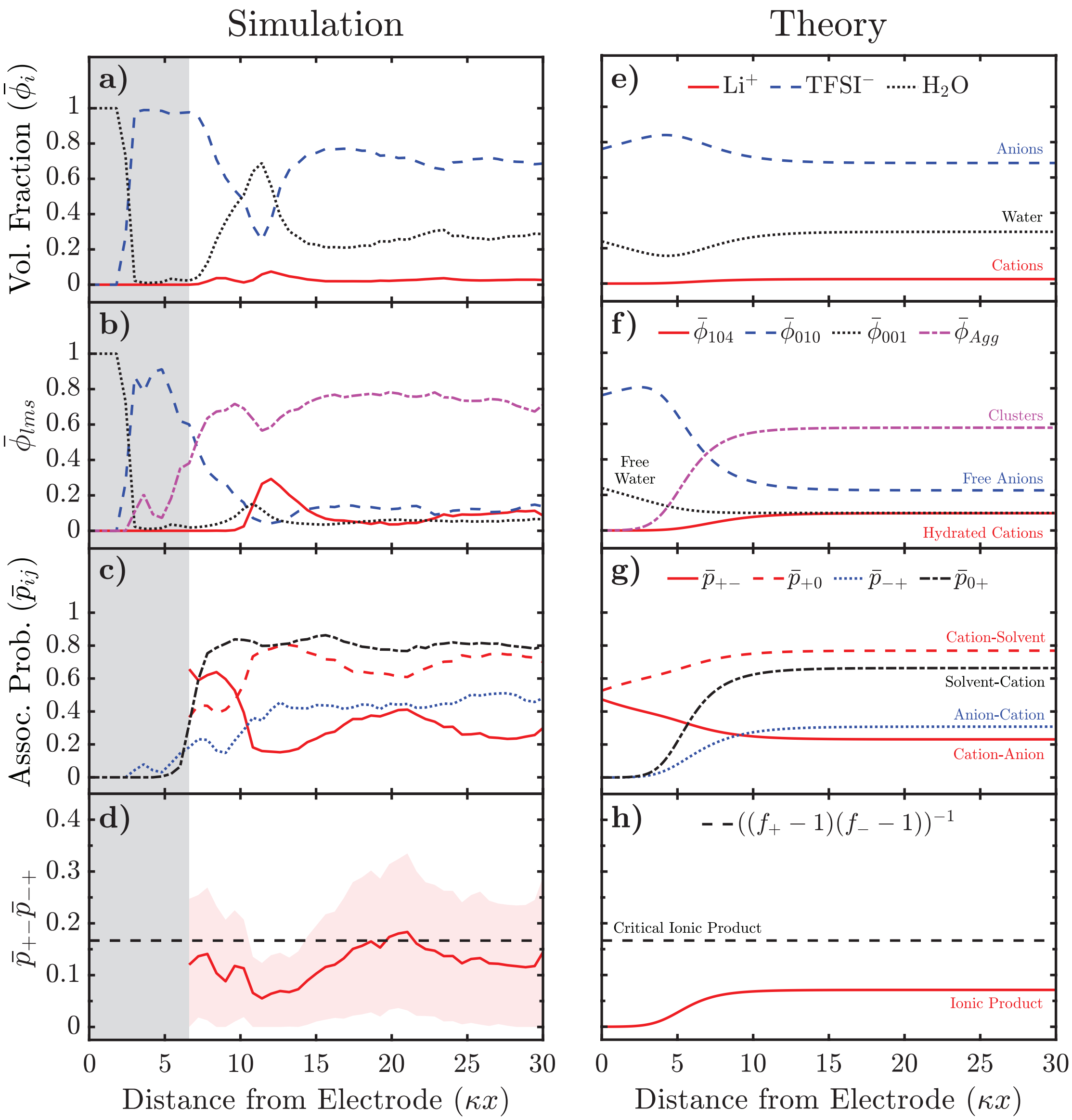

Figure S16. Distributions of properties of 12m WiSEs in the EDL using the sticky cation approximation as a function from the interface, in dimensionless units, where κ is the inverse Debye length. a-d) are the results from MD simulations, and e-h) are the corresponding predictions from theory. The gray region indicates the minimum distance from the electrode at which a species was never found. a,e) Total volume fraction of each species. b,f) Volume fractions of hydrated cations (Simulation $\bar{\phi}_{10x}$ & Theory $\bar{\phi}_{104}$), free anions, free water, and aggregates. c,g) Association probabilities. d,h) Product of the ionic association probabilities, $\bar{p}_{+-}\bar{p}_{-+}$, where the dashed line indicates the critical line for gelation. Here we use $f_+ = 4$, $f_- = 3$, $\xi_0 = 1$, $\xi_+ = 0.4$, $\xi_- = 10.8$, $\epsilon_r = 10.1$, $\lambda = 0.226$, $P = 4.995$ Debye, $v_0 = 22.9$ Å^3, and q$_s = 0.2$ C/m^2.

Now, let us consider the simulation measurements for the positively charged electrode. This can be done by comparing the simulation general case results shown in the left column of Fig. S15) against the sticky case results in the left column of Fig. S16). Similar to the negatively charged cases, the species volume fraction profiles in Fig. S15.a) and Fig. S16.a) are an exact match. Comparing Fig. S15.b) and Fig. S16.b), the cluster volume fractions are also an exact match. Turning to the association probabilities are shown in Fig. S15.c) and Fig. S16.c) one can note that the profiles for $\bar{p}_{-+}$ and $\bar{p}_{0+}$ as in the negatively charged case are exact matches; however as expected $\bar{p}_{+-}$ and $\bar{p}_{+0}$ in Fig. S16.c) appear to be scaled up by a constant factor compared to the $\bar{p}_{+-}$ and $\bar{p}_{+0}$ curves in Fig. S15.c). This change was expected to occur when the MD data analysis was changed from the general case to the sticky cation case. Lastly, considering the product of the ionic association probabilities, $\bar{p}_{+-}\bar{p}_{-+}$, displayed in Fig. S16.d) one can note the curve is a scaled-up version of $\bar{p}_{+-}\bar{p}_{-+}$ seen in Fig. S15.d). Once again, one can also note between Fig. S15.d) and Fig. S16.d) the critical threshold for $\bar{p}_{+-}\bar{p}_{-+}$ shifted up from the general case to the sticky cation case. As with the negatively charged electrode, the changes in the results and their profiles in the EDL do not appear to differ significantly between the general case and the sticky cation case in the MD simulations.

Let us consider how the theory's predictions change between the general case and the sticky cation case for the positively charged electrode. As before, we compare the theory's general prediction shown in the right column of Fig. S15) to the right column of Fig. S16, which contains the theory's prediction for the sticky cation approximation. Through Fig. S15.e) and Fig. S16.e), the predictions for species volume fractions can be compared. The Li^+ volume fraction ($\bar{\phi}_+$) profile is shown in Fig. S15.e) and Fig. S16.e), in both cases when approaching the interface $\bar{\phi}_+$ appears to slow decrease before decreasing faster near the interface. For the $TFSI^-$ volume fraction ($\bar{\phi}_-$) profile in both Fig. S15.e) and Fig. S16.e) it appears to slowly increase before quickly increasing closer to the interface and obtaining a maximum value around $4\lambda_D$ from the electrode, before moderately decreases. In the general case, $\bar{\phi}_-$ appears to have a slightly higher surface value than in the sticky cation case. In both cases, the H_2O volume fraction ($\bar{\phi}_0$), as it approaches the interface, appears to slowly decrease before quickly decreasing closer to the interface and obtaining a minimum around $4\lambda_D$ from the electrode before it moderately increases as seen in Fig. S15.e) and Fig. S16.e). Here, we see that in the general case, the $\bar{\phi}_0$ appears to take on a surface value

just marginally less than in the sticky cation case. For the species volume fractions, both cases produce similar trends with only slight deviations between the general case and the sticky cation approximation predictions.

For the cluster volume fractions, one can observe in both Fig. S15.f) and Fig. S16.f) that the strictly hydrated cation clusters slowly decrease before rapidly decreasing towards zero close to the interface. Here, the strictly hydrated cation clusters display the same trend and nearly identical profiles. The volume fraction of bare anions ($\bar{\phi}_{010}$) appears to present the same trends in Fig. S15.f) and Fig. S16.f) where it slowly increases before increasing faster near the interface where it achieves a maximum before decaying slightly to the interface. Here $\bar{\phi}_{001}$ appears to start at a higher value in the bulk and end at a slightly lower surface value in the sticky cation case than in the general case. The volume fraction of free water molecules ($\bar{\phi}_{001}$) seems to present the same trends in both Fig. S15.f) and Fig. S16.f) where it slow increases before quickly increasing near the interface. While the trends are the same, the exact surface $\bar{\phi}_{001}$ appears slightly larger in the sticky cation case compared to the general case. Lastly, for the volume fraction of aggregates ($\bar{\phi}_{Agg}$) profile in the EDL, the two cases appear to have the same trends as displayed in Fig. S15.f) and Fig. S16.f) where it slow decreases quickly decreasing towards zero closer to the interface. For $\bar{\phi}_{Agg}$, one can note that the general case appears to start at a somewhat higher value in the bulk than in the sticky cation case. The trends and predictions for the cluster volume fractions appear similar between the general and the sticky cation cases with only minor deviations.

The association probabilities are displayed in Fig. S15.g) and Fig. S16.g) in both cases for $\bar{p}_{+-}$ it appears to slowly increase before quickly increasing closer to the interface then increase slightly slower around $4\lambda_D$ from the interface. The trends seen in the $\bar{p}_{+-}$ profile appear to agree in both the general and sticky cation cases. The exact surface $\bar{p}_{+-}$ in the general case appears slightly larger than in the sticky cation approximation. In Fig. S15.g), $\bar{p}_{+0}$ shown in the general case initially decreases slowly before decreasing quickly close to the interface. In Fig. S16.g), $\bar{p}_{+0}$ displayed in the sticky case also initially slowly decreases before quickly decreasing closer to the interface and then decreasing slightly slower around $4\lambda_D$ from the interface. This deviation between the general and the sticky cation cases is expected as $\bar{p}_{+-} + \bar{p}_{+0} = 1$ is enforced in the sticky cation case but not the general case allowing for the structure of $\bar{p}_{+0}$ to not be a direct transformation of $\bar{p}_{+-}$ in the general case. Additionally, $\bar{p}_{+0}$ in the general case appears to take on a lower value in the bulk compared

to the sticky cation case. Shown in both Fig. S15.g) and Fig. S16.g) $\bar{p}_{-+}$ slowly decreasing before quickly decreasing towards zero near the interface. Here, the trends appear to match; however, the exact profiles appear to differ by scaling factor, i.e. the sticky case seems to be a slightly scaled-down version of the general case. Last for the association probabilities, $\bar{p}_{0+}$ demonstrated the same trends in its EDL profile as shown in both Fig. S15.g) and Fig. S16.g) where it is initially slowly decreasing before quickly decreasing towards zero near the interface. Once again while both the general and sticky cation trends are similar, here the sticky cation $\bar{p}_{0+}$ profile appears to be a scaled-down version of the general case's $\bar{p}_{0+}$ profile. Even with slight deviations between the association probability profiles in the general and sticky cation cases, they appear to produce similar trends and predictions.

Finally let us compare the product of the ionic association probabilities, $\bar{p}_{+-}\bar{p}_{-+}$, in the general and sticky cation cases shown in Fig. S15.h) and Fig. S16.h), respectively. In both cases, $\bar{p}_{+-}\bar{p}_{-+}$ very slightly decreases before gradually decays to zero close to the interface. In Fig. S15.h) and Fig. S16.h), the trends for $\bar{p}_{+-}\bar{p}_{-+}$ are the same, but the exact value is slightly different with the general case taking on a slightly larger value in the bulk than the sticky cation case. Given that the trends in the predicted EDL quantities are rather close between the general and the sticky cation cases and that the slight deviations in values or trends do not significantly change the overarching predictions, this finding suggests that the sticky cation approximation is reasonable for positively charged electrodes.

In conclusion as the results from MD simulations and the theory's predictions in the general case and under the sticky cation approximation do not vary significantly, this finding validates the usage of the approximation for this work for both negatively and positively charged electrodes. From the deviations observed, one can note that the accuracy of using this approximation may worsen at close proximity to the interface. This is expected as close to the interface, the system approaches limiting conditions for different species, which can significantly impact certain predictions from the theory, such as the association probabilities. Additionally, the theory is expected to deviate from the MD simulations as one closes the distance to the interface. In this region, one would expect to have a condensed layer in which the kinds of interactions and the form of species associations would vary significantly from the diffuse double layer. Additionally, sampling in the condensed layer may face a statistical shortfall that could be investigated and overcome by biased sampling methods, such as metadynamics simulations [24–26].

III. EXTENDED EXPERIMENTAL METHODS SECTION

A. Experimental Methods

Bis(trifluoromethane)sulfonimide lithium salt (LiTFSI, ≥99.0% (^{19}F-NMR), Sigma-Aldrich) was stored in a vacuum desiccator and dried in a vacuum oven at 95 °C for 24 h before use. Aqueous solutions of 1 m, 12 m, 15 m, and 21 m LiTFSI were prepared by dissolving salt in Milli-Q water (18.2 MΩ cm^{-1}) inside a N_2-filled anaerobic chamber (relative humidity controlled below 3% RH). Electrochemical measurements were performed in a sealed three-electrode cell with a gold disk electrode (2 mm diameter, CH Instruments) as working electrode, a gold wire (MSE Supplies) as counter electrode, and a silver wire (Sigma-Aldrich) as reference electrode. The gold disk electrode was polished mechanically on a microcloth polishing pad (Buehler) in 0.05 μm alumina particle (CH Instruments) slurry, rinsed with Deionized water and sonicated for 15 min, and blow-dried by ultrapure N_2 before use.

Electrochemical impedance spectroscopy measurements (EIS) were performed using a Gamry Reference 620 potentiostat. The impedance spectra were collected by applying an AC sinusoidal potential (10 mV) over a DC voltage in the frequency range from 0.1 Hz to 1 MHz. To eliminate hysteresis, the sequence of applied DC potential with 0.1 V step was chosen to start from open circuit potential (OCP) to positive or negative potential limits. Cyclic voltammetry measurements were taken with a scan rate of 10 mV s^{-1} after EIS measurements to confirm that the measurements were performed within the electrochemical stability window shown in Fig. S17. In our experiments, no unexpected or unusually high safety hazards were encountered.

B. Methods for Determining Differential Capacitance

Differential capacitance data were obtained by fitting the EIS data at each potential. Three methods were implemented based on the Nyquist plot and Cole-Cole plot; see Fig. S18. By examining the Nyquist plot in Fig. S18.a), we found that a circuit model with only a pure capacitor does not adequately fit the impedance data. Therefore, an electric circuit model with a constant phase element (CPE) was considered [27]. The impedance of a CPE

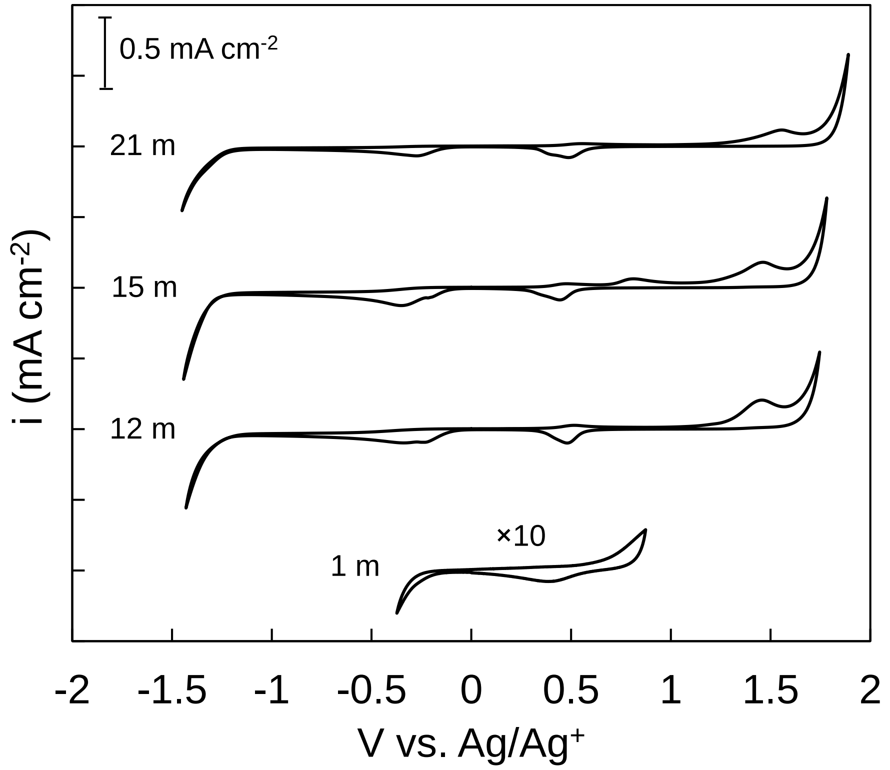

Figure S17. Cyclic voltammograms of 1 m, 12 m, 15 m, and 21 m LiTFSI on gold electrode measured after EIS with a scan rate of 10 mV s^{-1}. The current density for 1 m LiTFSI is plotted with ×10 magnification.

element (Z_{CPE}) can be described as,

$$Z_{CPE} = \frac{1}{Y_0(j\omega)^\alpha} = \frac{\cos(\alpha\pi/2)}{Y_0\omega^\alpha} - j\frac{\sin(\alpha\pi/2)}{Y_0\omega^\alpha}, \tag{S53}$$

where ω is angular frequency, Y_0[F $s^{\alpha-1}$] and α are the CPE parameters. α has a value such that $0 \leq \alpha \leq 1$; if $\alpha = 1$, the behavior is identical to a pure capacitor.

The equivalent circuit for fitting the Nyquist plot is shown in the inset of Fig. S18.a). Here, R_u represents the bulk electrolyte resistance, R_{DL} represents the double layer resistance, and CPE_{DL} describes the non-ideal double layer impedance. Only the double layer charging regime of the impedance spectra is selected for the fitting. The average α values of the fitted CPE element for each electrolyte across all the potentials are 0.930±0.004 for 1 m, 0.961±0.012 for 12 m, 0.965±0.008 for 15 m, and 0.960±0.010 for 21 m.

A more straightforward approach to determine the capacitive behavior is based on the complex capacitance plane shown in Fig. S18.b). The complex capacitance can be derived

from the impedance data,

$$C = C' + iC'' = \frac{1}{i\omega Z} = \frac{-Z''}{\omega\left((Z')^2 + (-Z'')^2\right)} - i\frac{Z'}{\omega\left((Z')^2 + (-Z'')^2\right)}. \tag{S54}$$

The experimental results show a distinctive capacitive process (slightly suppressed semicircle) at higher frequencies followed by a non-ideal behavior at lower frequencies. Capacitive behavior can also be indicated by a prominent peak in imaginary capacitance vs. frequency, see inset in Fig. S18.b); non-ideal slower processes lead to the increase of the imaginary capacitance at lower frequencies. The fast process (first semicircle in Cole-Cole plot/peak in -C”) has been attributed to double layer charging [28, 29]. The physical origin of the non-ideal behavior at lower frequencies is still not fully understood, but it is often associated with molecular mechanisms such as the reconstruction of the electrode surface, ion reorientation, and ion adsorption [28, 30, 31]. We selected the double layer charging frequency range for fitting as this experimental investigation focuses on its capacitance.

Method 1. Determining the capacitance at a selected frequency. As seen from Eq. (S53), a CPE element contributes to both the real and imaginary parts of impedance. However, only the imaginary part originates from the capacitive behavior. The capacitance can thus be derived from the imaginary component of the CPE [32]:

$$C(\omega) = \frac{1}{j\omega Z_{CPE,j}} = \frac{\mathrm{Y}_0\omega^{\alpha-1}}{\sin(\alpha\pi/2)}. \tag{S55}$$

The results display a frequency-dispersion of the capacitance, and therefore, a careful selection of the frequency to determine the capacitance is needed. Because the frequency range of double layer charging shifts significantly with water-in-LiTFSI electrolyte concentration, see inset in Fig. S18.b), using the same frequency to calculate the double layer capacitance across different concentrations of electrolytes is not ideal. Here, we select the peak-C” frequency to calculate the capacitance at each concentration.

Method 2. Deriving effective capacitance from circuit fitting parameters. This approach was first developed by Brug *et al.* [27] and explained in more detail by Hirschorn *et al.* [33]. Their methodology applies to cases with a distribution of surface time-constants where additive contributions are considered for each site of the electrode surface. The effective capacitance extracted is associated with a CPE and is expressed as,

$$C_{eff} = Y_0^{1/\alpha}\left(\frac{1}{R_u} + \frac{1}{R_{DL}}\right)^{(\alpha-1)/\alpha} = Y_0^{1/\alpha}\left(\frac{R_u R_{DL}}{R_u + R_{DL}}\right)^{(1-\alpha)/\alpha}. \tag{S56}$$

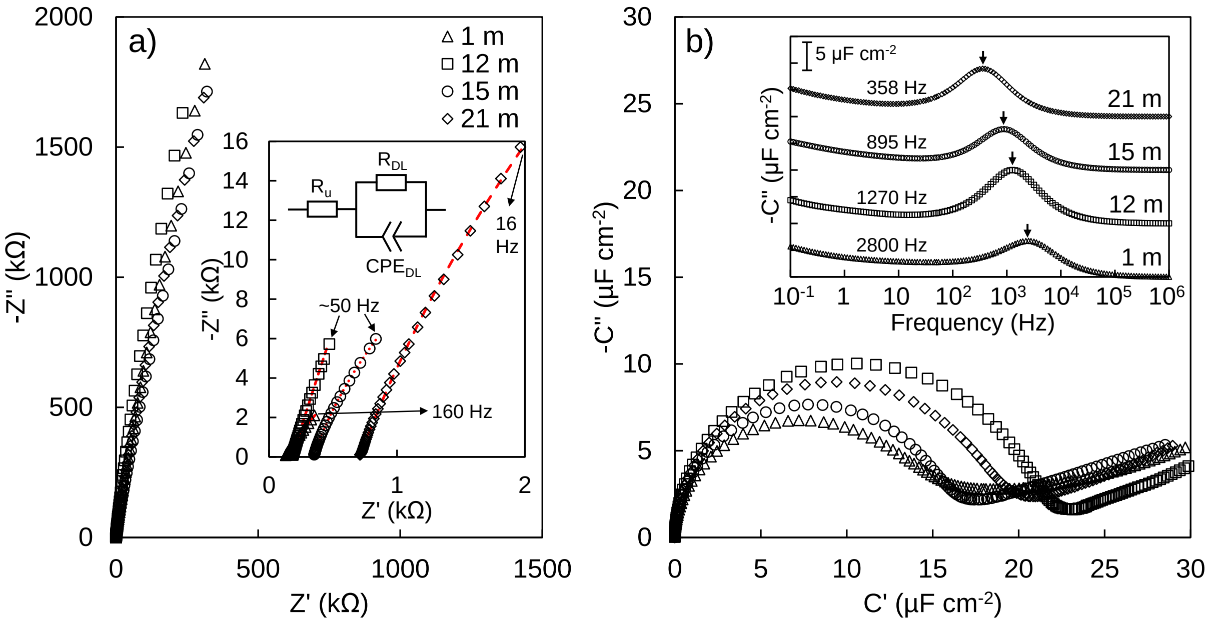

Figure S18. Nyquist plot and Cole-Cole (complex capacitance plane) plot for water-in-LiTFSI at open circuit potential. a) Nyquist plot and (inset) electric circuit fitting in the frequency range of double layer charging. The fitting circuit model is shown in the inset and the lower frequency limit for the fittings are labeled on the plot. The fitting curves are plotted in red. b) Cole-Cole plot and (inset) imaginary component of capacitance vs. frequency. A peak in the imaginary capacitance vs. frequency plot is associated with the capacitive process and the peak frequencies are noted by an arrow (↓) for each concentration on the plot.

When $R_{DL} \gg R_u$, Eq. (S56) simplifies to,

$$C_{eff} = Y_0^{1/\alpha} (R_u)^{(1-\alpha)/\alpha}. \tag{S57}$$

Method 3. Cole-Cole fit. Instead of analyzing data in the impedance plane, the capacitance plane is used for fitting. In the complex capacitance plane, the fitting can be performed using the Cole-Cole function:

$$C(\omega) = \frac{C}{1 + (i\omega\tau)^{\alpha}} \tag{S58}$$

Where τ denotes the relaxation time and α indicates the ideality of the capacitive process.

If multiple capacitive processes are observed, each process i is assumed to occur in parallel and the total capacitive process can be described as follows,

$$C(\omega) = \sum_i \frac{C_i}{1 + (i\omega\tau)^{\alpha_i}}. \tag{S59}$$

Here, we only fitted the fast capacitive process (1st semicircle) attributed to the double layer charging.

The extracted differential capacitances are shown in Fig. S19 and Fig. S20. The capacitance values derived from Cole-Cole methods are slightly higher than from the other two methods based on fitting the Nyquist plot, which is consistent with the comparison of analysis methods reported by Small *et al.* [34]. But overall, the three methods deliver very similar results.

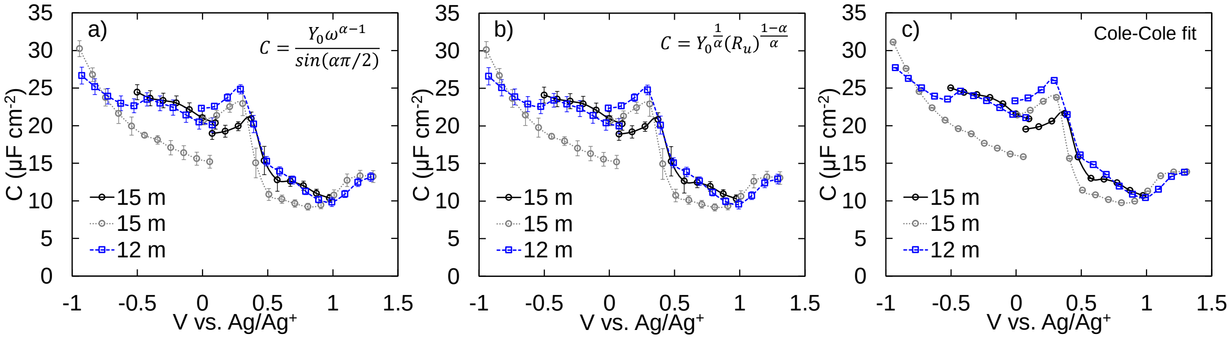

Figure S19. Differential capacitance of 15 m and 12 m water-in-LiTFSI as a function of the applied voltage from EIS measurements. The differential capacitance was obtained using three different fitting methods: a) Capacitance at a selected frequency, b) Capacitance derived from circuit fitting parameters, and c) Capacitance determined by the Cole-Cole method. Two measurements were taken for both the positive and negative branches at the concentration of 15 m LiTFSI, respectively, which are shown in the three plots for comparison.

Our current experimental findings highlight the function of electrode material and surface roughness that may play a key role in the measured differential capacitance [35–40]. In Zhang *et al.* [41], their differential capacitance measurements for 21m water-in-LiTFSI are much higher at negative potentials than these results with a similar structure as shown here in Fig. S20.b). The difference in measurements could be due to the surface preparation or EIS protocol and fitting methods used. Since Zhang *et al.* [41] displays similar trends, the different methodology used to analyze the data could explain the quantitative differences.

C. Additional Theory-Experimental Comparison

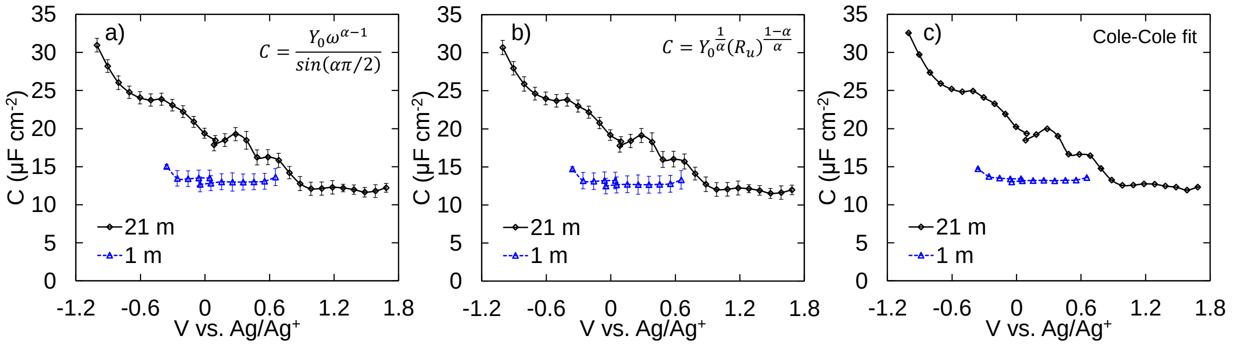

Figure S20. Differential capacitance of 21 m and 1 m aqueous LiTFSI as a function of the applied voltage from EIS measurements. The differential capacitance was obtained using three different fitting methods: a) Capacitance at a selected frequency, b) Capacitance derived from circuit fitting parameters, and c) Capacitance fitted by the Cole-Cole method.

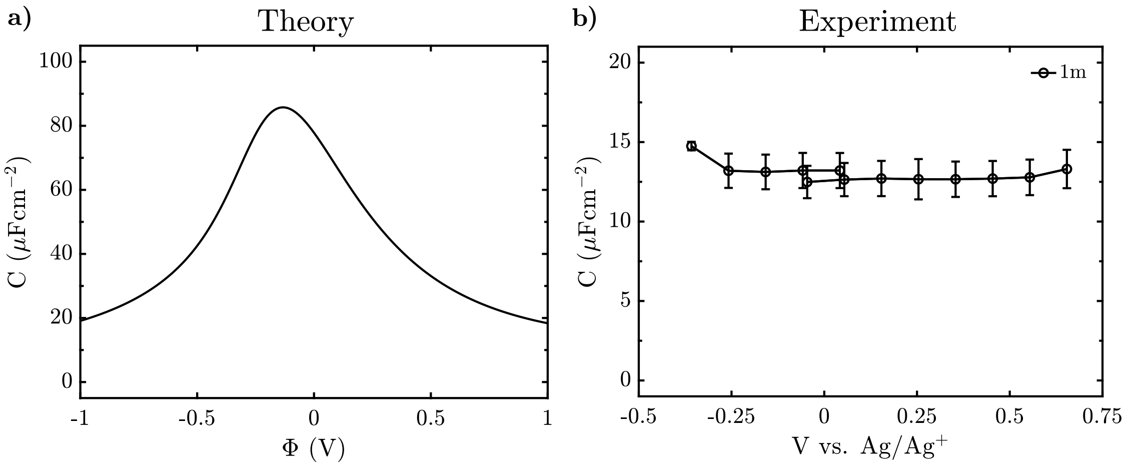

Figure S21. Differential Capacitance Comparison for 1m water-in-LiTFSI. a) Theory prediction for the differential capacitance of water-in-LiTFSI as a function of the electrostatic potential using $f_+ = 4$, $f_- = 3$, $\xi_0 = 1$, $\xi_+ = 0.4$, $\xi_- = 10.8$, $\epsilon_r = 10.1$, $\lambda = 0.228$, $P = 4.995$ Debye, $v_0 = 22.7$ Å^3, and $\alpha = 0.1$. b) Experimental measurement of the differential capacitance of water-in-LiTFSI derived from circuit fitting parameters as a function of the applied electrostatic potential.

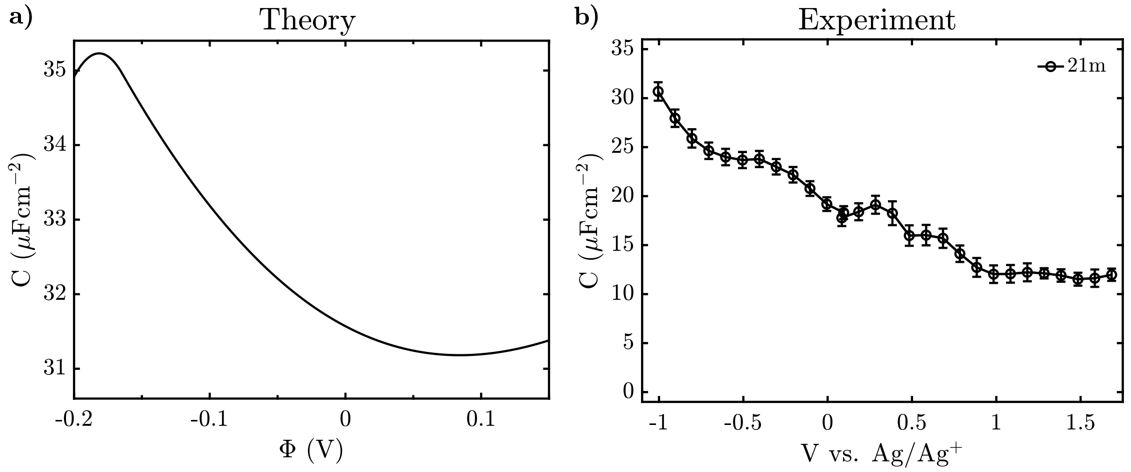

Figure S22. Differential Capacitance Comparison for 21m water-in-LiTFSI. a) Theory prediction for the differential capacitance of water-in-LiTFSI as a function of the electrostatic potential using $f_+ = 4$, $f_- = 3$, $\xi_0 = 1$, $\xi_+ = 0.4$, $\xi_- = 10.8$, $\epsilon_r = 10.1$, $\lambda = 0.228$, $P = 4.995$ Debye, $v_0 = 22.7$ Å^3, and $\alpha = 0.1$. b) Experimental measurement of the differential capacitance of water-in-LiTFSI derived from circuit fitting parameters as a function of the applied electrostatic potential.

[1] Z. A. Goodwin, G. Feng, and A. A. Kornyshev, Electrochim. Acta **225**, 190 (2017).

[2] Z. A. Goodwin, M. McEldrew, J. P. de Souza, M. Z. Bazant, and A. A. Kornyshev, J. Chem. Phys. **157**, 094106 (2022).

[3] R. Descartes, *La géométrie* (1637).

[4] D. M. Markiewitz, Z. A. Goodwin, M. McEldrew, J. P. de Souza, X. Zhang, R. M. Espinosa-Marzal, and M. Z. Bazant, Faraday Discussions , 365 (2024).

[5] M. McEldrew, Z. A. Goodwin, A. A. Kornyshev, and M. Z. Bazant, The journal of physical chemistry letters **9**, 5840 (2018).

[6] S. Plimpton, J. Comput. Phys. **117**, 1 (1995).

[7] L. Martínez, R. Andrade, E. G. Birgin, and J. M. Martínez, J. Comput. Chem. **30**, 2157 (2009).

[8] J. N. C. Lopes and A. A. Pádua, Theoretical Chemistry Accounts **131**, 1129 (2012).

[9] R. W. Hockney and J. W. Eastwood, *Computer simulation using particles* (CRC Press, 1988).

[10] M. McEldrew, Z. A. Goodwin, S. Bi, A. Kornyshev, and M. Z. Bazant, J. Electrochem. Soc. **168**, 050514 (2021).

[11] M. McEldrew, Z. A. H. Goodwin, H. Zhao, M. Z. Bazant, and A. A. Kornyshev, J. Phys. Chem B **125**, 2677–2689 (2021).

[12] G. Feng, M. Chen, S. Bi, Z. A. Goodwin, E. B. Postnikov, N. Brilliantov, M. Urbakh, and A. A. Kornyshev, Phys. Rev. X **9**, 021024 (2019).

[13] P. Jones, F. Coupette, A. Härtel, *et al.*, The Journal of Chemical Physics **154**, 134902 (2021).

[14] M. McEldrew, Z. A. Goodwin, S. Bi, M. Z. Bazant, and A. A. Kornyshev, J. Chem. Phys. **152**, 234506 (2020).

[15] M. S. Wertheim, Journal of statistical physics **35**, 19 (1984).

[16] M. S. Wertheim, Journal of statistical physics **35**, 35 (1984).

[17] M. Wertheim, Journal of statistical physics **42**, 459 (1986).

[18] M. Wertheim, Journal of statistical physics **42**, 477 (1986).

[19] D. Laría, H. R. Corti, and R. Fernández-Prini, Journal of the Chemical Society, Faraday Transactions **86**, 1051 (1990).

[20] L. Blum and O. Bernard, Journal of statistical physics **79**, 569 (1995).

[21] J.-P. Simonin, O. Bernard, and L. Blum, The Journal of Physical Chemistry B **103**, 699 (1999).

[22] F. Sciortino, E. Bianchi, J. F. Douglas, and P. Tartaglia, The Journal of chemical physics **126** (2007).

[23] Z. A. H. Goodwin, M. McEldrew, B. Kozinsky, and M. Z. Bazant, PRX Energy **2**, 013007 (2023).

[24] A. Barducci, M. Bonomi, and M. Parrinello, Wiley Interdisciplinary Reviews: Computational Molecular Science **1**, 826 (2011).

[25] O. Valsson, P. Tiwary, and M. Parrinello, Annual review of physical chemistry **67**, 159 (2016).

[26] J. Hénin, T. Lelièvre, M. R. Shirts, O. Valsson, and L. Delemotte, arXiv preprint arXiv:2202.04164 (2022).

[27] G. Brug, A. L. van den Eeden, M. Sluyters-Rehbach, and J. H. Sluyters, Journal of electroanalytical chemistry and interfacial electrochemistry **176**, 275 (1984).

[28] M. Drüschler, B. Huber, and B. Roling, The Journal of Physical Chemistry C **115**, 6802 (2011).

[29] J. M. Klein, E. Panichi, and B. Gurkan, Physical Chemistry Chemical Physics **21**, 3712 (2019).

[30] B. Roling, M. Drüschler, and B. Huber, Faraday discussions **154**, 303 (2012).

[31] P. Reichert, K. S. Kjær, T. B. van Driel, J. Mars, J. W. Ochsmann, D. Pontoni, M. Deutsch, M. M. Nielsen, and M. Mezger, Faraday discussions **206**, 141 (2018).

[32] V. Lockett, R. Sedev, J. Ralston, M. Horne, and T. Rodopoulos, The Journal of Physical Chemistry C **112**, 7486 (2008).

[33] B. Hirschorn, M. E. Orazem, B. Tribollet, V. Vivier, I. Frateur, and M. Musiani, Electrochimica acta **55**, 6218 (2010).

[34] L. J. Small and D. R. Wheeler, Journal of The Electrochemical Society **161**, H260 (2014).

[35] T. Pajkossy, Journal of Electroanalytical Chemistry **364**, 111 (1994).

[36] V. Lockett, M. Horne, R. Sedev, T. Rodopoulos, and J. Ralston, Physical Chemistry Chemical Physics **12**, 12499 (2010).

[37] T. Jänsch, J. Wallauer, and B. Roling, The Journal of Physical Chemistry C **119**, 4620 (2015).

[38] O. Oll, T. Romann, C. Siimenson, and E. Lust, Electrochemistry Communications **82**, 39 (2017).

[39] S. Torabi, M. Cherry, E. A. Duijnstee, V. M. Le Corre, L. Qiu, J. C. Hummelen, G. Palasantzas, and L. J. A. Koster, ACS applied materials & interfaces **9**, 27290 (2017).

[40] T. Aslyamov, K. Sinkov, and I. Akhatov, Physical Review E **103**, L060102 (2021).

[41] R. Zhang, M. Han, K. Ta, K. E. Madsen, X. Chen, X. Zhang, R. M. Espinosa-Marzal, and A. A. Gewirth, ACS Applied Energy Materials **3**, 8086 (2020).
